# Epitopes of Microbial and Human Heat Shock Protein 60 and Their Recognition in Myalgic Encephalomyelitis

**DOI:** 10.1371/journal.pone.0081155

**Published:** 2013-11-28

**Authors:** Amal Elfaitouri, Björn Herrmann, Agnes Bölin-Wiener, Yilin Wang, Carl-Gerhard Gottfries, Olof Zachrisson, Rϋdiger Pipkorn, Lars Rönnblom, Jonas Blomberg

**Affiliations:** 1 Section of Clinical Microbiology, Department of Medical Sciences, Uppsala University, Uppsala, Sweden; 2 Gottfries Clinic AB, Mölndal, Sweden; 3 Deutsches Krebsforschungszentrum, Heidelberg, Germany; 4 Section of Rheumatology, Department of Medical Sciences, Uppsala University, Uppsala, Sweden; San Raffaele Scientific Institute, Italy

## Abstract

Myalgic encephalomyelitis (ME, also called Chronic Fatigue Syndrome), a common disease with chronic fatigability, cognitive dysfunction and myalgia of unknown etiology, often starts with an infection. The chaperonin human heat shock protein 60 (HSP60) occurs in mitochondria and in bacteria, is highly conserved, antigenic and a major autoantigen. The anti-HSP60 humoral (IgG and IgM) immune response was studied in 69 ME patients and 76 blood donors (BD) (the Training set) with recombinant human and *E coli* HSP60, and 136 30-mer overlapping and targeted peptides from HSP60 of humans, *Chlamydia*, *Mycoplasma* and 26 other species in a multiplex suspension array. Peptides from HSP60 helix I had a chaperonin-like activity, but these and other HSP60 peptides also bound IgG and IgM with an ME preference, theoretically indicating a competition between HSP60 function and antibody binding. A HSP60-based panel of 25 antigens was selected. When evaluated with 61 other ME and 399 non-ME samples (331 BD, 20 Multiple Sclerosis and 48 Systemic Lupus Erythematosus patients), a peptide from *Chlamydia pneumoniae* HSP60 detected IgM in 15 of 61 (24%) of ME, and in 1 of 399 non-ME at a high cutoff (p<0.0001). IgM to specific cross-reactive epitopes of human and microbial HSP60 occurs in a subset of ME, compatible with infection-induced autoimmunity.

## Introduction

Chaperonins are common autoantigens. HSP60 is unique in both being highly conserved among pro- and eukaryotes [1,2] and highly antigenic [2]. Multiple Sclerosis (MS) patients have autoantibodies (predominantly IgM) to chaperonins like HSP60, HSP70 and alpha-B-crystallin [3–5]. Antibodies to peptides from human HSP60 are also common in type 1 diabetes (T1D) [6]. Antibodies to a microbial HSP60 are likely to crossreact with human HSP60, which also can crossreact with other human proteins [7]. 

Myalgic encephalomyelitis (ME) also referred to as the “chronic fatigue syndrome” (CFS; in the following just referred to as ME), is a common chronic debilitating disease. ME is characterized by chronic fatigability, cognitive dysfunction and myalgia. Numerous investigations have also demonstrated an unexplained long-term chronic inflammation and immune dysfunction in some ME patients [8], including autoimmunity [9–14]. A mitochondrial dysfunction in ME patients has been reported [15], but needs further investigation. ME overlaps with the clinical entities fibromyalgia (FM) and irritable bowel syndrome (IBS). ME often appears after a severe viral or bacterial infection. Human herpesvirus 6 (HHV-6) [16–19], Epstein-Barr virus (EBV)[19–29], enteroviruses [30–32], parvovirus B19 [32,33], *Chlamydia pneumoniae* [34,35] and *Mycoplasma* spp. [36–40] have been implicated in, but not proven to be the single cause of, ME. For a review see e.g. 41. Although there are limitations to interpretation of infection serology, it is in practice often possible to assess past or present microbial activity by studying the levels of IgG and IgM directed to the microbe. The present investigation sprung out of a large effort to find ME-selective serological markers. Over 1000 viral, bacterial and protozoal antigens (whole microbes, recombinant proteins and synthetic peptides) were tested (not shown). We then observed a tendency for HSP60 antigens to react selectively with ME samples compared to controls. This was the reason for the present study, where SMIA (Suspension Multiplex ImmunoAssay) was used to simultaneously detect IgG and IgM antibodies to recombinant and 30-mer peptide heat shock protein 60 (HSP60) antigens from humans and many microbes [42] in ME and controls. We found a major epitope which overlapped the peptide binding I helix of HSP60. Some of the HSP60 epitopes preferentially bound antibodies from ME samples. When tested in an independent evaluation set of 61 ME patients, one of the antigens reacted preferentially with ME, but not as much with BD, MS and SLE samples. 

Abnormal HSP60 levels, and an abnormal HSP60 response to exercise, have been found in ME patients [43]. The effect of HSP60 antibodies on human HSP60 and mitochondrial functions, and how a possible harmful effect from such antibodies normally is avoided, remains to be determined. Our results demonstrate HSP60 epitopes recognized by the immune system of healthy individuals, more by IgM than by IgG antibodies, as well as a selective immune response directed against a few HSP60 epitopes in a subset of ME patients. It is likely that HSP60 antibody reactions due to infection promote formation of autoantibodies to human HSP60. Whether that has any relevance for the pathogenesis and diagnosis of ME is uncertain. 

## Results

### Epitope definition stage

The known autoantigenicity of human HSP60, the high antigenicity of microbial HSP60, and reports on mitochondrial dysfunction in ME, led us to test whether ME patients had higher levels of antibody to HSP60 than blood donor controls. 

Anti-HSP60 antibodies in healthy humans and in ME patients: 69 ME and 76 blood donor (BD) samples (the Training set) were tested against bead-coupled recombinant human and *E. coli* HSP60 (Table 1). A relatively small but significant difference between ME and BD was observed in the IgG tests for human, and *E. coli* HSP60. The difference between ME and BD became more marked in the IgM test with human HSP60. 

**Table 1:** Antibody reactivity of HSP60-derived antigens*.

**Set of samples**	**Antigen**	**Antibody type**							
		**IgG**				**IgM**			
		Number of reactive above cutoff		P value, Fisher exact test	P value, Wilcoxon rank sum test	Number of reactive above cutoff		P value, Fisher exact test	P value, Wilcoxon rank sum test
**Training set**									
		ME (n=69)	BD (n=76)		ME vs BD	ME (n=69)	BD (n=76)		ME vs BD
	**Recombinant proteins**								
	Human HSP60	7	1	0.027, co 4000 MFI	n.s.	22	6	0.0003, co 200 MFI	<0.005
	E coli GroEl	36	15	<0.0001, co 4000 MFI	n.s.	17	5	0.0045, co 200 MFI	<0.005
	**Synthetic HSP60 peptides**								
	Selected human peptides								
	G20	7	1	0.0027, co 200 MFI	<0.01	43	8	<0.0001, co 200 MFI	<0.005
	G20c	19	16	n.s., co 200 MFI	0.014	39	9	<0.0001, co 200 MFI	<0.005
	Selected microbial G20c homologs								
	*Staphylococcus aureus*	54	47	n.s., co 200 MFI	<0.01	8	0	0.0021, co 200 MFI	<0.005
	*Chlamydia pneumoniae*	49	17	<0.0001,co 200 MFI	<0.001	35	1	<0.0001, co 200 MFI	<0.005
	*Plasmodium falciparum*	66	54	n.s., co 200 MFI	<0.01	56	3	<0.0001, co 200 MFI	<0.005
	Other selected microbial peptides								
	*Chlamydia pneumoniae* C18, short spacer	34	14	<0.0001, co 200 MFI	n.s.	54	9	<0.0001, co 200 MFI	
	*Chlamydia pneumoniae*, E25, long spacer	1	0	n.s., co 200 MFI	n.s.	18	1	<0.0001, co 200 MFI	
	*Mycoplasma penetrans* D25	19	1	<0.0001, co 200 MFI	<0.01	16	3	0.0009, co 200 MFI	
**Evaluation set**						ME (n=61)	BD (n=331)	ME vs BD	
	*Chlamydia pneumoniae* HSP60 G20c homolog	not done	not done			25	12	<0.0001, co 2 rel MFI	<0.01
	*Chlamydia pneumoniae* HSP60 G20c homolog	not done	not done			12	1	<0.0001, co 3.2 rel MFI	<0.01

**Figure 1:**
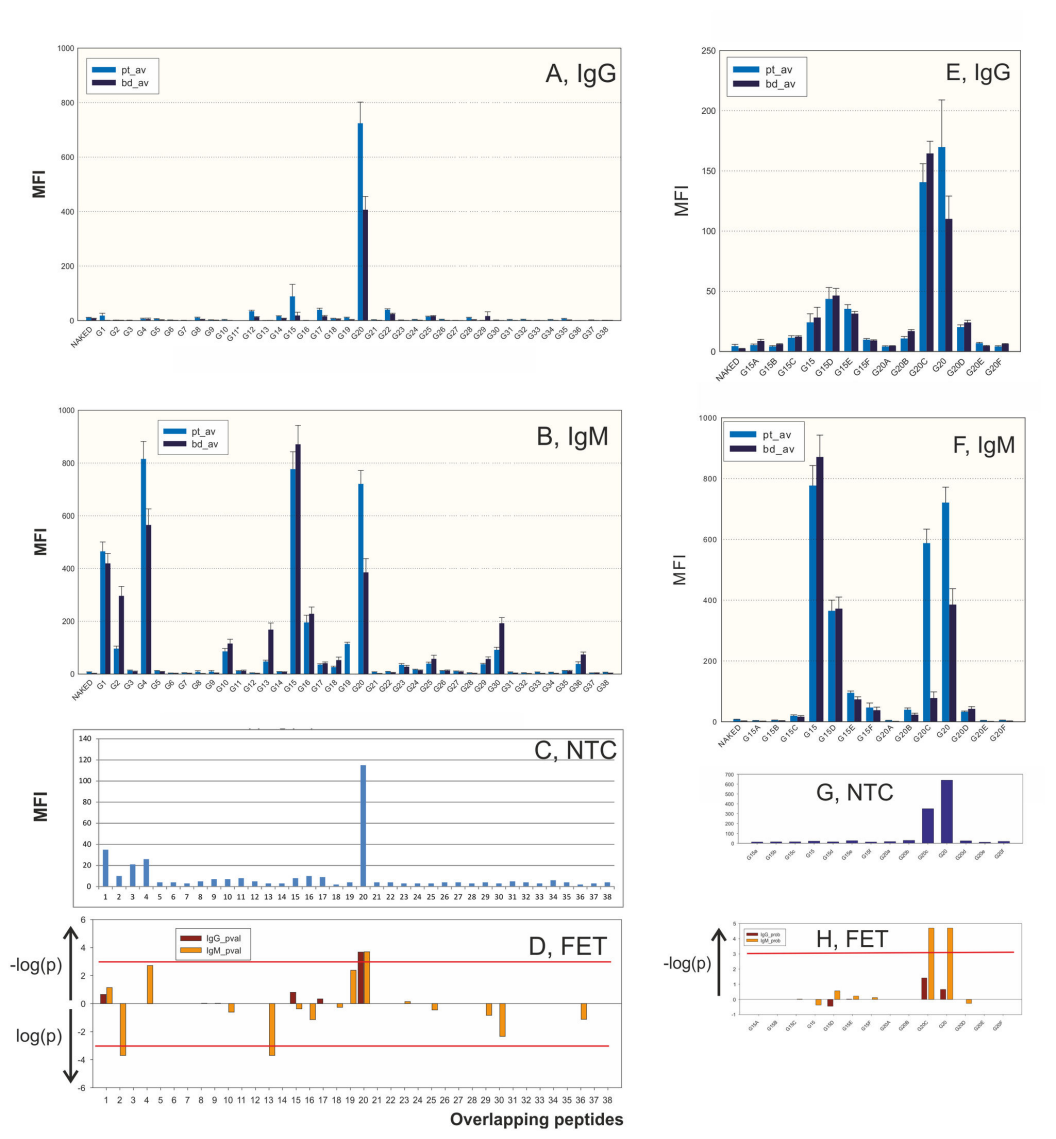
Comparison of IgG and IgM results. A-D: Epitope scanning with synthetic 30-mer peptides overlapping by 15 amino acids, from human HSP60. Peptides were covalently coupled to carboxylated color-coded beads. Binding values were partially subtracted with the non-template control (NTC) value. A. Averages of results with anti-IgG conjugate (protein G). B. Results with IgM (polyclonal anti-human IgM) conjugate. C. Binding of the detection components directly to beads, without presence of patient sample, the NTC. D. 10-logarithm of the p value from the Fischer Exact Test (FET). The upper arrow shows the p value of peptides with ME preference, the lower one shows the p value of peptides with BD preference. The red line denotes a p value of 0.001. Error bars denote standard error of the mean. The dark blue bars (pt_avg) denote averages from ME patient samples, the light blue bars (bd_avg) from BD. The abscissa shows results with successive overlapping peptides (G1-G38). E-H: Further epitope scanning of human HSP60. Peptide sequences were displaced three times, three amino acids at a time, either towards the amino end of human HSP60 (peptides a-c), or towards the carboxyl end (peptides d-f), starting either from peptide G15, or peptide G20. Abbreviations and conventions are as in A--D.

* Footnote: Blood samples were tested against bead-coupled recombinant HSP60 and synthetic peptides derived from HSP60. A cutoff (co) of 200 MFI was chosen for the Fisher Exact Test , based on our previous experience of SMIA, except for the highly reactive recombinant hsp60 antigens, where a cutoff of 4000 MFI was chosen. The most statistically significant results for each antigen category are shown. Further results are shown as needle plots in Figure 4, and Figures SF5, SF6 and SF8 in file S1, and as histograms in figures 1, 4 and SF7 in file S1.

We then identified human HSP60 autoepitopes recognized by patient and blood donor samples. The identification of a preference of patient sample IgM for both human and *E. coli* HSP60 led us to synthesize overlapping human HSP60 peptides. A complete set of 30-mer peptides overlapping by 15 amino acids were coupled to color coded beads and analyzed according to the SMIA protocol. A few peptides, primarily peptide G20, and more weakly peptide G15, reacted in the IgG test, with a weak preference for ME patient samples (Figure 1 A). In contrast, peptides G1, G2, G4, G10, G13, G15, G16, G19, G20 and G30 reacted in the IgM test (Figure 1B). Peptides G1, G4, G15 and G20 reacted strongest. Of those, ME patient samples reacted preferentially with G4 and G20. Peptides G2 and G13 reacted preferentially with BD samples, showing that the system could detect both types of preference. G20 overlaps the apical HSP60 I helix [44], a primary protein and peptide binding site of the HSP60 chaperonin (Figure 1C-F). Nine peptides contained IgM autoepitopes while only two reacted strongly in the IgG test. IgM antibodies were most discriminatory (Figure 1 D). Median Fluorescent Intensity (MFI) values obtained from the NTC and the naked bead were subtracted from the MFI values obtained from ME and non-ME patient and BD samples, as described in Materials and Methods. Essentially the same results were obtained when monoclonal anti-IgG and anti-IgM were used as secondary antibodies in the IgG and IgM tests (see section 3 of file S1). 

We then performed a fine tuning of human HSP60 autoepitope definition. Peptides G15 and G20 reacted more frequently and strongly than the other human HSP60 peptides in the IgG and IgM tests. To better define the two epitopes, a new set of 12 overlapping peptides, where six overlapped the G15 sequence, and six the G20 sequence, were synthesized. When these were tested, the original G15, and the G15d (shifted three amino acids towards the carboxy terminus), peptides gave the strongest reactions, while G20c, shifted three amino acids towards the amino terminus, reacted strongest (Figure 1 E-F).

IgG bound most to the G15, G20c and G20 peptides. None of them gave significant differences between ME patients and controls. The G15 and G20 peptides bound IgM strongly. G15 and overlapping peptides gave no difference between patient and controls. Peptides G20 and G20c had a preference for patient IgM (Figure 1 H and Table 1). 

Certain HSP60 peptides displayed a marked nonspecific protein binding. Peptides G20 and G20c gave a higher NTC value than the other peptides (Figure 1 C and G). Peptide G20c contains the entire primary protein-binding helix (I) of the HSP60. It is therefore possible that the high NTC values were caused by a chaperonin-like activity of the peptide. The NTC values were approximately equal regardless of whether the secondary biotinylated protein was protein G, monoclonal anti-IgG, polyclonal anti-IgM or monoclonal anti-IgM (Figure SF1 in File S1). Thus, the binding of detection components to beads containing human HSP60 peptides G20 and G20c was of a general nature, reminiscent of the broad protein affinity of HSP60. To obtain a measure of the chaperonin-like activity we synthesized five dodecamer HSP60-binding peptides, so-called “strongly binding peptides” [45] in biotinylated form. Indeed, two of five strongly binding peptides bound to human and *E. coli* HSP60, but also to the apical helix I containing G20c peptide (and also to its *Plasmodium* and *Chlamydia* homologs, data not shown; Figures SF1 and SF2; and Table ST6, in file S1). The whole recombinant proteins and the peptides thus had the same specificity. This suggests that the high NTC values seen with the majority of G20c homolog peptides were due to a chaperonin-like protein and peptide binding activity.

**Figure 2:**
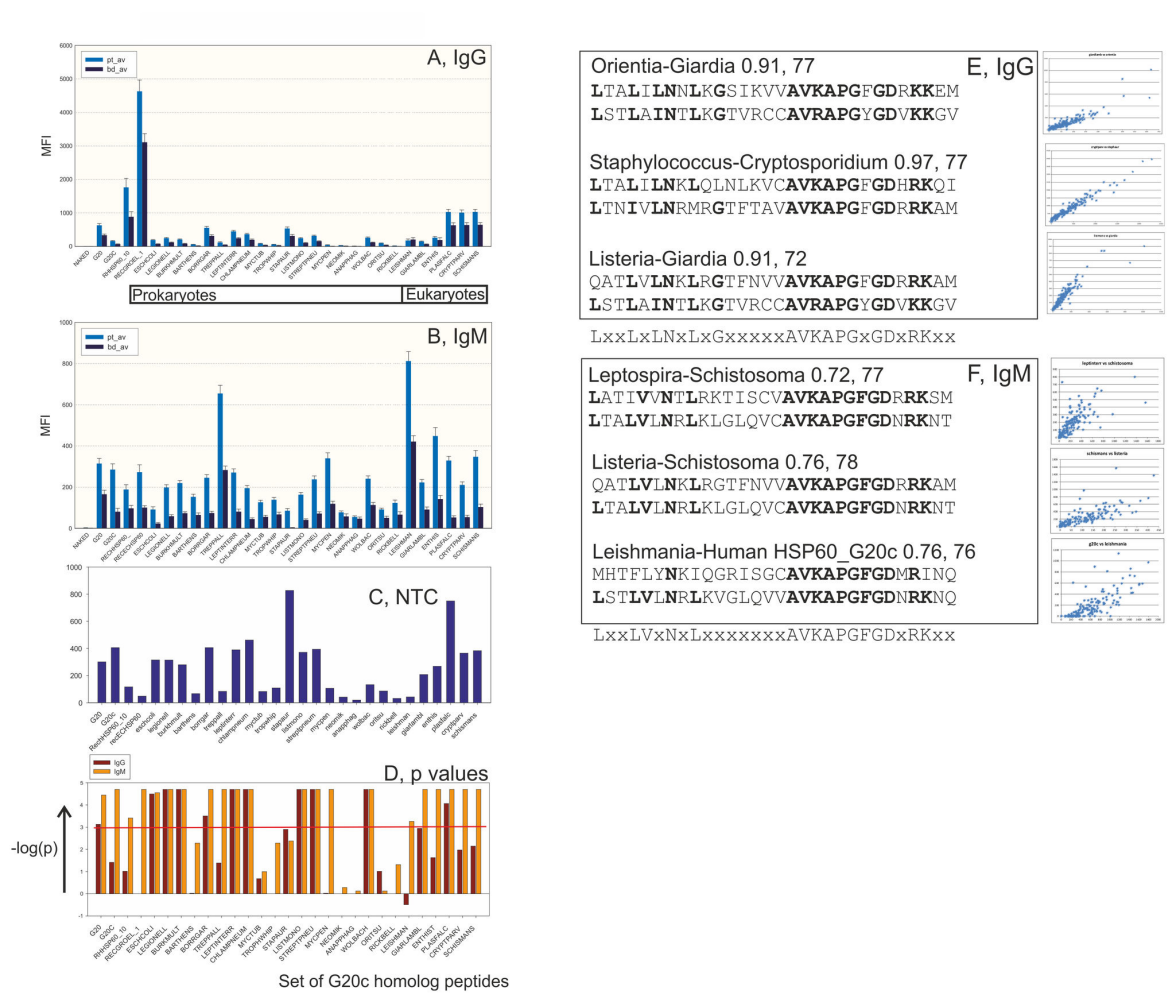
Phyloscan with G20c homologs of 25 bacterial and protozoal HSP60s for IgG (A) and IgM (B) binding. The average binding of patient (pt-av) and BD (bd-av) samples is shown. The relatively high NTC values (C) were partially subtracted as described in Materials and Methods. A significance test (D) using the Fisher exact test is also shown. The red line depicts a p value of 0.001. The peptides G20 (lane 1), G20c (2), recombinant human HSP60 (3) and *E. coli* GroEL (4) were also included. The names of the G20c homolog peptides are explained in File S1. Lanes 24-29 refer to eukaryotes. The rest are from prokaryotes. The order is the same as in the alignment of HSP60 sequences (Figure SF4 in file S1). Ordinates of sections A-C show MFI. E-F: Phylogenetic epitope definition. For each peptide pair, the correlation coefficient, the degree of sequence similarity (calculated with the BLOSUM62 scoring matrix), and the two peptide sequences, is shown. Identical peptide sequences get around 150 points with this scoring system. Three discordant sequence pairs with high IgG (E) and IgM (F) serological correlation are shown.

When the G20c peptide had been identified we were led to perform phylogenetic scanning of HSP60 G20c homologs for ME and BD antibody binding. Amino acid sequences of HSP60 from bacteria, protozoa, fungi and humans were aligned (Figure SF4 in file S1). A set of 25 G20c homolog 30-mer peptides (Table ST3 in file S1) were synthesized and tested in the IgG and IgM tests. The highest IgG values were with the *Schistosoma mansoni*, *Plasmodium falciparum*, *Cryptosporidium, Staphylococcus*, *Borrelia*, *Leishmania, Chlamydia* and *Leptospira* homologs. In the IgM test, especially strong reactions were seen with *Leishmania*, *Treponema*, *Entamoeba* and *Chlamydia* homologs (Figure 2). The five G20c homolog peptides with the highest positive (100%) and negative predictive values (62-68%) for ME in the IgM test were from *Staphylococcus*, *Plasmodium, Listeria*, *Burkholderia* and *Chlamydia* (Figure SF5 in file S1) 

The large variation of binding to the G20c homologs created an opportunity to study the degree of antigenic cross-reactivity of the peptides. When results from all 152 samples were correlated with each other, some of the MFI with G20c homologs were highly correlated (Table ST7 in file S1). The highest were with the prokaryote-prokaryote combinations which had a similar peptide sequence (BLOSUM score over 100). However some prokaryote-eukaryote combinations, for example between *Staphylococcus* and *Homo*, *Chlamydia* and *Homo* and *Leptospira* and *Plasmodium*, which have a considerable sequence difference (BLOSUM62 score=<100), also gave highly correlated antibody binding. IgG measurements tended to be more highly correlated than IgM measurements. Discordant sequence combinations with high IgG correlation coefficients, indicating a microbe-human epitope mimicry, more often involved the human G20c peptide (shown as “*Homo*” in Table ST7 in file S1) than those of IgM. Five of twenty high IgG correlations involved the human homolog, while only one of twenty high IgM correlations did so. 

Likewise, some IgM MFI with G20c homologs were highly correlated but generally gave lower correlation coefficients than IgG MFI. Discordant sequence combinations which gave high correlations were for example *Treponema* versus *Schistosoma* and *Leishmania* versus human G20c (Table ST7 in file S1). 

The high antibody reactivity correlations of discordant sequence pairs allowed us to define a consensus sequence of serologically important amino acids in the 30-amino acid G20c homologs: LxxLxxNxLxxxxxxxAVKAPGFGDxRKxx. This consensus was very similar for IgG and IgM correlations of discordant peptide pairs. It is also similar to the consensus from the alignment of G20c homologs in Figure SF4 in file S1. We tested ME, the seven non-ME patient samples, and BD samples separately, but all three gave the same consensus sequence. From the evaluation of highly serologically correlated but sequencewise discordant G20c homologs, it seems that the sera from the three groups recognized G20c peptides in a qualitatively similar way. 

The results from human HSP60 and phyloscanning with G20C homologs led us to perform epitope scanning with overlapping bacterial HSP60 peptides. Although controversial, previous publications have raised the possibility of *Chlamydia* and *Mycoplasma* as contributing factors in the etiology of ME (see the detailed account in section 7 of file S1). We therefore continued our study by synthesizing overlapping 30-mers from *Chlamydia pneumoniae* and *Mycoplasma penetrans* HSP60. HSP60 peptides from the related Mycoplasma pneumoniae and *Chlamydia trachomatis* should be included in future extensions of the study.

**Figure 3:**
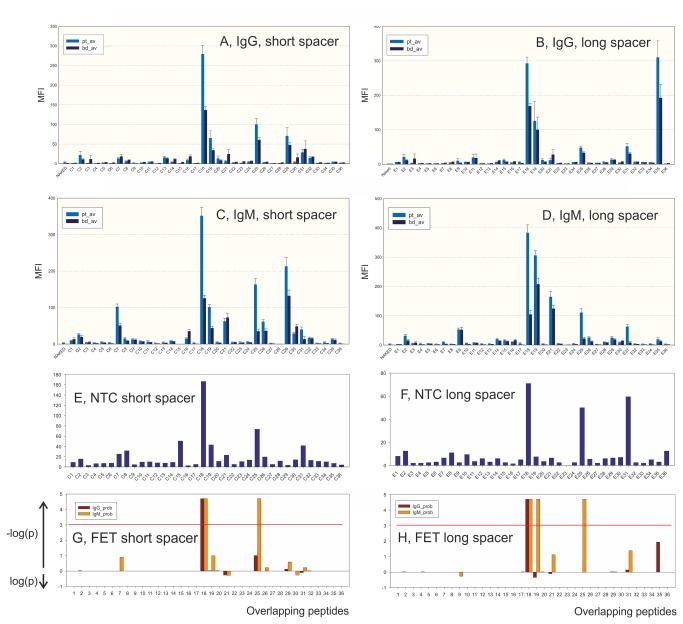
Epitope scanning with overlapping *Chlamydia pneumoniae* HSP60 peptides coupled with short (tri-ethylene glycol; C series) and long (hexa-ethylene glycol; E series) spacer. Reactions (MFI, after subtraction of NTC values) in the IgG and IgM assays, were measured. Frames E and F show the NTC values. Frames G and H show the probabilities calculated according to the Fisher Exact Test. The red line shows an estimated probability of 0.001.

The overlapping peptides from HSP60 of *Chlamydia pneumoniae* (short and long spacer; Figure 3) were tested against samples from patients and BD. Samples from the Training group with ME diagnosis (n=69) were treated as one group which in Figure 3 is named “patients”. 

The IgG reactions for peptides with short (C series) and long (E series) spacer were similar (Figure 3). E19 gave higher values than C19. C25 and C29 gave higher values than E25 and E29, respectively. A conspicuous difference was that E35 gave much higher values than C35. NTC values peaked in peptides C18 and C25, and E18, E25 and E31. 

One peptide sequence, embodied in the C18 (Fisher Exact Test, FET, p<0.001) and E18 (FET p<0.001) peptides, gave a significant IgG preference for ME patient samples. The IgM reactions gave similar results for peptides with long and short spacer. E19 gave higher values than C19. C25 and C29 gave a stronger reaction than their long spacer counterparts. E31 gave higher values than C31. Although the E35 peptide gave a prominent reaction with IgG, it gave hardly any reaction in the IgM test. The highest selectivity for patient samples (FET p<0.001) was seen with C18 and C25, and E18, E25 and E31, in the short and long spacer systems, respectively. All significant differences had a preference for ME patient samples. The results are detailed in needle plots in Figure SF6 in file S1.

The results of scanning for IgG and IgM epitopes with overlapping *Mycoplasma penetrans* HSP60 peptides are shown in Figure SF7, and as needle plots of the most discriminatory peptides in Figure SF8, both in file S1. The difference between peptide D18 and the overlapping *Mycoplasma* G20c homolog is only a two amino acids shift, yet the G20c homolog, and not D18, reacted with ME preference. It is reminiscent of the large difference in antigenicity between G20b and G20c, which differ by a three amino acid frame shift (Table ST2 in file S1). NTC values higher than for the rest of the *Mycoplasma* peptides were observed with three peptides, the G20c homolog, D9 and D25. D9 from the amino and D25 from the carboxy portion of *Mycoplasma* HSP60 reacted preferentially with ME samples, primarily in the IgG test. 

A small collection of sera with high and low amounts of antibody to *Chlamydia pneumoniae* were tested against whole bacterium *Chlamydia pneumoniae* antigen attached to color coded beads. We found a concordance between microimmunofluorescence (MIF) and ELISA positivity with whole bacterium but did not see a significant correlation between whole bacterium MFIs and anti-HSP60 overlapping peptide MFI in SMIA (data either not shown, or shown in Figure SF10 in file S1). This result together with the high HSP60 conservation and the cross-correlations between G20c homolog peptides, indicates that a significant portion of the anti-HSP60 response measured by the peptides is due to cross-reactivity. Its origin from infection with a specific microbe is hard to deduce from our data.

**Figure 4:**
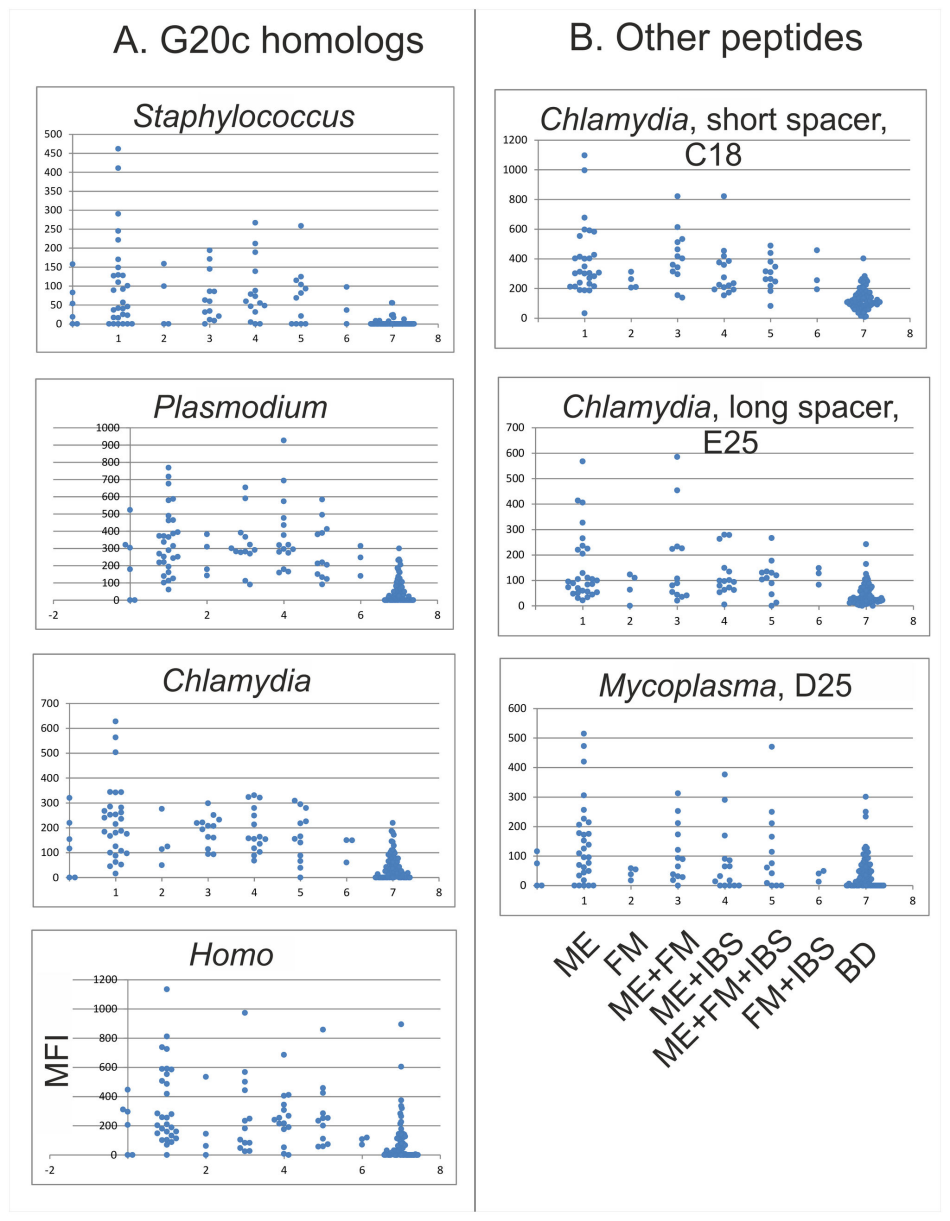
Needle plots of the most ME selective HSP60 peptides found in the Training set. All were from IgM assays. A. G20c homologs. The human HSP60 homolog is shown for comparison. B. Other HSP60 peptides. The *Chlamydia* E25 and *Mycoplasma* D25 peptides came from the carboxyl terminal half of HSP60, while the Chlamydia C18 came from its center, overlapping G20c and the apical helix I. ME patient samples are in lane 1, FM in lane 2, FM+ME in lane 3, ME+IBS in lane 4, ME+FM+IBS in lane 5, FM+IBS in lane 6 and BD in lane 7. The dots in lane 0 are from control samples (NTC, IgG, ME pool, etc). Ordinates: MFI.

An overview of results with the peptides which were most selective for ME versus BD samples is shown in Figure 4. The most discriminatory were *Staphylococcus*, *Chlamydia* and *Plasmodium* G20c homologs in the IgM test. But peptides C18 and E25 from *Chlamydia pneumoniae*, and D25 from *Mycoplasma penetrans* were also discriminatory in the IgM test. The latter two are from the carboxy terminal half of HSP60 and are not related to G20c. 

#### We then retested with a panel of HSP60 antigens

When all previous results were subjected to multivariate (principal component) analysis, certain HSP60 derived antigens showed a higher influence on the variance of the whole data set. The first principal component (explaining 53% of the whole variance, not shown) was most dependent upon the *Entamoeba* and *Schistosoma* G20c homolog peptides, human HSP60 and *E. coli* HSP60 recombinant proteins, plus a number of bacterial HSP60 peptides (G20c homologs and others). From these considerations, and the degree of selectivity for ME defined for each antigen, we composed a panel of 25 HSP60-derived antigens intended for further evaluation with new ME, non-ME patient and BD samples (Table ST8 in file S1). When IgM reactions above the MFI of IgM in a pool of the ME samples were provisionally scored as a “pepscore”, 28 ME patient samples were positive (41%, out of 69) (Table ST9 in file S1). None of the seven non-ME (FM and FM+IBS) and none of the 76 blood donor samples scored. The number of FM and IBS samples was however too small for conclusions whether their reactivity with the panel antigens was greater or smaller than those of ME samples. 

### Evaluation of the epitopes recognized by previously not tested samples

**Figure 5:**
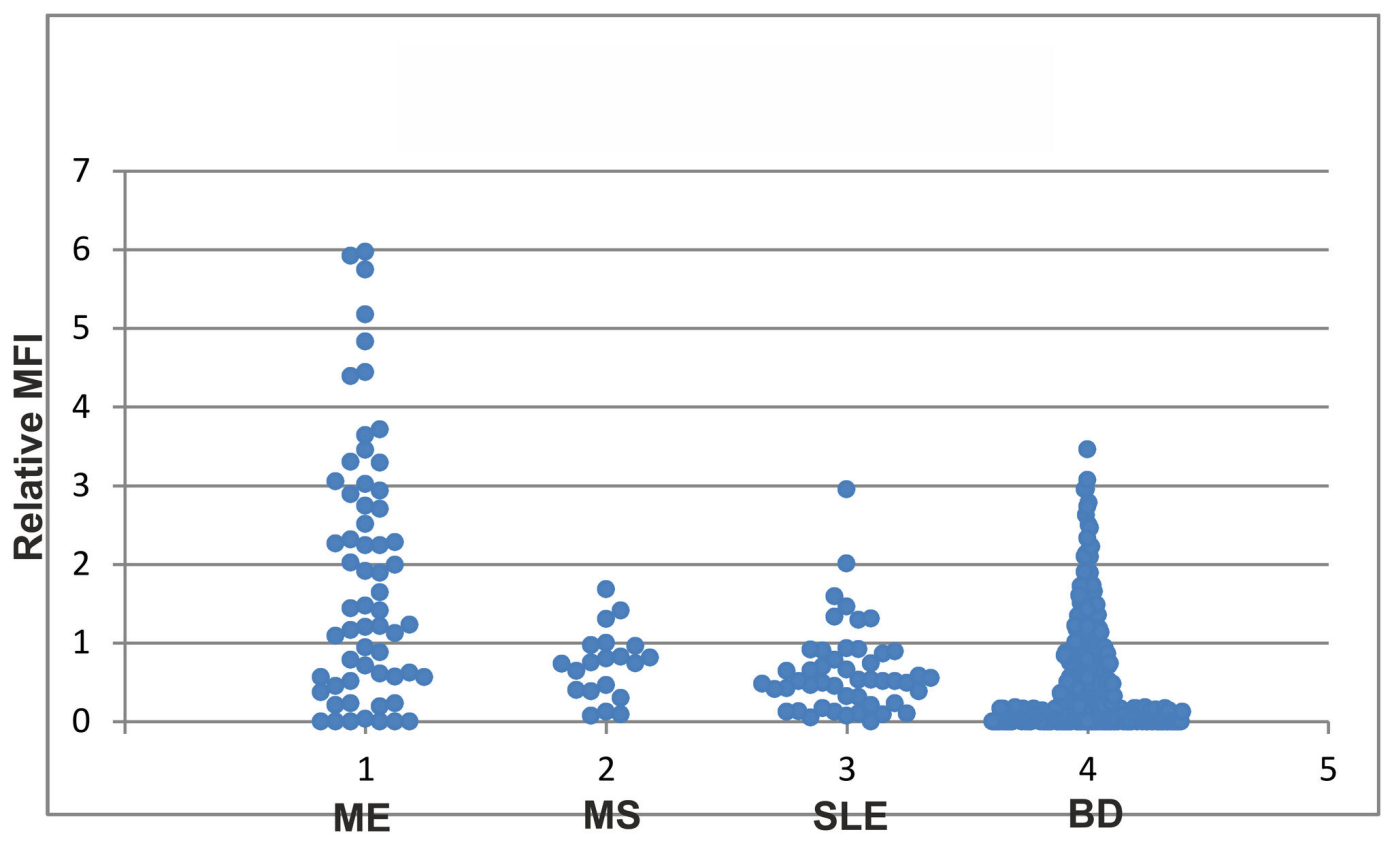
Needle plot of the relative MFI of the *Chlamydia peumoniae* G20c homolog (peptide 12) and the ME, MS, SLE and BD samples in the Evaluation set. Relative MFI were calculated by division of the MFI for each sample with the MFI of a pool of 69 ME samples, diluted 1/10.

The biomarker candidate antigen panel (Table ST8 in file S1) was applied to the Evaluation set (61 samples from not previously tested ME patients, 20 MS plasma samples, 48 SLE sera and 323 BD sera). Binding was expressed relative to that of an ME pool which was tested in the same round. Most antigens retained a preference for ME relative to BD, as in the training set. However, BD samples from 2005 and 2013 had a higher proportion of reactions with the majority of the antigens, making the preference weaker (Figure SF9 in file S1). The majority of the candidate biomarkers of the antigen panel did not hold up with the Evaluation set, illustrating the danger of relying on just one data set for definition of biomarkers. It was only peptide 12 (P12, the G20c homolog of *Chlamydia pneumoniae*) which retained a marked ME preference. P12 IgM reactivity did not correlate with IgM to whole *Chlamydia pneumoniae* antigen (Figure SF10 B in file S1). The MS sera bound weakly or not at all, although the recombinant *E coli* HSP60 reacted preferentially with MS samples (not shown). Many of the panel antigens gave relatively strong signals with the SLE sera. Figure 5 and the ROC curve in Figure SF11 in file S1 show that at a cutoff of 3.1 relative to the MFI of the ME pool, 15 (24%) of the 61 ME samples reacted, while 1 (0.003%) of the 399 non-ME samples reacted. At a relative MFI cutoff of 2, 25 (41%) of 61 ME and 17 (4.2%) of the 399 non-ME (13 of 302 BD) were detected, a similar result as with the Training set. Both ME vs non-ME differences are highly statistically significant. We observed differences in frequency of reactivity between the BD subsets obtained in different years and different seasons (Figure SF9 in file S1). This indicates that there is a seasonal variation of IgM reactions, unrelated to ME, for several of the antigens. It is to be expected that the frequency of infection-induced, partially cross-reactive antibodies, varies according to the epidemiological situation. 

## Discussion

HSP60 is a key molecule for a number of functions, for instance as a mitochondrial chaperonin participating in cellular stress reactions and in presentation of molecules both at cell surfaces and extracellularly. It may also regulate NK cell activity [46]. Human HSP60 probably originates from HSP60 of the endosymbiotic alphaproteobacteria which gave rise to mitochondria [47]. Its high conservation in eu- and prokaryotes [1] and high antigenicity predispose it for immunological cross-reactivity [2,48]. Single nucleotide polymorphisms in human HSP60 cause hereditary spastic paraplegia [49]. HSP60 malfunction thus theoretically may cause as diverse effects as mitochondrial dysfunction, immune dysregulation and central nervous system ailment. It is a central molecule in autoimmunity [50]. Autoimmune diseases where HSP60 antibodies have been reported are MS, T1D [3–6] and atherosclerosis (see sections 6-8 of file S1, and below). This paper identifies cross-reactive HSP60 epitopes with a large set of long synthetic peptides, and their selective recognition in some patients suffering from ME. A future logical extension could be to investigate if affinity purified antibodies to those epitopes interfere with HSP60 or mitochondrial function.

Overlapping peptides from human HSP60 identified a frequently recognized autoepitope (peptides G20 and G20c) of human HSP60, which overlaps the apical I helix, a major protein binding site of the HSP60 chaperonin. Epitopes detected in the IgM test were more numerous than those detected in the IgG test. This was observed in the three epitope scans, with human, *Chlamydia* and *Mycoplasma* HSP60. This clear difference in epitope repertoire between the IgG and IgM tests has not, to our knowledge, been observed before. The Ig class switch may involve an epitope selection based on degree of autoreactivity and avidity. The mode of presentation of peptides at the bead surface was also important. Differences were seen between reactivity patterns of *Chlamydia* HSP60 peptides with different spacer lengths, most pronounced in the IgG test.   

A few peptides from human HSP60 retained a chaperonin-like rather promiscuous protein binding activity. This non-antibody dependent binding created an uncertainty of how much of the signal from the antibody dependent binding that was specific. However, in experiments with blocking proteins and titrations we found that the protein content of the patient sample was enough to block the general protein binding of some HSP60 peptides (Figure SF3 in file S1). The dilution fluid of the assay system was protein-free. This allowed us to detect and evaluate the chaperonin-like peptide and protein binding of certain HSP60 peptides. We used five biotinylated dodecapeptides selected for high HSP60 affinity [45]. Two of them bound to both whole recombinant human and *E. coli* HSP60 and to G20c homolog peptides containing helix I in our SMIA test. Thus, the binding specificity of the G20c peptides was similar to that of the whole HSP60 protein. The protein binding activity preferentially occurred close to the I helix in the apical domain, a primary protein binding structure of the HSP60 chaperonin [45]. 

A few human HSP60 peptides reacted selectively with ME samples compared to those from BD. IgM reactions were more discriminatory than IgG reactions. A human HSP60 peptide (G20c), shifted three amino acids towards the amino end relative to the G20 peptide, had a higher ratio of antibody binding to NTC binding with human samples than the G20 peptide. It was therefore chosen as the basis for further investigations.

In an extension of the epitope scanning we compared peptides homologous with the human G20c peptide, a “phyloscan”. Certain ME and BD samples gave high IgG and IgM antibody reactions to HSP60 peptides from *Leishmania* and *Schistosoma*. Like *Plasmodium*, these microbes are rare in Sweden. The highly conserved sequence of HSP60 can lead to unexpected serological cross-reactions. The high correlation coefficients of some structurally discordant peptide pairs support this conclusion (Tables ST7A and ST7B in File S1, and Figure 3). Despite a clear sequence difference and a large phylogenetic distance peptides from *E. coli*, *Listeria*, *Burkholderia*, *Staphylococcus* and *Cryptosporidium* displayed an almost perfect antigenicity correlation in IgG data. A less perfect, but still high, correlation was seen between antigenicities of many G20c homologs in the IgM test. Thus, the diversity of sequence recognition was greater in the IgM than in the IgG tests. One interpretation is that a greater diversity of IgM recognition is replaced by a simpler IgG recognition during maturation of humoral immunity, suggesting an anti-HSP60 “final common pathway". The same conclusion can be drawn from the higher number of epitopes recognized in the IgM test with overlapping peptides from human HSP60, *Chlamydia pneumoniae* HSP60 and *Mycoplasma penetrans* HSP60. A large initial number of IgM epitopes may be replaced by a few more crossreactive IgG ones. It is reasonable to assume that repeated exposures to microbial HSP60 epitopes normally is channeled into harmless, more or less autoreactive, antibodies. A successive “channeling” of HSP60 epitopes to less harmful ones has been observed during the course of adjuvant arthritis in rats [51,52]. The phenomenon of “epitope spreading” in autoimmunity [53–55] may be a similar phenomenon. The profound conservation and cross-reactivity of certain HSP60 epitopes is a critical task for self/nonself discrimination, with a great potential for mistakes. 

The observations from the phyloscan, and previous publications, led us to synthesize three more overlapping peptide sets, one with a tri-ethylene glycol spacer from *Mycoplasma penetrans* HSP60, and two from *Chlamydia pneumoniae* HSP60. The latter two either had a tri- or a hexa-ethylene glycol spacer [56]. The spacers yielded similar, but not identical results (commented on in the section 9 of File S1).The relatively long peptides (30-mers) employed here may allow some secondary structure and mimicking of conformational epitopes. It is however far from the more complete epitope representation of an entire protein. 

Several of the discriminatory peptides defined using the Training set came from *Chlamydia pneumoniae* and *Mycoplasma penetrans*. Three major HSP60 regions contained ME-selective epitopes, halfway from the amino terminus to the middle, the middle, and halfway from the middle towards the carboxy terminus. Thus, the apical helix I domain is not the only ME-specific domain. IgM epitopes were more frequently discriminatory between ME and non-ME samples. Many, but not all, of the discriminatory peptides also gave higher NTC values. The blocking effect of the proteins in the samples, and the partial subtraction procedure, both of which treated ME and non-ME samples alike, controlled for the chaperonin-like binding to those peptides. 

Most of the antigens defined based on the Training set were not as ME-selective in the Evaluation set. As shown in Figure SF9 in file S1, this was due to the varying IgM reactivity among blood donor samples from different years. Thus, it is likely that many of the “ME specific” IgM reactions found in the Test set were due to a low reactivity in the 2010 blood donor samples. However, the *Chlamydia pneumoniae* G20c homolog peptide in the IgM test retained a marked ME preference. It merits consideration as member of a future ME biomarker panel.

**Figure 6:**
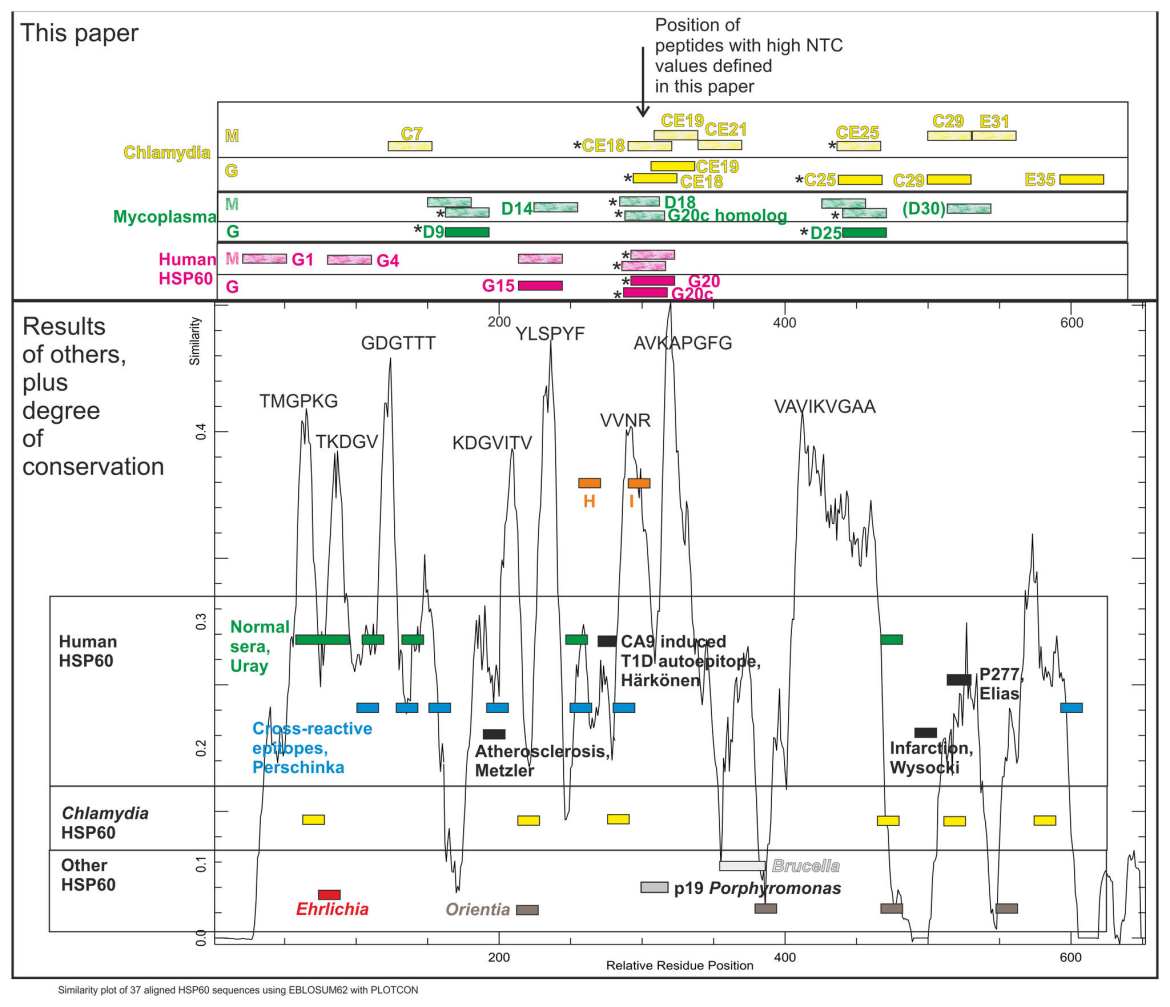
Overview of antigenicities of human, chlamydia and mycoplasma HSP60 peptides from our study, in the context of results from other HSP60 epitope mapping studies. A similarity plot of 34 aligned HSP60 amino acid sequences (Fig. SF4 in file S1) is also included, scoring similarity with the EBLOSUM62 matrix and a sliding window of 10 amino acids, using the program Plotcon (at the EMBOSS web site). Peaks of conservation are labeled with the human HSP60 sequence variant. The X axis shows the position in the alignment. IgG and IgM binding peptides defined in the present paper are shown. Peptides which discriminated ME from non-ME patients and BD (in the Test set) are marked with asterisks. *Mycoplasma* HSP60 peptides are green, *Chlamydia* HSP60 peptides are yellow, human HSP60 peptide magenta. Results of others include epitopes recognized by normal human sera (Uray), crossreactive epitopes of human, *Chlamydia*, *E. coli* and *Mycobacterium* HSP60/65 recognized by sera of individuals with atherosclerosis (Perschinka), human HSP60 epitopes (black) selectively recognized in patients with coronary infarction (Wysocki), atherosclerosis (Metzler) and after Coxsackie A9 infection (Härkönen). P277 (Elias) is a human HSP60 peptide containing T and B cell epitopes. It was used in vaccination experiments with NOD mice. Previously reported chlamydia HSP60 epitopes are shown in yellow. A protective and diagnostically useful *Ehrlichia* peptide (red) and diagnostically useful *Orientia tsutsugamushi* St58 (dark gray) and *Brucella* HSP60 peptides (light gray) are also shown. Literature references are given below, and are further discussed in section 6 of File S1. Many of these peptide sequences are also shown in detail in Tables ST1-ST5, and in the alignment of Figure SF4 in file S1.

As shown in Figure 6, reactivity to a variety of HSP60 epitopes is common in both healthy individuals and persons with a wide variety of diseases. A broader study including more samples from different kinds of SLE patients and other autoimmune diseases like T1D and rheumatoid arthritis plus patients suffering from myocardial infarction and other signs of atherosclerosis, should be conducted. 

### Our results compared to previous work on HSP60 epitopes

When our epitope scanning results were placed in the context of previously reported HSP60 epitopes, a complex picture emerged [6,57–76]. It is summarized in Figure 6 and section 6 in file S1). Various disease correlations and diagnostic applications of short synthetic HSP60 peptides have been reported. Our results with 30-mer overlapping peptides of human, *Chlamydia* and *Mycoplasma* peptides were reminiscent of, but far from identical to, previous results. 

### Implications of the results for the understanding of ME pathogenesis

HSP60 is a central autoantigen. The function of HSP60 is to interact with proteins in a rather promiscuous way, so it is no surprise that it appears as a candidate in many pathomechanistic surveys. Although we find relatively high levels of antibodies to specific HSP60 epitopes in ME, the high frequency of anti-HSP60 antibodies in control samples, and the high conservation and many functions of HSP60, argue against simplistic conclusions regarding its etiological role(s) and possible therapeutic implications [77]. Results of others indicate that its cross-reactions also involve epitopes of other chaperonins [78], and maybe viral capsid proteins [63]. Our results are compatible with the presence of infection-elicited autoantibodies in ME. The finding that autoantibodies occur in ME is not new [9–14]. An increased frequency of antibodies to cardiolipin has been reported [12]. Like HSP60 it is a molecule which occurs in both mitochondria and bacteria. The panorama of autoantibodies in ME should be further explored. 

### Speculation regarding a possible pathogenic role of anti-HSP60

The profound fatigability in ME may be related to impaired aerobic energy metabolism during recovery from exercise [79,80] and mitochondrial dysfunction [15,81–83]. One can speculate that anti-HSP60 antibodies in ME also interfere with HSP60 function, in mitochondria and elsewhere. In fact, autoantibodies to mitochondrial tricarboxylic acid enzymes occur in primary biliary cirrhosis [84]. Immunological abnormalities, like NK cell dysfunction, are frequently reported in ME [85,86]. It is conceivable that a disturbance in HSP60 turnover brought about by anti-HSP60 could influence NK cell activity. The leader of HSP60 contains a nonapeptide (QMRPVSRVL) which can bind to HLA-E and diminish HLA-E mediated inhibition of NK cell activity, leading to increased NK cell killing of stressed cells [46]. An autoantibody dependent mechanism behind ME is also in accordance with the results of anti-CD20 monoclonal antibody treatment, of Fluge and Mella [87,88], where B cell depletion led to substantial improvement in around 60% of ME patients. It should be investigated if ME has a similar pathogenesis as the postinfectious neuroimmunological diseases (briefly referenced in section 8 of file S1).

## Conclusions

Using three sets of completely overlapping synthetic 30mer peptides in a multiplex suspension array, we defined the pattern of IgG and IgM recognition of HSP60 epitopes of blood donors and ME patients. Certain peptides of HSP60 of pro- and eukaryotes (including humans), primarily from its apical I helix, retained a chaperonin-like protein binding activity but also bound IgG and IgM from ME samples more frequently and more intensely than those from the controls. IgM epitopes were more numerous than IgG epitopes. A few of them were discriminatory for ME. The concordance of results between HSP60 peptides from diverse hosts advocates a crossreactive source (via mimotopes) of the autoantibodies which occur in ME and controls. A consensus peptide sequence from a major crossreactive epitope coinciding with the apical I helix was defined. Whether the anti-HSP60 antibodies of ME patients and controls arise from one or several acute or chronic infections cannot be determined from this purely serological study. We identified a synthetic HSP60 peptide which potentially can be diagnostically useful, together with other markers, in a future biomarker panel for ME patients. Regardless of the possible relation of anti-HSP60 antibodies to ME, the definition of HSP60 epitopes and their crossreactivities are important outcomes of this work.

## Methods

### Clinical samples

Samples were provided by the co-authors CGG and OZ, Gottfries Clinic AB, Mölndal, Sweden. The Training set (n=76) included 69 ME patients, of which 29 patients had only ME, 13 ME+FM, 15 ME+IBS and 12 ME+FM+IBS. The seven non-ME patients consisted of 4 patients with FM and 3 with FM+IBS. The Evaluation set included 61 patients with the main diagnosis of ME, but 5 patients did also fulfill the criteria for FM and 6 for IBS. 

The ME diagnosis was made according to the Canadian criteria [89], the FM diagnosis according to the ACR classification [90] and the IBS diagnosis according to the Rome II criteria [91]. ME samples in the Training set was originally collected for a study of the XMRV virus. ME samples in the Evaluation set were originally collected for a study of the psychotropic drug OSU6162 [92]. As healthy controls we used 76 blood donors in the Training set, and 323 blood donors in the Evaluation set. Their sera came from the Uppsala Academic Hospital blood bank. They were analyzed according to a general permission to test for blood-borne microbes obtained at blood donation. The controls were not age and sex-matched to the patients. MS (n=20) and SLE (n=50) samples were obtained from the department of Neurology and Internal Medicine of the Uppsala University Hospital. 

#### Ethics statement

In summary, all samples were tested anonymously. ME samples were collected according to the ethical permissions Dnr 680-09, 960-12 (Training set) and Dnr 806-11, 029-13 (Evaluation set) granted by the ethical commission of the University of Gothenburg. The blood donor control samples were used according to a general permission granted by the Uppsala University blood bank to test for blood-borne microbes. It includes written consent from the blood donor. MS and SLE samples were tested according to ethical permissions Ups-01-81 and Ups-01-380, granted by the Uppsala University Ethical committee. The *Chlamydia* antibody positive sera were tested anonymously, according to patient consent. 

### Other human samples, antigens and synthetic peptides

Positive and negative sera with a known content of microbe-specific antibody were also included. Fourteen positive *Chlamydia pneumoniae* sera were obtained with the accredited methods at the department of clinical microbiology, Uppsala Academic Hospital. In experiments not shown, we coupled the lysate from *Chlamydia pneumoniae* elementary bodies (kindly provided by dr Eva Moreno, Vircell, Spain) to color-coded beads and tested whether it reacted selectively with patient samples compared to blood donor (BD) samples in IgG or IgM tests. There was no such selectivity. Other sera not related to the study patients, of known *Chlamydia pneumoniae* antibody status (in microimmunofluorescence or commercial ELISA), were run against the same antigen. A clear correlation was seen, showing that the bead-based *Chlamydia pneumoniae* elementary body serology test had the capacity to detect *Chlamydia pneumoniae* antibodies. These results indicate that the immune response to the whole bacterium is different from the response to the G20c epitope on HSP60. The high cross-reactivity of the latter means that the response to it is influenced by exposure to other HSP60-containing microbes.

Other controls were a standard preparation of human gammaglobulin (5 mg/ml; referred to as “IgG”; IgTrol, Svanova Biotech AB, Uppsala, Sweden, cf. [93], diluted 1:10. It was included in the IgG runs. A pool of all 76 ME plasma samples were included in both IgG and IgM runs at two different dilutions (1:5 and 1:10). Both were used to monitor the repeatability of the procedure. 

The synthetic peptides are further described in section 2, and Tables ST2, ST3 and ST5 of File S1. 

*Chlamydia pneumoniae* elementary body and *Mycoplasma pneumoniae* whole cell antigens were each coupled to a bead. The two native antigens were kindly provided by dr Eva Moreno, department of antigen production, VIRCELL, S.L. Spain. 

### Preparation of beads for use in SMIA, the SMIA procedure, validation with protein A absorption and alternative second antibodies, and procedures for data management and statistics

This is detailed in section 3 of File S1.

## Supporting Information

File S1 **Supporting information, tables (ST1-ST8), and figures (SF1-SF11).**
(DOCX)
