# Supplementary material for: Epitopes of Microbial and Human Heat Shock Protein 60 and Their Recognition in Myalgic Encephalomyelitis
Source: PLoS One. 2013 Nov 28;8(11):e81155. doi: 10.1371/journal.pone.0081155 (PMC3842916; doi:10.1371/journal.pone.0081155)
Supplement: File S1 — Supporting information, tables (ST1-ST8), and figures (SF1-SF11). (DOCX) [file pone.0081155.s001.docx]

**Supplementary information**

**Results from the epitope definition stage**

**Section 1. Further sample information**

All ME patients were from the Gottfries Clinic in Gothenburg. The Test (n=69; during 2008-2009) and Evaluation (n=61; during 2010-2011) sets were diagnosed according to the same criteria, but the patients were not the same. The main diagnosis of all 61 in the Evaluation set was ME. The Test set included both samples from ME and non-ME patients,where 69 had ME with of without FM and IBS, 4 had FM and 3 had FM+IBS.

**Section 2. Further information regarding antigens for the suspension array**

*Synthetic peptides*

Human HSP60 peptides were chosen from the published sequences of human HSP60 (GenBank id NP_955472), *Chlamydia pneumoniae* HSP60 (Genbank Id NP_224342) and *Mycoplasma penetrans* HSP60, also named GroL (GenBank ID NP_757486). All peptides were 30-mers and had a three-carbon polyethylene glycol spacer coupled at the amino end. The spacer started with a primary amino group. Two overlapping series of *Chlamydia* peptides were tested, one with a three-carbon and one with a six-carbon polyethylene glycol spacer. The peptides overlapped by 15 amino acids. The peptides were synthesized by the coauthor dr Rüdiger Pipkorn. Lyophilized peptides were dissolved in sterile phosphate-buffered saline (PBS), pH 7. The dissolution sometimes had to be facilitated by warming the solution to 37°C overnight on a shaker or by sonication for 5 min. Highly hydrophobic peptides were dissolved in 10 to 50% (vol/vol) dimethyl sulfoxide (DMSO) (Sigma D2650). Initially, 38 overlapping peptides of HSP60 were synthesized, (Supplementary table ST1).

In initial experiments, two human HSP60 peptides G15 and G20, gave significant IgG reactions. To better localize the epitopes, peptides overlapping G15 (G15a, G15b, G15c, G15d, G15e, and G15f) and G20 (G20a, G20b, G20c, G20d, G20e, and G20f), systematically shifted three amino acids before and after G15 and G20, respectively, were synthesized (Supplementary table ST2).

A human HSP60 peptide (G20c) showed a higher antibody binding activity than the human HSP60 G20 peptide. Twentysix new peptides were therefore designed after an alignment of Human HSP60 G20c analogs with HSP60 of many microbes, spanning much of the HSP60 variation. The prokaryotes *Orientia tsutsugamushi, Neoehrlichia mikurensis, Wolbachia, Legionella pneumophila, Borrelia garinii, Bartonella henselae, Tropheryma whipplei, Rickettsia bellii, Anaplasma phagocytophilium, Burkholderia multivorans, Escherichia coli, Chlamydophila pneumoniae , Staphylococcus aureus, Mycobacterium tuberculosis, Mycoplasma penetrans, Listeria monocytogenes, Streptococcus pneumoniae, Treponema pallidum,*and the eukaryotes *Leishmania (strain Friedlin), Schistosoma mansoni , Plasmodium falciparum, Leptospira interrogans, Giardia lamblia, Cryptosporidium parvum,* and *Entamoeba histolytica* were included (cf. Supplementary table ST3).

Further, 36 overlapping peptides from *Mycoplasma* penetrans HSP60 (Supplementary table ST4), and two sets of 36 overlapping *Chlamydia pneumoniae* HSP60 peptides, with a three and six carbon polyethylene glycol spacer, respectively, were synthesized (Supplementary table ST5).

*E. coli* HSP60 (GroEL) selectively binds tryptophan-rich synthetic peptides (here termed “Strongly Binding Peptide, SBP”, defined from panning with *E. coli* HSP60 of a peptide library ([*1*](#_ENREF_1)). The binding of such a peptide (here called SBP1), defined by crystallography, was close to the apical domain helix I. Binding of SBPs is therefore a way of demonstrating chaperonin activity. We synthesized five SBP dodecamers defined in (Supplementary table ST6). They were biotinylated at the amino terminus. The biotin was joined to the SBP via a 6 carbon polyethylene glycol spacer, the same arrangement as in the overlapping long spacer chlamydia HSP60 30-mer peptides.

**Table ST1.** The amino acid sequences of overlapping peptides of intact human HSP60 (GenBank id NP_955472).

Turqoise background: Cross reactive epitopes (between Mycobacteria, *Chlamydia*, *E.coli* and Human HSP60) defined by Perschinka et al ([*2*](#_ENREF_2)). Gray background: Sequence homologous with the *Porphyromonas gingivalis* peptide of Jeong et al ([*3*](#_ENREF_3)), antibodies to which were more frequent in chronic periodontitis, atherosclerosis, type 2 diabetes mellitus and rheumatoid arthritis. Another peptide sequence, antibodies to which correlated with cardiovascular disease, Okada et al ([*4*](#_ENREF_4)) is shown with overstrike. Dark green background shows the peptide selectively recognized by patients with unstable angina or myocardial infarction, Wysocki et alc([*5*](#_ENREF_5)). The bold V in position 73 is mutated in Hereditary Spastic Paraplegia SPG13, labelled as position 72 in Hansen et al ([*6*](#_ENREF_6)). The underlined letters shows the autoepitopes to which antibodies were boosted by Coxsackievirus A9 infection, Härkönen et al. ([*7*](#_ENREF_7)).

A detailed discussion of peptidic HSP60 epitopes is given below (“Further details on HSP60 epitopes”).

| **Peptide name** | | **Peptide sequence** |
| --- | --- | --- |
| G1 | Human HSP60 1-30 | MLRLPTVFRQMRPVSRVLAPHLTRAYAKDV |
| G2 | Human HSP60 16-45 | RVLAPHLTRAYAKDVKFGADARALMLQGVD |
| G3 | Human HSP60 31-60 | KFGADARALMLQGVDLLADAVAVTMGPKGR |
| G4 | Human HSP60 46-75 | LLADAVAVTMGPKGRTVIIEQSWGSPKVTK |
| G5 | Human HSP60 61-90 | TVIIEQSWGSPK**V**TKDGVTVAKSIDLKDKY |
| G6 | Human HSP60 76-105 | DGVTVAKSIDLKDKYKNIGAKLVQDVANNT |
| G7 | Human HSP60 91-120 | KNIGAKLVQDVANNTNEEAGDGTTTATVLA |
| G8 | Human HSP60106-135 | NEEAGDGTTTATVLARSIAKEGFEKISKGA |
| G9 | Human HSP60121-150 | RSIAKEGFEKISKGANPVEIRRGVMLAVDA |
| G10 | Human HSP60136-165 | NPVEIRRGVMLAVDAVIAELKKQSKPVTTP |
| G11 | Human HSP60151-180 | VIAELKKQSKPVTTP~~EEIAQVATISANGDK~~ |
| G12 | Human HSP60166-195 | ~~EEIAQVATISANGDKEIGNII~~SDAMKKVGR |
| G13 | Human HSP60181-210 | EIGNIISDAMKKVGRKGVITVKDGKTLNDE |
| G14 | Human HSP60196-225 | KGVITVKDGKTLNDELEIIEGMKFDRGYIS |
| G15 | Human HSP60211-240 | LEIIEGMKFDRGYISPYFINTSKGQKCEFQ |
| G16 | Human HSP60226-255 | PYFINTSKGQKCEFQDAYVLLSEKKISSIQ |
| G17 | Human HSP60241-270 | DAYVLLSEKKISSIQSIVPALEIANAHRKP |
| G18 | Human GroEL256-285 | SIVPALEIANAHRKPLVIIAEDVDGEALST |
| G19 | Human GroEL271-300 | LVIIAEDVDGEALSTLVLNRLKVGLQVVAV |
| G20 | Human GroEL286-315 | LVLNRLKVGLQVVAVKAPGFGDNRKNQLKD |
| G21 | Human GroEL301-330 | KAPGFGDNRKNQLKDMAIATGGAVFGEEGL |
| G22 | Human GroEL316-345 | MAIATGGAVFGEEGLTLNLEDVQPHDLGKV |
| G23 | Human GroEL331-360 | TLNLEDVQPHDLGKVGEVIVTKDDAMLLKG |
| G24 | Human GroEL346-375 | GEVIVTKDDAMLLKGKGDKAQIEKRIQEII |
| G25 | Human GroEL361-390 | KGDKAQIEKRIQEIIEQLDVTTSEYEKEKL |
| G26 | Human GroEL376-405 | EQLDVTTSEYEKEKLNERLAKLSDGVAVLK |
| G27 | Human GroEL391-420 | NERLAKLSDGVAVLKVGGTSDVEVNEKKDR |
| G28 | Human GroEL406-435 | VGGTSDVEVNEKKDRVTDALNATRAAVEEG |
| G29 | Human GroEL421-450 | VTDALNATRAAVEEGIVLGGGCALLRCIPA |
| G30 | Human GroEL436-465 | IVLGGGCALLRCIPALDSLTPANEDQKIGI |
| G31 | Human GroEL451-480 | LDSLTPANEDQKIGIEIIKRTLKIPAMTIA |
| G32 | Human GroEL466-495 | EIIKRTLKIPAMTIAKNAGVEGSLIVEKIM |
| G33 | Human GroEL481-510 | KNAGVEGSLIVEKIMQSSSEVGYDAMAGDF |
| G34 | Human GroEL496-525 | QSSSEVGYDAMAGDFVNMVEKGIIDPTKVV |
| G35 | Human GroEL511-540 | VNMVEKGIIDPTKVVRTALLDAAGVASLLT |
| G36 | Human GroEL526-555 | RTALLDAAGVASLLTTAEVVVTEIPKEEKD |
| G37 | Human GroEL541-570 | TAEVVVTEIPKEEKDPGMGAMGGMGGGMGG |
| G38 | Human GroEL556-573 | PGMGAMGGMGGGMGGGMF |

**Table ST2.** The amino acid sequences of overlapping peptides of fragments around human HSP60 G15 (aa211-240) and human HSP60 G20 (aa286-320), shown in red.

| **Peptide name** | | **Peptide sequence** |
| --- | --- | --- |
| G15a | Human GroEL202-231 | KDGKTLNDELEIIEGMKFDRGYISPYFINT |
| G15b | Human GroEL205-234 | KTLNDELEIIEGMKFDRGYISPYFINTSKG |
| G15c | Human GroEL208-237 | NDELEIIEGMKFDRGYISPYFINTSKGQKC |
| G15 | Human GroEL211-240 | LEIIEGMKFDRGYISPYFINTSKGQKCEFQ |
| G15d | Human GroEL214-243 | IEGMKFDRGYISPYFINTSKGQKCEFQDAY |
| G15e | Human GroEL217-246 | MKFDRGYISPYFINTSKGQKCEFQDAYVLL |
| G15f | Human GroEL220-249 | DRGYISPYFINTSKGQKCEFQDAYVLLSEK |
| G20a | Human GroEL277-306 | DVDGEALSTLVLNRLKVGLQVVAVKAPGFG |
| G20b | Human GroEL280-309 | GEALSTLVLNRLKVGLQVVAVKAPGFGDNR |
| G20c | Human GroEL283-312 | LSTLVLNRLKVGLQVVAVKAPGFGDNRKNQ |
| G20 | Human GroEL286-320 | LVLNRLKVGLQVVAVKAPGFGDNRKNQLKD |
| G20d | Human GroEL289-318 | NRLKVGLQVVAVKAPGFGDNRKNQLKDMAI |
| G20e | Human GroEL292-321 | KVGLQVVAVKAPGFGDNRKNQLKDMAIATG |
| G20f | Human GroEL295-324 | LQVVAVKAPGFGDNRKNQLKDMAIATGGAV |

**Table ST3.** The amino acid sequences of GroEL of many microbes which are homologs of Human HSP60 (283-312) (Here referred to as “G20c homologs”); Peptide names contain the respective GenBank identity numbers)

| **Peptide name** | **Peptide sequence** |
| --- | --- |
| GroEL_orientia_tsutsugamushi_CH60_ORITS 258-287 | LTALILNNLKGSIKVVAVKAPGFGDRKKEM |
| GroEL5_neoehrlichia_mikurensis_FJ966359 259-288 | LSTLVLNKLRGGLHVAAVKAPGFGDRRKDM |
| GroEL_Wolbachia_YP_198181 261-290 | LSTLVINKLRGGLKVTAVKAPGFGDRRKEM |
| GroEL_Legionella_YP_126086 258-287 | LATLVVNNMRGIVKVCAVKAPGFGDRRKAM |
| GroEL_Borrelia_garinii_YP_073092 258-287 | LAALVLNSVRGALKVCAIKSPGFGDRRKAM |
| GroEL_Bartonella_henselae_YP_034075 259-288 | LATLVVNKLRGGLKIAAVKAPGFGDRRKAM |
| GroEL_tropheryma_whipplei_NP_789261 257-286 | LATLVVNKIRGIFKSVAVKAPGFGDRRKMM |
| GroEL_rickettsia_bellii_YP_537760 259-288 | LATLVVNRLRGGLKVAAVKAPGFGDRRKAM |
| GroEL_anaplasma_phagocytophilium_HZ_YP_504857 258-287 | LSTLVLNKLRGGLQVAAVKAPGFGDRRKDM |
| GroEL_Burkholderia_multivorans_YP_001947677 259-288 | LATLVVNAMRGILKVAAVKAPGFGDRRKAM |
| GroEL_E_coli_K12_AAC77103 259-288 | LATLVVNTMRGIVKVAAVKAPGFGDRRKAM |
| GroEL_CH60_CHLPN_chlamydia_pneumoniae 259-288 | LATLVVNRLRAGFRVCAVKAPGFGDRRKAM |
| GroEL_Staph_aureus_MRSA_YP_041479 257-286 | LTNIVLNRMRGTFTAVAVKAPGFGDRRKAM |
| GroEL_Mycobact_tuberc_CAA17397 257-286 | LSTLVVNKIRGTFKSVAVKAPGFGDRRKAM |
| GroEL_mycoplasma_penetrans_NP_757486 258-287 | LTTLVVNKMRGVFNVVAVKAPEFGDKRKQV |
| HSP60_Leishmania_Strain_Friedlin_XP_001685504 272-301 | MHTFLYNKIQGRISGCAVKAPGFGDMRINQ |
| HSP60_Schistosoma_mansoni_XP_002572332 276-305 | LTALVLNRLKLGLQVCAVKAPGFGDNRKNT |
| HSP60_Plasmodium_falciparum_XP_001347438 288-317 | LATLIVNKLRLGLKICAVKAPGFGEHRKAL |
| GroEL_Leptospira_interrogans_YP_001299 258-287 | LATIVVNTLRKTISCVAVKAPGFGDRRKSM |
| GroEL_Listeria_monocytogenes_AF335323_2 257-286 | QATLVLNKLRGTFNVVAVKAPGFGDRRKAM |
| GroEL_streptococcus_pneumoniae_AF325449_1 257-286 | LPTLVLNKIRGTFNVVAVKAPGFGDRRKAM |
| GroEL_treponema_pallidum_YP_001933036 258-287 | LATLVVNSLRGTLKTCAVKAPGFGDRRKEM |
| Chaperonin60_Giardia_lamblia_XP_001705532 261-290 | LSTLAINTLKGTVRCCAVRAPGYGDVKKGV |
| chaperonin60_Cryptosporidium_parvum_XP_627821 291-320 | LTALILNKLQLNLKVCAVKAPGFGDHRKQI |
| Chaperonin60_Entamoeba_histolytica_XP_656268 265-294 | LTTLVLNKLRGLPIAAVRAPGFGETRKGILH |

Results with the above peptides are shown in figure 2 with the following abbreviations:

The G20c homologs were 5. *Escherichia coli* (ESCHCOLI), 6. *Legionella pneumophila* (LEGIONELL)*,* 7. *Burkholderia multivorans* (BURKHMULT), 8. *Bartonella henselae* (BARTHENS), 9. *Borrelia garinii* (BORRGAR), 10. *Treponema pallidum* (TREPPALL),11. *Leptospira interrogans* (LEPTINTERR), 12*. Chlamydia pneumoniae* (CHLAMPNEUM), 13. *Mycobacterium tuberculosis* (MYCTUB), 14. *Tropheryma whipplei* (TROPHWHIP), 15. *Staphylococcus aureus* (STAPAUR), 16. *Listeria monocytogenes* (LISTMONO), 17. *Streptococcus pneumoniae* (STREPTPNEU), 18. *Mycoplasma penetrans* (MYCPEN), 19. *Neoehrlichia mikurensis* (NEOMIK), 20. *Anaplasma phagocytophilium* (ANAPPHAG), 21. *Wolbachia* (WOLBAC)*,* 22*. Orientia tutsugamishi* (ORITSU)*,* 23. *Rickettsia bellii* (RICKBELL)*,* 24*.* *Leishmania major* (LEISHMAN), 25*. Giardia lamblia* (GIARLAMB), 26. *Entamoeba histolytica* (ENTHIS), 27*. Plasmodium falciparum* (PLASFALC), 28. *Cryptosporidium parvum* (CRYPTPARV), 29. *Schistosoma mansoni* (SCHISMANS) (cf. Supplementary Table ST3).

All G20c homologs gave high NTC values (100-900 MFI). The values presented in Figure 2 were partially subtracted with the NTC value as described in Materials and methods. The subtraction was uniform, because equal numbers of patient and control samples were always analyzed together. It could therefore not create artificial statistical differences between the groups. Rather, occasional oversubtraction where negative values were set to 0 could reduce, not exaggerate, differences and significances. G20c homolog peptides with the highest positive predictive (100%) and negative predictive values (62-68%) for ME in the IgM test were *Staphylococcus* at cutoff 66 MFI, *Plasmodium* at cutoff 321 MFI, *Listeria* at cutoff 198 MFI, *Burkholderia* at cutoff 278 MFI, *Chlamydia* at cutoff 244 MFI and *Legionella* at cutoff 271 MFI.

**Table ST4.** The amino acid sequences of *Mycoplasma* penetrans GroEl peptides (GenBank id NP_757486).

| **Peptide name** | | **Peptide sequence** |
| --- | --- | --- |
| D1 | GroEL_Mycopl_pen 1-30 | MAKEIKFSDSARNKLFNGVQQLFDAVKVTM |
| D2 | GroEL_Mycopl_pen 16-45 | FNGVQQLFDAVKVTMGPRGRNVLIQKSYGA |
| D3 | GroEL_Mycopl_pen 31-60 | GPRGRNVLIQKSYGAPVITKDGVSVAKEVD |
| D4 | GroEL_Mycopl_pen 46-75 | PVITKDGVSVAKEVDLTNPIENMGAQLVKD |
| D5 | GroEL_Mycopl_pen 61-90 | LTNPIENMGAQLVKDVASKTADEAGDGTTT |
| D6 | GroEL_Mycopl_pen 76-105 | VASKTADEAGDGTTTATVLAYGVFKEGLRN |
| D7 | GroEL_Mycopl_pen 91-120 | ATVLAYGVFKEGLRNVISGANPIEIKRGMD |
| D8 | GroEL_Mycopl_pen 106-135 | VISGANPIEIKRGMDKTVNAIVNELNKSSK |
| D9 | GroEL_Mycopl_pen 121-200 | KTVNAIVNELNKSSKKIARKDEIIQVATIS |
| D10 | GroEL_Mycopl_pen 136-165 | KIARKDEIIQVATISANSDKKIGELIANAM |
| D11 | GroEL_Mycopl_pen 201-180 | ANSDKKIGELIANAMEKVGSDGVITVEEAK |
| D12 | GroEL_Mycopl_pen 166-195 | EKVGSDGVITVEEAKGINDELTVVEGMQFD |
| D13 | GroEL_Mycopl_pen 181-210 | GINDELTVVEGMQFDRGYISPYFVTDTNKM |
| D14 | GroEL_Mycopl_pen 196-225 | RGYISPYFVTDTNKMIAKLENPYILITDKK |
| D15 | GroEL_Mycopl_pen 211-240 | IAKLENPYILITDKKVSSIKDILPILEEIM |
| D16 | GroEL_Mycopl_pen 226-255 | VSSIKDILPILEEIMKTGRPLLIIADDVDG |
| D17 | GroEL_Mycopl_pen 241-270 | KTGRPLLIIADDVDGEALTTLVVNKMRGVF |
| D18 | GroEL_Mycopl_pen 256-285 | EALTTLVVNKMRGVFNVVAVKAPEFGDKRK |
| D19 | GroEL_Mycopl_pen 271-300 | NVVAVKAPEFGDKRKQVLEDIAILTGGSFV |
| D20 | GroEL_Mycopl_pen 286-320 | QVLEDIAILTGGSFVTDDLGISFDKVTLQD |
| D21 | GroEL_Mycopl_pen 301-330 | TDDLGISFDKVTLQDLGQAESVVIDKDNST |
| D22 | GroEL_Mycopl_pen 316-345 | LGQAESVVIDKDNSTIVKGKGLESQIKERI |
| D23 | GroEL_Mycopl_pen 331-360 | IVKGKGLESQIKERISKIKTAIEMTDSDYD |
| D24 | GroEL_Mycopl_pen 346-375 | SKIKTAIEMTDSDYDKDSLRNRLAKLNKGV |
| D25 | GroEL_Mycopl_pen 361-390 | KDSLRNRLAKLNKGVAVIKVGAVSEVELKE |
| D26 | GroEL_Mycopl_pen 376-405 | AVIKVGAVSEVELKEKKDRVDDALSATKAA |
| D27 | GroEL_Mycopl_pen 391-420 | KKDRVDDALSATKAAIEEGIVIGGGAALVH |
| D28 | GroEL_Mycopl_pen 406-435 | IEEGIVIGGGAALVHVSKRINVNTLNLIGD |
| D29 | GroEL_Mycopl_pen 421-450 | VSKRINVNTLNLIGDEKIGYQIVMSAIMSP |
| D30 | GroEL_Mycopl_pen 436-465 | EKIGYQIVMSAIMSPISQIVSNAGFDKGVV |
| D31 | GroEL_Mycopl_pen 451-480 | ISQIVSNAGFDKGVVINEILKATNPHLGFN |
| D32 | GroEL_Mycopl_pen 466-495 | INEILKATNPHLGFNAATGKYVDMFQTGII |
| D33 | GroEL_Mycopl_pen 481-510 | AATGKYVDMFQTGIIDPVKVTRIALQNAVS |
| D34 | GroEL_Mycopl_pen 496-525 | DPVKVTRIALQNAVSVSSMLLTTEAVIYDV |
| D35 | GroEL_Mycopl_pen 511-540 | VSSMLLTTEAVIYDVKDDKEDSVPAMPNMG |
| D36 | GroEL_Mycopl_pen 526-555 | KDDKEDSVPAMPNMGMGGMM |

**Table ST5.**  The amino acid sequences of overlapping *Chlamydia pneumoniae* GroEL (GenBank id NP_224342) peptides. The peptides were synthesized with short (tri-ethylene glycol, C series) and long (hexa-ethylene glycol, E series) spacers.

Yellow background: Peptides recognized by IgG in sera from women with *Chlamydia trachomatis* associated ectopic pregnancy, Yi et al ([*8*](#_ENREF_8)). Most *Chlamydia pneumoniae* peptides had an identical sequence. Positions which differed were underlined. The overlapping epitopes of peptides C30-C32 and E30-E32 defined by Yi et al were indicated by additional overstrike and italic marks.

| **Short**  **Spacer** | **Long**  **Spacer** | **Peptide name** | **Peptide sequence** |
| --- | --- | --- | --- |
| C1 | E1 | GroEL_Chlamyd_pneum 1-30 | MVAKNIKYNEEARKKIQKGVKTLAEAVKVT |
| C2 | E2 | GroEL_Chlamyd_pneum 16-45 | IQKGVKTLAEAVKVTLGPKGRHVVIDKSFG |
| C3 | E3 | GroEL_Chlamyd_pneum 31-60 | LGPKGRHVVIDKSFGSPQVTKDGVTVAKEV |
| C4 | E4 | GroEL_Chlamyd_pneum 46-75 | SPQVTKDGVTVAKEVELADKHENMGAQMVK |
| C5 | E5 | GroEL_Chlamyd_pneum 61-90 | ELADKHENMGAQMVKEVASKTADKAGDGTT |
| C6 | E6 | GroEL_Chlamyd_pneum 76-105 | EVASKTADKAGDGTTTATVLAEAIYTEGLR |
| C7 | E7 | GroEL_Chlamyd_pneum 91-120 | TATVLAEAIYTEGLRNVTAGANPMDLKRGI |
| C8 | E8 | GroEL_Chlamyd_pneum 106-135 | NVTAGANPMDLKRGIDKAVKVVVDQIKKIS |
| C9 | E9 | GroEL_Chlamyd_pneum 121-200 | DKAVKVVVDQIKKISKPVQHHKEIAQVATI |
| C10 | E10 | GroEL_Chlamyd_pneum 136-165 | KPVQHHKEIAQVATISANNDAEIGNLIAEA |
| C11 | E11 | GroEL_Chlamyd_pneum 201-180 | SANNDAEIGNLIAEAMEKVGKNGSITVEEA |
| C12 | E12 | GroEL_Chlamyd_pneum 166-195 | MEKVGKNGSITVEEAKGFETVLDVVEGMNF |
| C13 | E13 | GroEL_Chlamyd_pneum 181-210 | KGFETVLDVVEGMNFNRGYLSSYFATNPET |
| C14 | E14 | GroEL_Chlamyd_pneum 196-225 | NRGYLSSYFATNPETQECVLEDALVLIYDK |
| C15 | E15 | GroEL_Chlamyd_pneum 211-240 | QECVLEDALVLIYDKKISGIKDFLPILQQV |
| C16 | E16 | GroEL_Chlamyd_pneum 226-255 | KISGIKDFLPILQQVAESGRPLLIIAEDIE |
| C17 | E17 | GroEL_Chlamyd_pneum 241-270 | AESGRPLLIIAEDIEGEALATLVVNRIRGG |
| C18 | E18 | GroEL_Chlamyd_pneum 256-285 | GEALATLVVNRIRGGFRVCAVKAPGFGDRR |
| C19 | E19 | GroEL_Chlamyd_pneum 271-300 | FRVCAVKAPGFGDRRKAMLEDIAILTGGQL |
| C20 | E20 | GroEL_Chlamyd_pneum 286-320 | KAMLEDIAILTGGQLISEELGMKLENANLA |
| C21 | E21 | GroEL_Chlamyd_pneum 301-330 | ISEELGMKLENANLAMLGKAKKVIVSKEDT |
| C22 | E22 | GroEL_Chlamyd_pneum 316-345 | MLGKAKKVIVSKEDTTIVEGMGEKEALEAR |
| C23 | E23 | GroEL_Chlamyd_pneum 331-360 | TIVEGMGEKEALEARCESIKKQIEDSSSDY |
| C24 | E24 | GroEL_Chlamyd_pneum 346-375 | CESIKKQIEDSSSDYDKEKLQERLAKLSGG |
| C25 | E25 | GroEL_Chlamyd_pneum 361-390 | DKEKLQERLAKLSGGVAVIRVGAATEIEMK |
| C26 | E26 | GroEL_Chlamyd_pneum 376-405 | VAVIRVGAATEIEMKEKKDRVDDAQHATIA |
| C27 | E27 | GroEL_Chlamyd_pneum 391-420 | EKKDRVDDAQHATIAAVEEGILPGGGTALI |
| C28 | E28 | GroEL_Chlamyd_pneum 406-435 | AVEEGILPGGGTALIRCIPTLEAFLPMLTN |
| C29 | E29 | GroEL_Chlamyd_pneum 421-450 | RCIPTLEAFLPMLTNEDEQIGARIVLKALS |
| C30 | E30 | GroEL_Chlamyd_pneum 436-465 | EDEQIGARIVLKALSAPLKQIAA~~NAGKEGA~~ |
| C31 | E31 | GroEL_Chlamyd_pneum 451-480 | APLKQIAA~~NAGKE~~*~~GAIIFQQ~~VMSRS*ANEGY |
| C32 | E32 | GroEL_Chlamyd_pneum 466-495 | *IIFQQVMSRS*ANEGYDALRDAYTDMLEAGI |
| C33 | E33 | GroEL_Chlamyd_pneum 481-510 | DALRDAYTDMLEAGILDPAKVTRSALESAA |
| C34 | E34 | GroEL_Chlamyd_pneum 496-525 | LDPAKVTRSALESAASVAGLLLTTEALIAE |
| C35**^(m)^** | E35**^g(m)^** | GroEL_Chlamyd_pneum 511-540 | SVAGLLLTTEALIAEIPEEKPAAAPAMPGA |
| C36 | E36 | GroEL_Chlamyd_pneum 526-555 | IPEEKPAAAPAMPGAGMDY |

Polyethylene glycol of varying length has earlier been used as an inert spacer to display peptides with minimal spatial constraint on surfaces ([*9*](#_ENREF_9)). The spacer can restrict the availability of a peptide to antibodies. Potentially it could lead to false negative results in SMIA, and we wanted to investigate this variable. It turned out that an IgG epitope that was not visible in the scan with Chlamydia peptides with a short spacer was prominent in the scan with a longer spacer (E35). On the other hand, an epitope which was relatively strongly recognized with a short spacer (C25) was weaker with a long spacer. All peptides were anchored at the amino terminal end. It is possible that further epitopes could have been discovered if peptides anchored at the carboxy terminus also were tested.

**Supplementary table ST6.** Sequences of ”strongly binding peptides” (SBPs), taken from Chen and Sigler ([*1*](#_ENREF_1)). In that publication, the peptides were selected by panning with E. coli HSP60 from a peptide library. The biotinylated hexaethylene glycol spacer is written as Bio-(EG)_6_.

| **Peptide name** | **Peptide sequence** |
| --- | --- |
| SBP1 | Bio-(EG)_6_- SWMTTPWGFLHP |
| SBP2 | Bio-(EG)_6_- FHYEIWIPPHRG |
| SBP3 | Bio-(EG)_6_- SSPWWLVSFTST |
| SBP4 | Bio-(EG)_6_- SHSLIWRIPLLH |
| SBP5 | Bio-(EG)_6_- IYVPWYYAENLP |

**Section 3. Details regarding the SMIA procedure**

*Coupling of proteins and peptides to carboxylated microspheres*

The synthetic peptides or protein were coupled to Luminex Carboxylated Microspheres, as described in the “Sample protocol for two step carbodiimide coupling of protein to carboxylated microspheres” provided by the Luminex Corporation (Austin, TX). Briefly, the stock uncoupled beads (xMAP Technology, Austin, TX) were vortexed for 20 s, then sonicated for 20 s. An appropriate amount (i.e. 100 - 400 µl) of the stock microspheres, containing 1.25 × 10^7^ beads per ml, was transferred to a 1.5 ml Eppendorf tube which then was centrifuged at 13,000 × *g* for 3 min. The supernatant was carefully removed and the beads were resuspended in 100 μl of distilled water, followed by vortexing and sonication for 20 s, and then centrifuged at 13,000 ×*g* for 3 min. Supernatants were subsequently removed and resuspended in 80 µl of 100 mM monobasic sodium phosphate (MSP, Sigma, cat. nr S3139) pH 6.2 , then vortexed and sonicated for 20 s. Ten microliters of freshly made *N*-hydroxysuccinimide (NHS)(Pierce, cat. nr 24510) and 10 μl of 50-mg/ml 1-ethyl-3-(3-dimethylaminopropyl) carbodiimide (EDC; water-soluble carbodiimide; Pierce, cat. nr 25952-53-8 ) in H_2_O were added to the beads. The suspension was then incubated in the dark for 20 min at room temperature. The beads were then centrifuged as described above. The supernatant was carefully removed, and the pellet was washed twice in 250 μl of 50 mM 2-(*N*-morpholino) ethanesulfonic acid (MES) sodium salt (Sigma, cat. nr 71119-23-8), pH 5. The supernatant was removed and the beads resuspended in an appropriate volume of the MES buffer (i.e. 125 µl MES to 100µl of beads), vortexed and sonicated. Fifty micrograms of the peptide or 1- 100 µg protein was added. Beads were mixed by careful vortexing and then incubated in the dark for 2 hours at room temperature on a plate shaker (100 rpm). The coupled microspheres were pelleted by centrifugation at 13,000 × *g* for 3 min and resuspended in 500 μl of StabilGuard buffer (SurModics, Eden Prairie, MN, cat. nr SG01-1000 ). The coupled microspheres were again pelleted by centrifugation as described above and resuspended in 1 ml of StabilGuard buffer twice. The final pellet was resuspended in an appropriate amount of StabilGuard buffer (1000 µl of StabilGuard buffer if 100µl of beads were taken). This created a bead mixture consisting of 1250 beads/μl. The coupled beads were stored at 4°C in the dark.

Ten µg of *Haemophilus influenzae* type b (Hib) vaccine (Act-HIB®; Sanofi Pasteur MSD) was diluted in 100µL sterile water and coupled to the Luminex bead following the coupling protocol. An unprocessed (“naked”) bead, without any bound antigen, was used to control for nonspecific binding to the Luminex beads. The stock uncoupled beads were vortexed for 20 s, then sonicated for 20 s. An appropriate amount of the stock microspheres was transferred to a 1.5 ml Eppendorf tube which then was centrifuged at 13,000 × *g* for 3 min. The supernatant was carefully removed and the beads were resuspended in 100 μl of distilled water, followed by vortexing and sonication for 20 s, and then centrifuged at 13,000 ×*g* for 3 min. Supernatants were subsequently removed and resuspended in 500 µl of StabilGuard. The uncoupled beads were again pelleted by centrifugation as described above and resuspended in 1 ml of StabilGuard buffer twice. The final pellet was resuspended in StabilGuard buffer to 1250 beads/μl. The coupled beads in this stock suspension were stored at 4°C in the dark.

*Preanalytical procedures*

Samples were immediately frozen in small aliquots, to avoid repetitive freezing and thawing.

*Suspension Multiplex Immunoassay (SMIA)*

During the development of SMIA for many of the pathogens previously associated with ME we noted that the sensitivity of the SMIA was the same as, or better than, EIA, with a wide dynamic range and possibility to simultaneously read 100 analytes in the Luminex® 200™ analyzer (Luminex Corporation, Austin, Texas) (J Blomberg, unpublished, and ([*10*](#_ENREF_10)*,* [*11*](#_ENREF_11)). The assay system required only 5-10 µl of serum or plasma. Antigen (10-50 µg of whole microbe lysate, recombinant protein and synthetic peptide) was coupled covalently to carboxylated color-coded beads as described above. IgG was detected using biotinylated protein G (BPG) ([*12*](#_ENREF_12)*,* [*13*](#_ENREF_13)) as secondary antibody. For IgM antibody detection biotinylated anti-human IgM (affinity purified, μ-chain specific, Sigma-Aldrich cat. Nr. B1140) was used as secondary antibody.

A few control experiments with 4 µg/ml biotinylated monoclonal anti-IgG (BioLegend inc, San Diego, CA, cat. nr. 409307) and 4 µg/ml biotinylated monoclonal anti-IgM (BioLegend, cat nr. 314504) are reported in the Supplementary Figure SF3.

*Preparations prior to filter plate loading*

Samples were diluted prior to loading the filter plate using StabilGuard as a diluent. Each ME patient plasma sample was diluted 1:10 (this dilution included the dilution during plasma preparation). BD serum samples were also diluted 1:10. After a concluded coupling the bead stock solution contained 1250 beads/ µl. For a typical experiment, a bead mixture consisting of 25 beads/μl was made using StabilGuard as a diluent. All the beads with coupled proteins or peptides as well as the naked bead and *Hemophilus influenzae* B bead were sonicated and vortexed for 20 s before being added to the bead mixture. A fetuin bead (see below) was included in IgM experiments as an additional positive control.

*Filter plate loading and washing*

For an IgG run, the standard Luminex protocol for the filter plate, namely “Sample protocol for indirect antibody capture immunoassay” was largely followed ([*10*](#_ENREF_10)*,* [*11*](#_ENREF_11)), with the exception that coupling conditions were different. Briefly, the multiplex assay was carried out in a 96 well Multiscreen® filter plates (Millipore, UK, cat. nr MSHVN4B10) with 0.45 μm pore size. Initially, the filter plate was pre-wetted twice with 100 µl PBS (pH7.4).The PBS was removed from the wells by aspiration through the MultiScreen_HTS_ vacuum manifold (Millipore, USA, cat. nr. MSVMHTS0D). Fifty microliters of serum diluted 1:10 in StabilGuard buffer were added to each well, except one, a well containing only StabilGuard and beads, as a non-template control (NTC). The bead mixture was sonicated and vortexed for 20 s. Fifty microliters of the bead mix was then added to each well. After this, the wells were incubated in the dark for 30 min at room temperature on a plate shaker. After incubation, the plate was aspirated and washed with 100 µl PBS twice. Fifty µl StabilGuard were added and followed by 50µl of a 4µg/ml BPG solution (Pierce, cat. nr 29988) diluted in StabilGuard. The beads were resuspended by pipetting up and down five times. The plate was incubated in the dark for 30 min at room temperature on a plate shaker (100 rpm). After incubation, the wells were aspirated and washed twice with 100 µl PBS. 50µl StabilGuard were added to each well and followed by 50 µl of a 4 μg/ml streptavidin-phycoerythrin (SA-PE) solution (Life Technologies Europe, cat. nr SA1004-4) diluted in StabilGuard, and the beads were resuspended by pipetting up and down five times. The plate was then incubated in the dark for 15 min at room temperature on a plate shaker. After incubation, the wells were aspirated and washed twice with 100 μl PBS. The filter plate well was resuspended in 150 μl of PBS. One hundred μl were then analyzed on the Luminex® 200™ system analyzer following the manufacturer's instructions. The StarStation (Applied Cytometry, Sheffield, UK) or xPONENT® (Luminex corp. Austin, Tx) softwares were used to analyze the data.

For an IgM run, filter plate loading was done as described for IgG, with the exception that 0.4 µg/ml of anti-human IgM (Biotin conjugated, μ-chain specific, Sigma-Aldrich, cat. nr. B1265) was used as a secondary antibody instead of Protein G. The HIB vaccine bead, intended as a positive control for IgG detection, proved to work also as a positive control for IgM detection. The HIB vaccine is a polysaccharide conjugated to tetanus toxoid, and it is not surprising that it also detects IgM, because anti-carbohydrate antibodies tend to be of IgM type. As a further positive control for presence of IgM (Katona and Blomberg, unpublished), we used a bead coupled with 100 μg of bovine fetuin (Sigma-Aldrich, cat. nr F2379), also called a2HS-Glycoprotein. Fetuin is a heavily glycosylated glycoprotein which reacts with anti-betagalactoside antibodies. The majority of humans have such IgM antibodies.

*Specificity of the MFI signals*

**Supplementary figure SF1.**

1. NTC Protein G vs NTC monoclonal anti IgG for 24 human HSP60 peptides (G1-G24).
2. Correlation between protein G and monoclonal anti-IgG values for a HIB-coupled bead, and 24 ME samples.
3. Correlation between protein G and monoclonal anti-IgG for a G20c-coupled bead, and 22 ME samples.

1. Correlation between poly- and monoclonal anti-IgM for a HIB-coupled bead and 39 ME samples.

**
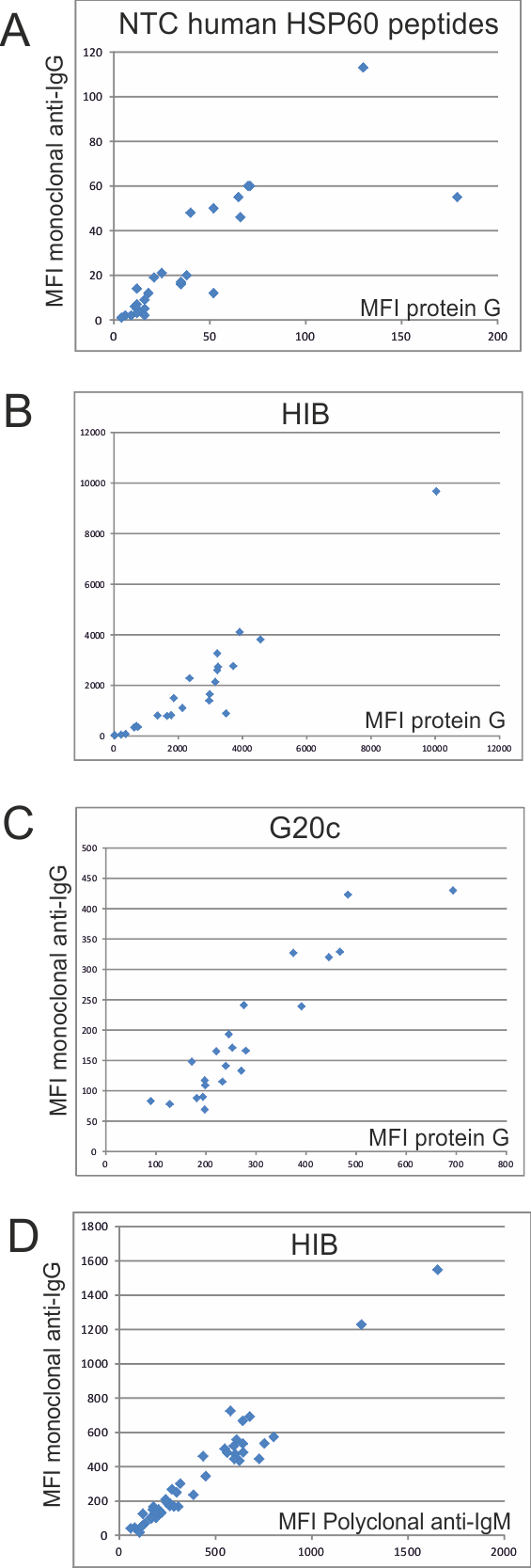
**

In experiments using Strongly Binding Peptides, SBPs, the five biotinylated SBPs (SBP1-SBP5, see Supplementary Table ST6) were dissolved in sterile phosphate-buffered saline (PBS), pH 7 and 10 to 50% DMSO. For a Strongly Binding Peptide assay, filter plate loading was done as described for IgG, with the exception that 500, 50, 5 and 0.5 µg/ml of each five biotinylated SBP (SBP1-SBP5) was used instead of Protein G. Fifty microliters of sample (IgG diluted 1:10, ME pool diluted 1:5, and ME pool diluted 1:10) diluted in StabilGuard buffer was added to each well. One well containing only StabilGuard was used as a non-template control “NTC” well. Fifty microliters of the bead mix (containing beads with human HSP60 G20, G20a, G20b, G20c, G20d, G20e, G20f, recombinant human HSP60, GroEL recombinant protein, *Hemophilus influenzae* B, and naked beads) was then added to each well. Four different concentrations for each of the five biotinylated SBPs (500, 50, 5 and 0.5 µg/ml) were incubated for 30 minutes. The beads were then washed, and incubated with SA-PE like in the experiments with biotinylated protein G and anti human IgM, and read in the Luminex bead counter. The results are described in Supplementary Figure SF2.

**Supplementary figure SF2.** Binding of “strongly binding peptides” (SBPs) to HSP60 recombinant proteins and human HSP60 G20 and G20c peptides.

1. Out of the five SBPs, SBP2 and SBP4, but not the other SBPs, bound to recombinant human HSP60, E coli GroEL, G20c and G20c homolog peptides, suggesting a chaperonin-like peptide binding activity of G20c homolog peptides. Signals of ME sera with high MFI values to these antigens were not much diminished.

B and C. SBP2 and SBP4 bound without saturation effects, with a concentration of 5 µg/ml being optimal for further experiments.


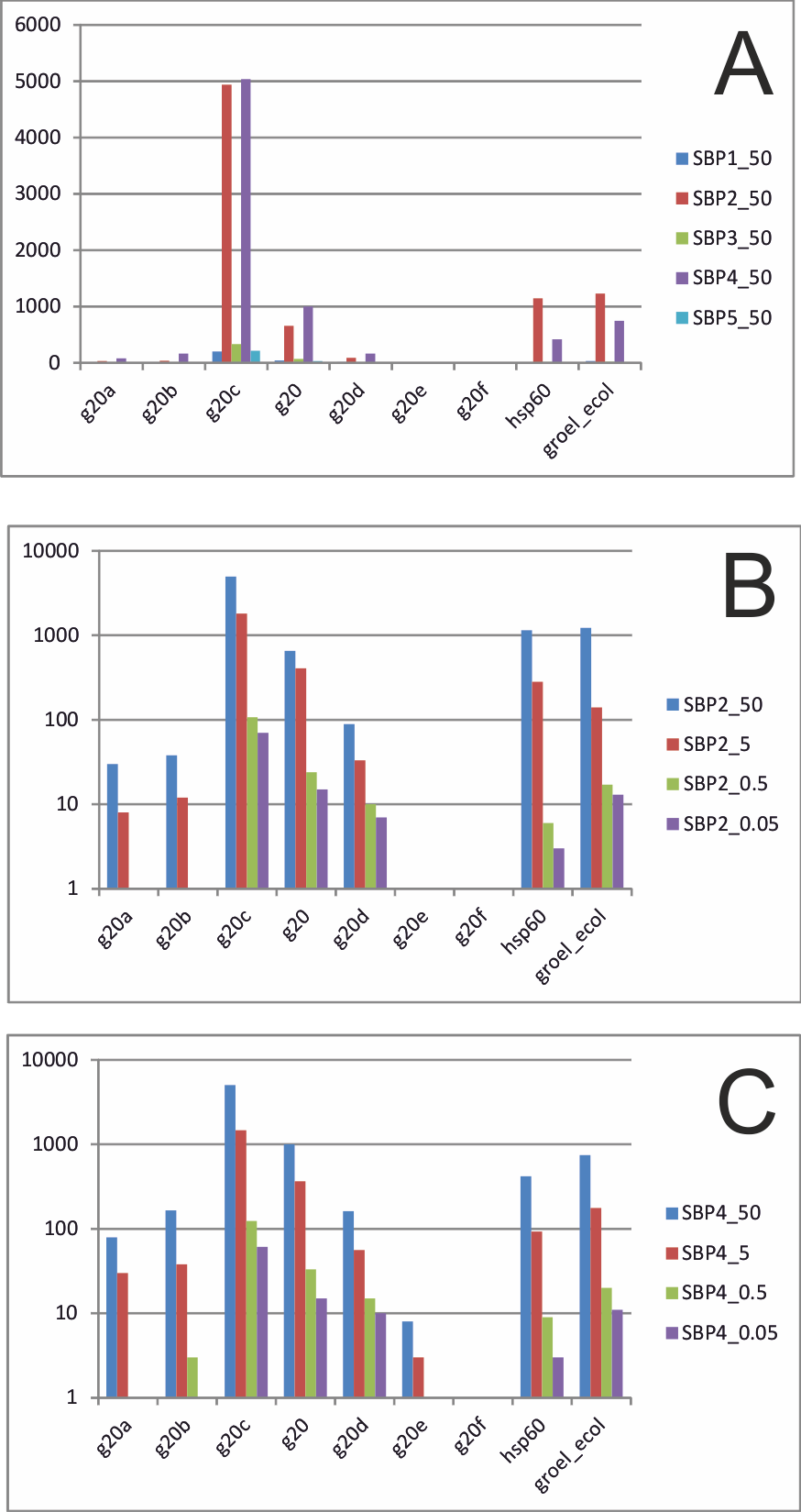


*Absorption of IgG with protein A.*

A source of falsely positive IgM values is IgG anti-IgM (“rheuma factor”), which occurs in autoimmune diseases like rheumatoid arthritis. Ten strongly IgM positive and 6 weakly IgM positive ME samples and 6 blood donor sera were incubated with recombinant Protein A Sepharose™ Fast Flow and Sepharose™ Fast Flow beads alone (17-1279-01, 17-0149-01, GE Healthcare, Bio-Sciences AB Uppsala, Sweden) at 37°C on a shaker for 1 h. The samples were centrifugated for 3 minutes at 13000 rpm. The supernatant was subsequently tested in the IgG and IgM SMIA tests, as described in Methods. In the IgG test, 0-30% of the MFI were recovered, while 60-150% of the MFI were recovered in the IgM test (data not shown). The result makes it unlikely that rheuma factor was a major cause of the IgM values in the ME samples.

*Competition experiments using blocking protein and SBPs.*

During the course of this investigation we found that including ovalbumin together with the secondary antibody drastically reduced the NTC values with a marginal effect on the antibody specific signals from the samples (Supplementary Figure SF2). Obviously, the human blood samples contained enough non-antibody protein (a typical serum or plasma contains 70 mg/ml) to block the low affinity detected by the NTC. The StabilGuard diluent does not contain protein. We did not routinely include highly concentrated blocking protein in the tests because of occasional clogging of the probe in the Luminex machine in the presence of blocking protein. Instead, we opted for a partial subtraction of NTC values, as described in Materials and Methods (Supplementary Figure SF3).

# A few peptides, mostly those containing the helix I of human and microbial HSP60s gave high NTC values, making this control less useful. The binding in the presence of patient sample could be lower than that of the NTC (binding of the signal generating system to the bead without presence of patient sample). We therefore tried to reduce the NTC values by addition of 0.1, 1, 10 and 100 µg/ml Ovalbumin (Albumin from chicken egg white), (Sigma-Aldrich, cat. nr A 5503) and Bovine Serum Albumin (Bovine Plasma Albumin; BSA), (Sigma-Aldrich, cat. nr A3913) during incubation with BPG or SA-PE. A tenfold reduction of the NTC value of bead-bound G20c, without affecting the MFI of wells containing patient samples, was obtained by inclusion of 100 µg/mL of ovalbumin during the incubation with BPG while BSA was less effective. However, the ovalbumin was not routinely added because of occasional clogging of the Luminex machine probe. NTC MFI were therefore partially subtracted from the MFI of patient and BD samples as described above.

# Competition experiments with blocking proteins were conducted using 0.1, 1, 10 and 100 µg/ml ovalbumin and the same concentrations of BSA (not shown) during incubation with BPG or SA-PE during the incubations with protein G and SA-PE. The results are described in Supplementary Figure SF3.

**Supplementary Figure SF3.** Competition experiments with high concentrations of blocking protein. Ovalbumin (and BSA, not shown) was added at the protein G and SAPE incubation steps. The IgG preparation (5 mg/ml), the ME pool and ME patient and a BD sample with high IgG binding to the three antigens were run without and with different concentrations of ovalbumin at the protein G/anti-IgM incubation stage. The effect of ovalbumin was stronger than that of BSA at the same weight percentages. Results for peptides G20 (A), G20c (B) and *E. coli* GroEL (HSP60) (C) are shown.

**
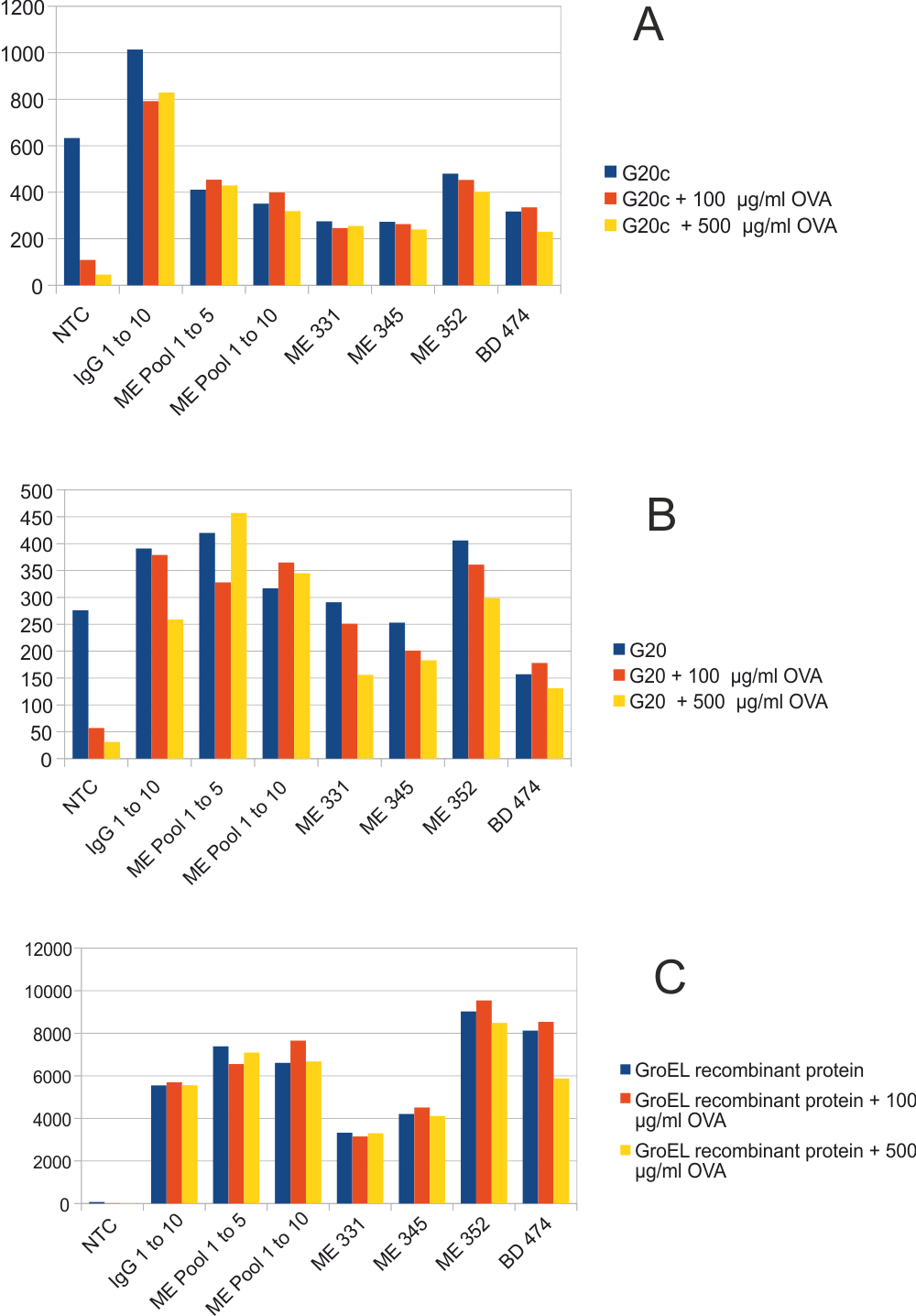
**

*Data management and Statistics*

A computer program (MultiPlus™) which subtracts the NTC and naked bead values, and checks the results of the control sera for each peptide and protein, and stores them in a relational database, was written in Visual Foxpro by J. Blomberg. The subtraction was trivial for nearly all naked bead values, which were 5-10 MFI, but nontrivial for a few peptides, where the NTC values could reach several hundred MFI. In these cases the lowest MFI obtained with some samples were often lower than the NTC value. To avoid oversubtraction, the subtraction was made with 10% of the NTC and 90% of the lowest sample value for the bead. Negative values after subtraction were set to 0 MFI. The experiments always contained an equal number of patient and control samples, so the two groups were always treated equally. The validity of the subtraction was ascertained in later experiments, when we found that the NTC values could be reduced by inclusion of ovalbumin during the incubation with BPG or biotinylated anti-IgM. Subtracted results with and without the ovalbumin inclusion were highly similar (Supplementary Figure SF1). Significant differences in antigen reactivity between ME patient and BD samples were automatically evaluated using the two-tailed Fisher exact test (FET) and two-tailed Wilcoxon rank sum tests (WRST) with computer procedures written by J. Blomberg. A cutoff of 200 MFI was used in the FET unless stated otherwise. To reduce the influence of weak binding values in the WRST, all MFI values were subtracted with 50, with resulting negative values listed as 0, before WRST analysis. At this initial stage, no (“Bonferroni”) correction of p values for repeated comparisons was made. Cross-correlation of variables to each other, and principal component analysis (PCA), was performed using the Unscrambler statistical package (version X, Camo AS, Bergen, Norway).

**Section 4. Further results from the phyloscanning with G20c homologs**

**Supplementary Figure SF5.** Needle plots of reactivities with Test set samples with G20c homolog peptides in A. IgG and B. IgM assays. MFI values subtracted with NTC MFI of selected G20c homologs from the phyloscanning. Results from G20c homologs of a protozoon and bacteria are presented separately. Although the ME preference in most IgG tests was inferior to that of the IgM tests it is shown for comparison. The patient group was here split into ME (lane 1), FM (lane 2), ME+FM (lane 3), ME+IBS (lane 4), ME+FM+IBS (lane 5) and FM+IBS (lane 6) groups. BD samples were in lane 7 and control samples in lane 0. The most strongly discriminating peptides are framed in red. A cutoff which may be useful if the peptide is to be used as a biomarker,based on the Test set alone, is also shown in red. For abbreviations, see the legend of Figure 5.

**
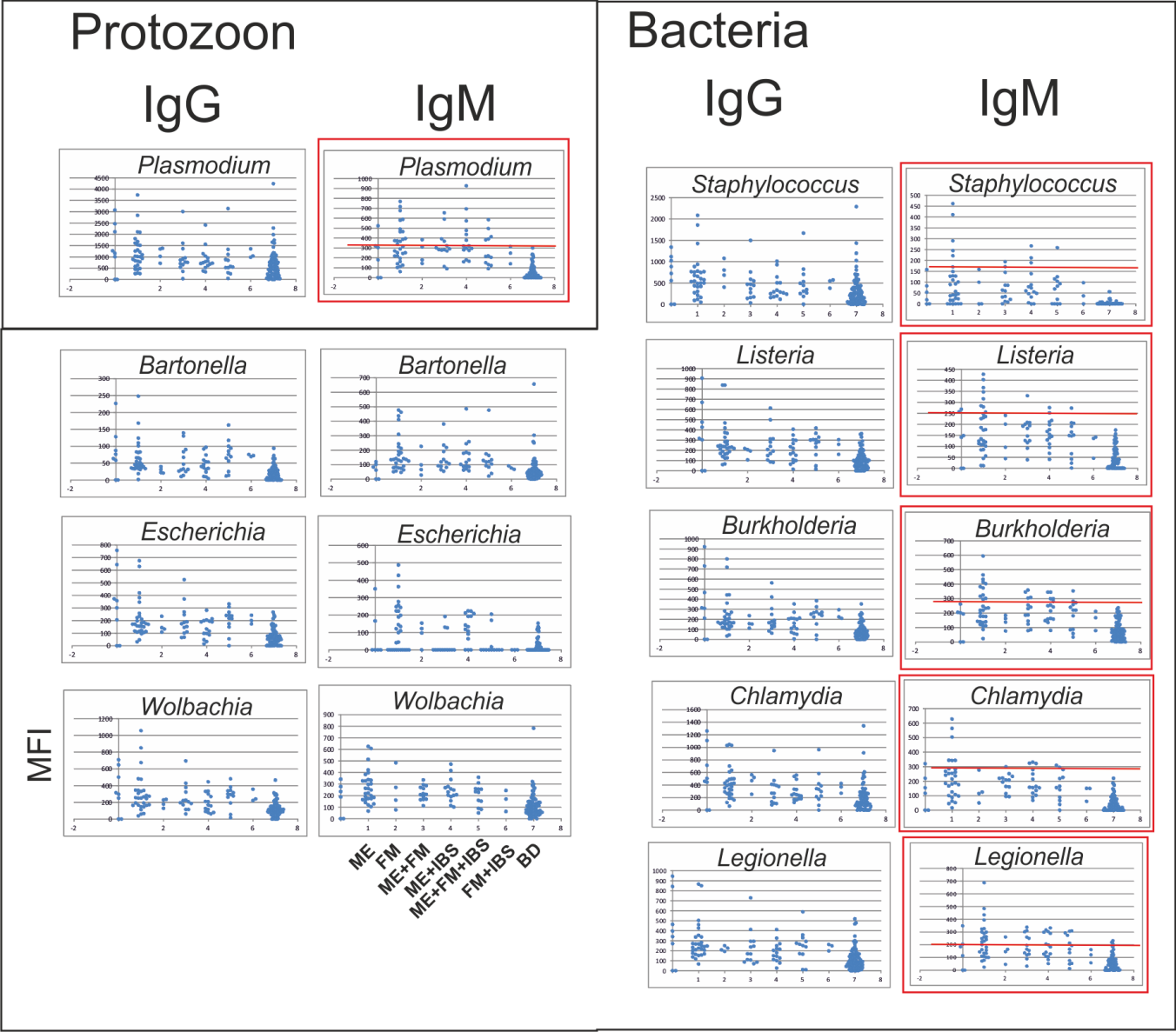
**

**Supplementary table ST7.** Correlation coefficients of MFI of G20c homolog peptides and recombinant human and *E. coli* HSP60 proteins in a serological assay with patient and BD samples. Correlation coefficients and sequence similarity according to the BLOSUM62 scoring system (“blosum”) are included.

**Table ST7A.** The 20 highest correlations between the IgG binding (MFI) of G20c homologs.

Five of the combinations were discordant, between a prokaryotic and a eukaryotic HSP60 sequence (*).

| Sequence 1 | Sequence 2 | Discordant  Combination | blosum | Iggcorr | Igmcorr |
| --- | --- | --- | --- | --- | --- |
| *Escherichia coli* | *Burkholderia multivorans* |  | 135 | 0,99228 | 0,64098 |
| *Treponema pallidum* | *Mycobacterium tuberculosis* |  | 116 | 0,96978 | 0,39078 |
| *Leptospira interrogans* | *Legionella pneumophila* |  | 99 | 0,96167 | 0,78119 |
| *Burkholderia multivorans* | *Bartonella henselae* |  | 126 | 0,93848 | 0,55282 |
| *Escherichia coli* | *Bartonella henselae* |  | 124 | 0,93809 | 0,27021 |
| *Escherichia coli* | *Legionella pneumophila* |  | 135 | 0,93669 | 0,5946 |
| *Leptospira interrogans* | *Chlamydia pneumoniae* |  | 102 | 0,93266 | 0,84728 |
| *Anaplasma phagocytophilium* | *Wolbachia* |  | 132 | 0,93076 | 0,42341 |
| *Staphylococcus aureus* | *Homo sapiens* | * | 90 | 0,92763 | 0,46618 |
| *Schistosoma mansonii* | *Homo sapiens* |  | 127 | 0,91305 | 0,37872 |
| *Rickettsia bellii* | *Candidatus neoehrlichia mikurensis* |  | 124 | 0,91003 | 0,51955 |
| *Leptospira interrogans* | *Escherichia coli* |  | 104 | 0,90108 | 0,66396 |
| *Chlamydia pneumoniae* | *Homo sapiens* | * | 100 | 0,89957 | 0,52151 |
| *Leptospira interrogans* | *Burkholderia multivorans* |  | 100 | 0,89639 | 0,87349 |
| *Leptospira interrogans* | *Plasmodium falciparum* | * | 92 | 0,89507 | 0,46447 |
| *Leptospira interrogans* | *Homo sapiens* | * | 89 | 0,89501 | 0,45683 |
| *Anaplasma phagocytophilium* | *Bartonella henselae* |  | 130 | 0,887 | 0,69313 |
| *Leptospira interrogans* | *Staphylococcus aureus* |  | 100 | 0,882 | 0,81089 |
| *Leptospira interrogans* | *Schistosoma mansonii* | * | 77 | 0,87907 | 0,66144 |
| *Borrelia_garinii* | *Homo sapiens* | * | 83 | 0,86963 | 0,38378 |

**Table ST7B.** The 20 highest correlations between the IgM binding (MFI) of G20c homologs. One of the combinations was discordant, between a prokaryotic and a eukaryotic HSP60 sequence (*).

| Sequence 1 | Sequence 2 | Discordant  combination | blosum | Iggcorr | Igmcorr |
| --- | --- | --- | --- | --- | --- |
| *Borrelia garinii* | *Legionella pneumophila* |  | 123 | 0,86684 | 0,93864 |
| *Rickettsia bellii* | *Bartonella henselae* |  | 141 | 0,72258 | 0,87917 |
| *Leptospira interrogans* | *Burkholderia multivorans* |  | 100 | 0,89639 | 0,87349 |
| *Rickettsia bellii* | *Tropheryma whipplei* |  | 109 | 0,7994 | 0,86233 |
| *Chlamydia pneumoniae* | *Burkholderia multivorans* |  | 110 | 0,71103 | 0,85463 |
| *Leptospira interrogans* | *Chlamydia pneumoniae* |  | 102 | 0,93266 | 0,84728 |
| *Treponema pallidum* | *Legionella pneumophila* |  | 124 | 0,741 | 0,84472 |
| *Leptospira interrogans* | *Staphylococcus aureus* |  | 100 | 0,882 | 0,81089 |
| *Leptospira interrogans* | *Legionella pneumophila* |  | 99 | 0,96167 | 0,78119 |
| *Mycobacterium tuberculosis* | *Tropheryma whipplei* |  | 131 | 0,81951 | 0,77923 |
| *Chlamydia pneumoniae* | *Legionella pneumophila* |  | 119 | 0,85473 | 0,76039 |
| *Treponema pallidum* | *Staphylococcus aureus* |  | 97 | 0,43771 | 0,75873 |
| *Treponema pallidum* | *Leptospira interrogans* |  | 108 | 0,63775 | 0,74766 |
| *Treponema pallidum* | *Burkholderia_multivorans* |  | 116 | 0,67714 | 0,74684 |
| *Leishmania* | *Homo sapiens* |  | 76 | 0,16706 | 0,74027 |
| *Chlamydia pneumoniae* | *Escherichia coli* |  | 109 | 0,72024 | 0,73912 |
| *Treponema pallidum* | *Schistosoma mansonii* | * | 88 | 0,46295 | 0,71362 |
| *Anaplasma phagocytophilium* | *Tropheryma whipplei* |  | 102 | 0,81087 | 0,71279 |
| *Leptospira_interrogans* | *Mycobacterium_tuberculosis* |  | 106 | 0,71761 | 0,70157 |

**Section 5. Alignment of HSP60 sequences.**

**Supplementary Figure SF4.** An alignment of 34 HSP60 sequences.

Turqoise background: Epitopes crossreactive with HSP60 peptides of Mycobacterial, Chlamydial, *E. coli* and human origin, defined by Perschinka et al ([*2*](#_ENREF_2)).

Gray background: *Porphyromonas gingivalis* peptide related to autoimmunity Jeong et al ([*3*](#_ENREF_3)).

Underlined are the i. nonapeptide from the leader of human HSP60 which binds HLA-E and promotes NK cell killing, Michaelsson et al ([*14*](#_ENREF_14)),

ii. Overstrike: *E. coli* Peptide frequently antigenic in cardiovascular disease (Okada et al, ([*4*](#_ENREF_4)).

Red text indicates homologs of human HSP60 G20c.

Serologically reactive octameric peptides of Orientia tsutsugamushi Sta58, an HSP60 protein, (Lachumanan et al, ([*15*](#_ENREF_15)) are shown. Further details on HSP60 epitopes are given in section 6.

The positions of the apical loops and helices, as defined in *E. coli* GroEL, Braig et al ([*16*](#_ENREF_16)) are also indicated. Alignment positions, referring to the numbers in Figure 7, are shown to the right.

CLUSTAL W (1.83) multiple sequence alignment

50

GroEL_orientia_tsutsugamushi_C --------------------------------MSKQIVHGDQCRKKIIEG

GroEL_rickettsia_bellii_YP_537 -------------------------------MATKLIKHGSKAREQMLEG

GroEL5_neoehrlichia_mikurensis --------------------------------MANVVVTGETLDKSIRDI

GroEL_anaplasma_phagocytophili --------------------------------MSNTVVTGEVLDKSIREV

GroEL_ehrlichia_deer_AB454077 --------------------------------MANVVVTGEQLDKSIREV

GroEL_Wolbachia_YP_198181 --------------------------------MTNVVVSGEQLQEAFREV

GroEL_Borrelia_garinii_YP_0730 --------------------------------MAKDIYFNEDARKSLLSG

GroEL_treponema_pallidum_YP_00 --------------------------------MAKQLLFNEEARKKLLSG

GroEL_Leptospira_interrogans_Y --------------------------------MAKDIEYNETARRKLLEG

GroEL_CH60_CHLPN_chlamydophila -------------------------------MAAKNIKYNEEARKKIHKG

GroEL3_chlamydia_psittaci_AEG8 -------------------------------MAAKNIKYNEDARKKIHKG

GroEL2_salmonella_enterica_typ -------------------------------MAAKDVKFGNDARVKMLRG

GroEL_salmonella_typhi_TY2_U01 -------------------------------MAAKDVKFGNDARVKMLRG

GroEL_E_coli_K12_AAC77103 -------------------------------MAAKDVKFGNDARVKMLRG

GroEL_Yersinia_pestis_NP_99189 -------------------------------MAAKDVKFGNDARIKMLRG

GroEL_haemophilus_influenzae_Y -------------------------------MAAKDVKFGNDARVKMLKG

GroEL_Legionella_YP_126086 --------------------------------MAKELRFGDDARLQMLAG

GroEL_Burkholderia_multivorans -------------------------------MAAKDVKFHDGARSRIVKG

GroEL_Bartonella_henselae_YP_0 -------------------------------MAAKEVKFGREARERLLRG

GroEL_brucella_abortus_ZP_0587 -------------------------------MAAKDVKFGRTAREKMLRG

GroEL_Mycobact_tuberc_CAA17397 --------------------------------MAKTIAYDEEARRGLERG

GroEL_Mycobacterium_leprae_TN_ --------------------------------MAKTIAYDEEARRGLERG

GroEL_tropheryma_whipplei_NP_7 --------------------------------MAKKITFNEDARRGLERG

GroEL_Staph_aureus_MRSA_YP_041 --------------------------------MVKQLKFSEDARQAMLRG

GroEL_Listeria_monocytogenes_A --------------------------------MAKDIKFSEDARRAMLRG

GroEL_streptococcus_pneumoniae --------------------------------MSKEIKFSSDARSAMVRG

GroEL_mycoplasma_penetrans_NP_ --------------------------------MAKEIKFSDSARNKLFNG

HSP60_Leishmania_Strain_Friedl ------------------MLSRTVPRCVKYGSTPKDIRYGMEARNALLAG

HSP60_Schistosoma_mansoni_XP_0 -------MLRAFATLRGT--LAPVRHRVIQRSYAKEVKFGADARSAMLVG

Hsp60_human_mitochondria_NP_95 -------MLRLPTVFRQMRPVSRVLAPHLTRAYAKDVKFGADARALMLQG

HSP60_Plasmodium_falciparum_XP MISTLRGKIFN--NGSNRNKCVSILSNIQKRNISKDIRFGSDARTAMLTG

chaperonin60_Cryptosporidium_p MLLRSGINLYKSVEGSIGLRSAAIRFGMRYISSGKELSFGGKARKEMLKG

Chaperonin60_Entamoeba_histoly ------------------------MLSSSSHYNGKLLSLNIDCRENVLSG

Chaperonin60_Giardia_lamblia_X -----------------------------MLQHYTSVISGEDARSGLLRG

. . .

100

GroEL_orientia_tsutsugamushi_C INVVANAVGITLGPKGRCVAIEQSYG--PPKITKDGVSVAKAIQLKDKSL

GroEL_rickettsia_bellii_YP_537 IDILADAVKVTLGPKGRNVLIEQSFG--APKITKDGVTVAKSIELKDKIR

GroEL5_neoehrlichia_mikurensis IRILEDAVGCTAGPKGLTIAISKPYG--TPEITKDGYKVIKSIKPEEPLA

GroEL_anaplasma_phagocytophili VRILEDAVGCTAGPKGLTVAISKPYG--SPEITKDGYKVMKSIKPEEPLA

GroEL_ehrlichia_deer_AB454077 VRILEDAVGCTAGPKGLTVAIGKSYG--APEITKDGYKVIKSIKPEDPLA

GroEL_Wolbachia_YP_198181 AVMVDSTVAITAGPRGKTVGINKPYG--APEITKDGYKVMKGIKPEKPLH

GroEL_Borrelia_garinii_YP_0730 VEKLSNAVKVTLGPKGRNVLIDKKFG--SPTVTKDGVSVAREIELENPFE

GroEL_treponema_pallidum_YP_00 VEQISSAVKVTLGPKGRNVLLEKGYG--APTVTKDGVSVAKEVELEDPFE

GroEL_Leptospira_interrogans_Y VNKLANAVKVTLGPKGRNVVIDKKFG--APTITKDGVTVAKEIELEDPLE

GroEL_CH60_CHLPN_chlamydophila VKTLAEAVKVTLGPKGRHVVIDKSFG--SPQVTKDGVTVAKEIELEDKHE

GroEL3_chlamydia_psittaci_AEG8 VKTLAEAVKVTLGPKGRHVVIDKSFG--SPQVTKDGVTVAKEIELEDKHE

GroEL2_salmonella_enterica_typ VNVLADAVKVTLGPKGRNVVLDKSFG--APTITKDGVSVAREIELEDKFE

GroEL_salmonella_typhi_TY2_U01 VNVLADAVKVTLGPKGRNVVLDKSFG--APTITKDGVSVAREIELEDKFE

GroEL_E_coli_K12_AAC77103 VNVLADAVKVTLGPKGRNVVLDKSFG--APTITKDGVSVAREIELEDKFE

GroEL_Yersinia_pestis_NP_99189 VNILADAVKVTLGPKGRNVVLDKSFG--SPTITKDGVSVAREIELEDKFE

GroEL_haemophilus_influenzae_Y VNVLADAVKVTLGPKGRNVILDKSFG--APTITKDGVSVAREIELEDKFE

GroEL_Legionella_YP_126086 VNALADAVQVTMGPRGRNVVLEKSYG--APTVTKDGVSVAKEIEFEHRFM

GroEL_Burkholderia_multivorans VNVLADAVKVTLGPKGRNVLIERSFG--APTITKDGVSVAKEIELKDRFE

GroEL_Bartonella_henselae_YP_0 VDILANAVKVTLGPKGRNVVIDKSFG--APRITKDGVSVAKEIELEDKFE

GroEL_brucella_abortus_ZP_0587 VDILADAVKVTLGPKGRNVVIEKSFG--APRITKDGVSVAKEVELEDKFE

GroEL_Mycobact_tuberc_CAA17397 LNALADAVKVTLGPKGRNVVLEKKWG--APTITNDGVSIAKEIELEDPYE

GroEL_Mycobacterium_leprae_TN_ LNSLADAVKVTLGPKGRNVVLEKKWG--APTITNDGVSIAKEIELEDPYE

GroEL_tropheryma_whipplei_NP_7 LNTLADTVKVTLGPRGRNVVLEKKWG--APVITNDGVTIAKEIELDDPYE

GroEL_Staph_aureus_MRSA_YP_041 VDQLANAVKVTIGPKGRNVVLDKEFT--APLITNDGVTIAKEIELEDPYE

GroEL_Listeria_monocytogenes_A VDQLANAVKVTLGPKGRNVVLEKKFG--SPLITNDGVTIAKEIELEDPFE

GroEL_streptococcus_pneumoniae VDILADTVKVTLGPKGRNVVLEKSFG--SPLITNDGVTIAKEIELEDHFE

GroEL_mycoplasma_penetrans_NP_ VQQLFDAVKVTMGPRGRNVLIQKSYG--APVITKDGVSVAKEVDLTNPIE

HSP60_Leishmania_Strain_Friedl VENLVKAVGVTLGPKGRNVILEMPYA--CPKITKDGVTVAKSIEFEDSFE

HSP60_Schistosoma_mansoni_XP_0 VDILADAVAVTMGPKGRNVIIESSWK--SPKITKDGVTVAKGIELKDKFQ

Hsp60_human_mitochondria_NP_95 VDLLADAVAVTMGPKGRTVIIEQSWG--SPKVTKDGVTVAKSIDLKDKYK

HSP60_Plasmodium_falciparum_XP CNKLADAVSVTLGPKGRNVIIEQSFG--SPKITKDGVTVAKSIEFNNKLA

chaperonin60_Cryptosporidium_p ANDLADAVGVTLGPRGRNVVIEQGFGE-APKITKDGVTVAKAIQFGKGSV

Chaperonin60_Entamoeba_histoly IKKVADAVSVTLGPKGRTVIIDQPYG--NARVTKDGVSVAKALTFSDNTL

Chaperonin60_Giardia_lamblia_X IKTIADVVATTLGPRGRAVILADGSASGTTKVTKDGVSVARAINLSG-LE

: ..* * **:* : : . :*:.* .: : :

150

GroEL_orientia_tsutsugamushi_C NVGAQFVISVASKTADVAGDGTTTATVIADAAVRELNKAEVAGIDIQEVR

GroEL_rickettsia_bellii_YP_537 NAGAQLLKSAATKAAEVAGDGTTTATVLARALAREGNKLVAAGYNPMDLK

GroEL5_neoehrlichia_mikurensis QAIANIIAQSASQCNDKVGDGTTTCSILTAKVIEEVSKAKAAGADIISIK

GroEL_anaplasma_phagocytophili AAIASIITQSASQCNDKVGDGTTTCSILTAKVIEEVSKAKAAGSDIVSIK

GroEL_ehrlichia_deer_AB454077 LAIANIITQSASQCNDKVGDGTTTCSILTAKVIEEVSKAKAAGADIVCIK

GroEL_Wolbachia_YP_198181 AAITSIFAQSCFQCNDKVGDGTTTCSILTSNMIMEALKSIAAGNDRVSIK

GroEL_Borrelia_garinii_YP_0730 NMGAQLLKEVAIKTNDVAGDGTTTATVLAYAIAREGLKNVSSGINPIGIK

GroEL_treponema_pallidum_YP_00 NMGAQLLKEVATKTNDVAGDGTTTATVLAYSMVREGLKAVAAGMTPLELK

GroEL_Leptospira_interrogans_Y NMGAQMVKEVSTKTNDVAGDGTTTATILAQSIINEGLKNVTAGANPMSLK

GroEL_CH60_CHLPN_chlamydophila NMGAQMVKEVASKTADKAGDGTTTATVLAEAIYSEGLRNVTAGANPMDLK

GroEL3_chlamydia_psittaci_AEG8 NMGAQMVKEVASKTADKAGDGTTTATVLAEAIYSEGLRNVTAGANPMDLK

GroEL2_salmonella_enterica_typ NMGAQMVKEVASKANDAAGDGTTTATVLAQSIITEGLKAVAAGMNPMDLK

GroEL_salmonella_typhi_TY2_U01 NMGAQMVKEVASKANDAAGDGTTTATVLAQSIITEGLKAVAAGMNPMDLK

GroEL_E_coli_K12_AAC77103 NMGAQMVKEVASKANDAAGDGTTTATVLAQAIITEGLKAVAAGMNPMDLK

GroEL_Yersinia_pestis_NP_99189 NMGAQMVKEVASKANDAAGDGTTTATVLAQSIITEGLKAVAAGMNPMDLK

GroEL_haemophilus_influenzae_Y NMGAQMVKEVASKANDAAGDGTTTATVLAQAIVNEGLKAVAAGMNPMDLK

GroEL_Legionella_YP_126086 NMGAQMVKEVASKTSDTAGDGTTTATVLARSILVEGHKAVAAGMNPMDLK

GroEL_Burkholderia_multivorans NMGAQVVKQVASKTADVAGDGTTTATVLAQAIVQEGMKHVAAGINPMDLK

GroEL_Bartonella_henselae_YP_0 NMGAQMLREVASKTNDIAGDGTTTATVLGQAIVQEGVKAVAAGMNPMDLK

GroEL_brucella_abortus_ZP_0587 NMGAQMLREVASKTNDTAGDGTTTATVLGQAIVQEGAKAVAAGMNPMDLK

GroEL_Mycobact_tuberc_CAA17397 KIGAELVKEVAKKTDDVAGDGTTTATVLAQALVREGLRNVAAGANPLGLK

GroEL_Mycobacterium_leprae_TN_ KIGAELVKEVAKKTDDVAGDGTTTATVLAQALVKEGLRNVAAGANPLGLK

GroEL_tropheryma_whipplei_NP_7 KIGAELVKEVAKKTDDVAGDGTTTSVVLAQAMVREGLKNVAAGADPISLR

GroEL_Staph_aureus_MRSA_YP_041 NMGAKLVQEVANKTNEIAGDGTTTATVLAQAMIQEGLKNVTSGANPVGLR

GroEL_Listeria_monocytogenes_A NMGAKLVSEVASKTNDVAGDGTTTATVLAQAMIQEGLKNVTAGANPVGVR

GroEL_streptococcus_pneumoniae NMGAKLVSEVASKTNDIAGDGTTTATVLTQAIVREGIKNVTAGANPIGIR

GroEL_mycoplasma_penetrans_NP_ NMGAQLVKDVASKTADEAGDGTTTATVLAYGVFKEGLRNVISGANPIEIK

HSP60_Leishmania_Strain_Friedl NLGANLVRQVAGLTNDNAGDGTTTATVLSGAIFKEGFRSVASGTNPMDLK

HSP60_Schistosoma_mansoni_XP_0 NIGAKLVQDVANNTNEEAGDGTTTATVLARAIAKEGFEKISKGANPIEFR

Hsp60_human_mitochondria_NP_95 NIGAKLVQDVANNTNEEAGDGTTTATVLARSIAKEGFEKISKGANPVEIR

HSP60_Plasmodium_falciparum_XP NLGAQMVKQVAANTNDKAGDGTTTATILARSIFQQGCKAVDSGMNPMDLL

chaperonin60_Cryptosporidium_p NLGAQLLKNVAISTNEEAGDGTTTATVLARAIFKSGCEKVDAGLNPMDLL

Chaperonin60_Entamoeba_histoly NVGGKIAKEVASKVNDRSGDGTTTATCLLRKVACEGVQAINTGLSGTDLL

Chaperonin60_Giardia_lamblia_X GVGADLIKDASLRTNTMAGDGTTTSLILSGKLVNEMNKYALSGLGNLQLL

.. . . ***:**. : . . * .

200

GroEL_orientia_tsutsugamushi_C KGAEKAVEAVIADVRKNSS--PVKNEEEIAQVATVSSNGDREIGEKIANA

GroEL_rickettsia_bellii_YP_537 RGMDLAVNTVLEEVKKASK--KIDSQEEIAQVGTISSNGDKEIGEKIAKA

GroEL5_neoehrlichia_mikurensis NGILKAKELVLESLLSMKR--DVSSEDEIAQVATISANGDKNIGSKIAQC

GroEL_anaplasma_phagocytophili NGILKAKEAVLTALMSMRR--EVE-EDEIAQVATLSANGDKNIGSKIAQC

GroEL_ehrlichia_deer_AB454077 EGVLKAKEAVLEALMSMKR--EVLSEEEIAQVATISANGDKNIGSKIAQC

GroEL_Wolbachia_YP_198181 NGMQKAKDAVLEGITSMSRTIPLEKMDEVAQVAIISANGDKDIGNSIADA

GroEL_Borrelia_garinii_YP_0730 KGIDHAVNLAAEKIRQSAK--KITTKEEIAQVASISANNDSYIGEKIAEA

GroEL_treponema_pallidum_YP_00 RGMDKAVAIAVDDIKQNSK--GIKSNEEVAHVASVSANNDKEIGRILASA

GroEL_Leptospira_interrogans_Y KGIDKAVTAAVESIQKRAV--KIENKKDIANVASISANNDNTIGNLIADA

GroEL_CH60_CHLPN_chlamydophila RGIDKAVKVVVDELKKISK--PVQHHKEIAQVATISANNDSEIGNLIAEA

GroEL3_chlamydia_psittaci_AEG8 RGIDKAVKVVVDQIKKISK--PVQHHKEIAQVATISANNDSEIGNLIAEA

GroEL2_salmonella_enterica_typ RGIDKAVAAAVEELKALSV--PCSDSKAIAQVGTISANSDETVGKLIAEA

GroEL_salmonella_typhi_TY2_U01 RGIDKAVAAAVEELKALSV--PCSDSKAIAQVGTISANSDETVGKLIAEA

GroEL_E_coli_K12_AAC77103 RGIDKAVTAAVEELKALSV--PCSDSKAIAQVGTISANSDETVGKLIAEA

GroEL_Yersinia_pestis_NP_99189 RGIDKAVIAAVEELKKLSV--PCSDSKAIAQVGTISANSDSTVGELIAQA

GroEL_haemophilus_influenzae_Y RGIDKAVSAVVSELKNLSK--PCETAKEIEQVGTISANSDSIVGQLISQA

GroEL_Legionella_YP_126086 RGIDKAVLAVTKKLQAMSK--PCKDSKAIAQVGTISANSDEAIGAIIAEA

GroEL_Burkholderia_multivorans RGIDKAVGAVLDELRKLSR--PIATNKEIAQVGAISANSDEAIGKIIADA

GroEL_Bartonella_henselae_YP_0 RGIDAAVDEVVANLFKKAK--KIQTSAEIAQVGTISANGAAEIGKMIADA

GroEL_brucella_abortus_ZP_0587 RGIDLAVNEVVAELLKKAK--KINTSEEVAQVGTISANGEAEIGKMIAEA

GroEL_Mycobact_tuberc_CAA17397 RGIEKAVEKVTETLLKGAK--EVETKEQIAATAAISA-GDQSIGDLIAEA

GroEL_Mycobacterium_leprae_TN_ RGIEKAVDKVTETLLKDAK--EVETKEQIAATAAISA-GDQSIGDLIAEA

GroEL_tropheryma_whipplei_NP_7 RGIEKSVAAVSKALLTSAK--EVETEAEIAACASISA-GDPQIGDIIAQA

GroEL_Staph_aureus_MRSA_YP_041 QGIDKAVKVAVEALHENSQ--KVENKNEIAQVGAISA-ADEEIGRYISEA

GroEL_Listeria_monocytogenes_A RGIEKAVATAIEELKAISK--PIESKESIAQVAAISS-GDEEVGKLIAEA

GroEL_streptococcus_pneumoniae RGIETAVAAAVEALKNNAI--PVANKEAIAQVAAVSS-RSEKVGEYISEA

GroEL_mycoplasma_penetrans_NP_ RGMDKTVNAIVNELNKSSK--KIARKDEIIQVATISANSDKKIGELIANA

HSP60_Leishmania_Strain_Friedl RGIDLACREVLISLAEQSR--PVTSKSEITQVAMISANMDQEIGSLIGDA

HSP60_Schistosoma_mansoni_XP_0 RGVMSAVDAVVKELKSLSK--PISTPEEIAKSQQYQPTVTKRLA-----I

Hsp60_human_mitochondria_NP_95 RGVMLAVDAVIAELKKQSK--PVTTP~~EEIAQVATISANGDKEIGNII~~SDA

HSP60_Plasmodium_falciparum_XP RGINKGVEKVLEYLNSIKK--DVTTTEEIFNVASISANGDKNIGQLIADT

chaperonin60_Cryptosporidium_p RGIKLGVEHVVNELDLLSQ--PVKSHDDILNVATISANGDSIVGSLIAQA

Chaperonin60_Entamoeba_histoly KGISIAKDIVLKEITKQSK---PTLKEDIISVARVSANNDEKIGEMVGDI

Chaperonin60_Giardia_lamblia_X QALNSAGVDCLQSLRKQSR--AIESNKMLYSVATIAANNDPKIGKVVSDA

.. : : . :.

1^st^ apical loop 250

GroEL_orientia_tsutsugamushi_C MKQVGQEGVITVEDSKNFN-FEVEVVKGMRFDRGYISQYFATNREKMITE

GroEL_rickettsia_bellii_YP_537 MEEVGKEGVITVEEAKNFS-FDVEVVKGMMFDRGYLSPYFVTNSEKMVAE

GroEL5_neoehrlichia_mikurensis VKEVGKDGVITVEESKGFKELEVEKTDGMQFDRGYLSPYFVTNAEKMLIE

GroEL_anaplasma_phagocytophili VKEVGKDGVITVEESKGFKDLEVEKTDGMQFDRGYLSPYFVTNAEKMLVE

GroEL_ehrlichia_deer_AB454077 VQEVGKDGVITVEESKGFKELDVEKTDGMQFDRGYLSPYFVTNSEKMLVE

GroEL_Wolbachia_YP_198181 VKKVGKEGVITVEESKGSKELEVELTTGMQFDRGYLSPYFITSNEKMIVE

GroEL_Borrelia_garinii_YP_0730 MDKVGKDGVITVEESKTFD-TTISYVEGMQFDRGYLSPYFSTNKENMSVS

GroEL_treponema_pallidum_YP_00 IEKVGNDGVIDVDEAQTME-TVTEFVEGMQFDRGYISSYFVTDRDRMETV

GroEL_Leptospira_interrogans_Y MDKVGKDGVITVEEAKSIE-TTLDVVEGMQFDRGYISPYMVTDAESMVAT

GroEL_CH60_CHLPN_chlamydophila MEKVGKNGSITVEEAKGFE-TVLDVVEGMNFNRGYLSSYFSTNPETQECV

GroEL3_chlamydia_psittaci_AEG8 MEKVGKNGSITVEEAKGFE-TVLDVVEGMNFNRGYLSSYFSTNPETQECV

GroEL2_salmonella_enterica_typ MDKVGKEGVITVEDGTGLQ-DELDVVEGMQFDRGYLSPYFINKPETGAVE

GroEL_salmonella_typhi_TY2_U01 MDKVGKEGVITVEDGTGLQ-DELDVVEGMQFDRGYLSPYFINKPETGAVE

GroEL_E_coli_K12_AAC77103 MDKVGKEGVITVEDGTGLQ-DELDVVEGMQFDRGYLSPYFINKPETGAVE

GroEL_Yersinia_pestis_NP_99189 MEKVGKEGVITVEEGSGLQ-DELDVVEGMQFDRGYLSPYFINKPETGSIE

GroEL_haemophilus_influenzae_Y MEKVGKEGVITVEDGTGLE-DELDVVEGMQFDRGYLSPYFINKPETATVE

GroEL_Legionella_YP_126086 MEKVGKEGVITVEDGNGLE-NELSVVEGMQFDRGYISPYFINNQQNMSCE

GroEL_Burkholderia_multivorans MERVGKEGVITVEDGKSLE-NELEVVEGMQFDRGYVSPYFINDPEKQAAY

GroEL_Bartonella_henselae_YP_0 MEKVGNEGVITVEEAKTAE-TELEVVEGMQFDRGYLSPYFVTNAEKMVAD

GroEL_brucella_abortus_ZP_0587 MQKVGNEGVITVEEAKTAE-TELEVVEGMQFDRGYLSPYFVTNPEKMVAD

GroEL_Mycobact_tuberc_CAA17397 MDKVGNEGVITVEESNTFG-LQLELTEGMRFDKGYISGYFVTDPERQEAV

GroEL_Mycobacterium_leprae_TN_ MDKVGNEGVITVEESNTFG-LQLELTEGMRFDKGYISGYFVTDAERQEAV

GroEL_tropheryma_whipplei_NP_7 LEKVGKEGVVTVEESNTFG-TELEITEGMRFDKGYLSAYFVTDAERQETV

GroEL_Staph_aureus_MRSA_YP_041 MEKVGNDGVITIEESNGLN-TELEVVEGMQFDRGYQSPYMVTDSDKMVAE

GroEL_Listeria_monocytogenes_A MERVGNDGVITIEESKGFA-TELDVVEGMQFDRGYTSPYMVTDSDKMEAV

GroEL_streptococcus_pneumoniae MEKVGKDGVITIEESRGME-TELEVVEGMQFDRGYLSQYMVTDSEKMVAD

GroEL_mycoplasma_penetrans_NP_ MEKVGSDGVITVEEAKGIN-DELTVVEGMQFDRGYISPYFVTDTNKMIAK

HSP60_Leishmania_Strain_Friedl MQQVGKDGVITTQEGRSLN-TELELVEGMSFERGYTSPYFVTNTKAQRCE

HSP60_Schistosoma_mansoni_XP_0 MKKVGNDGTITVKDGKTLH-DELEFIEGMKFDRGYISPYFLNTEKGARCE

Hsp60_human_mitochondria_NP_95 MKKVGRKGVITVKDGKTLN-DELEIIEGMKFDRGYISPYFINTSKGQKCE

HSP60_Plasmodium_falciparum_XP MKKVGKEGTITVTEGKTLQ-HELEIVEGIKFDRGYISPYFINNSKDQKVE

chaperonin60_Cryptosporidium_p YSKVGRHGTINIEEGNTTQ-SELEIVEGLKLDKGYISPYFITNQKYQKVE

Chaperonin60_Entamoeba_histoly FGKIGRDGAVDIETGKGTK-DIVNIVEGMVLDQGFLSRYFTTDEKNTKVD

Chaperonin60_Giardia_lamblia_X FAAVGREGTITVEDG-YTDIDTLNVTDGCSIPSGFLSPYFALGGSR-YLE

:* .* : . . : *: * *: .

apical H helix, apical I helix 300

HHHHHHHHHH IIIIII

GroEL_orientia_tsutsugamushi_C FENPYILLLDQKVSTV-QPLVPVLEAVAHTGK-PLVLIADDVDGEALTAL

GroEL_rickettsia_bellii_YP_537 LENPYILLFEKKLSNL-QPMLPILEAVVQSQR-PLLIIAEDVEGEALATL

GroEL5_neoehrlichia_mikurensis FENPYILLTEKKLNII-QPILPILENIARSGR-PLLIIAEDVEGEALSTL

GroEL_anaplasma_phagocytophili FENPYIFLTEKKINLV-QSILPILENVARSGR-PLLIIAEDVEGEALSTL

GroEL_ehrlichia_deer_AB454077 FENPYILLTEKKLNII-QPILPILENVARSGR-PLLIIAEDVEGEALSTL

GroEL_Wolbachia_YP_198181 FDDPYLLITEKKLSII-QPLLPILEAVVKSGK-PLLIIAEDIEGEALSTL

GroEL_Borrelia_garinii_YP_0730 FDDAFILIYEKKISSI-KELLPVLEKVLGTNK-PLLIIAEDIEGDALAAL

GroEL_treponema_pallidum_YP_00 YENPYILIYDKSISTM-KDLLPLLEKIAQTGR-PLLIIAEDVEGEALATL

GroEL_Leptospira_interrogans_Y LNDPFILIYDKKISSM-KDLIHILEKVAQAGK-PLVIISEEVEGEALATI

GroEL_CH60_CHLPN_chlamydophila LEDALILIYDKKISGI-KDFLPVLQQVAESGR-PLLIIAEEIEGEALATL

GroEL3_chlamydia_psittaci_AEG8 LEEALVLIYDKKISGI-KDFLPVLQQVAESGR-PLLIIAEDIEGEALATL

GroEL2_salmonella_enterica_typ LESPFILLADKKI*SNI-REMLPVLEAVAKAG*K-PLLIIAEDVEG*EALATL*

GroEL_salmonella_typhi_TY2_U01 LESPFILLADKKISNI-REMLPVLEAVAKAGK-PLLIIAEDVEGEALATL

GroEL_E_coli_K12_AAC77103 LESPFILLADKKISNI-REMLPVLEAVAKAGK-PLLIIAEDVEGEALATL

GroEL_Yersinia_pestis_NP_99189 LESPFILLADKKISNI-REMLPVLEAVAKAGK-PLLIIAEDVEGEALATL

GroEL_haemophilus_influenzae_Y LDNPYLLLVDKKISNI-RELLPVLEGVAKAGK-PLLIIAEDVEGEALATL

GroEL_Legionella_YP_126086 LEHPFILLVDKKVSSI-REMLSVLEGVAKSGR-PLLIIAEDVEGEALATL

GroEL_Burkholderia_multivorans LDDPLILLHDKKISSI-RDLLPILEAASKAGK-PLLIVAEDVDGEALATL

GroEL_Bartonella_henselae_YP_0 LDDPYILIHEKKLSNL-QSLLPVLEAVVQSGK-PLLIIAEDVEGEALATL

GroEL_brucella_abortus_ZP_0587 LEDAYILLHEKKLSNL-QALLPVLEAVVQTSK-PLLIIAEDVEGEALATL

GroEL_Mycobact_tuberc_CAA17397 LEDPYILLVSSKVSTV-KDLLPLLEKVIGAGK-PLLIIAEDVEGEALSTL

GroEL_Mycobacterium_leprae_TN_ LEEPYILLVSSKVSTV-KDLLPLLEKVIQAGK-SLLIIAEDVEGEALSTL

GroEL_tropheryma_whipplei_NP_7 FENPYILICDSKISSV-KDLLPVVDKVIQSGK-QLLIIAEDVDGEALATL

GroEL_Staph_aureus_MRSA_YP_041 LERPYILVTDKKISSF-QDILPLLEQVVQSNR-PILIVADEVEGDALTNI

GroEL_Listeria_monocytogenes_A LEKPYILITDKKINNI-QEILPVLEQVVQQGR-PMLIIAEDVEGEAQATL

GroEL_streptococcus_pneumoniae LENPYILITDKKISNI-QEILPLLESILQSNR-PLLIIADDVDGEALPTL

GroEL_mycoplasma_penetrans_NP_ LENPYILITDKKVSSI-KDILPILEEIMKTGR-PLLIIADDVDGEALTTL

HSP60_Leishmania_Strain_Friedl LENALVYVANRKLTSV-AHILPALNYAIQQKR-PLLVIAEDVEGEAMHTF

HSP60_Schistosoma_mansoni_XP_0 FQDAFVLFSEKKINSI-QTLLPALELCHQQKR-PLLIIAEDVEGEALTAL

Hsp60_human_mitochondria_NP_95 FQDAYVLLSEKKISSI-QSIVPALEIANAHRK-PLVIIAEDVDGEALSTL

HSP60_Plasmodium_falciparum_XP LDKPYILIHEKKISTV-KSLLPVLEHVLQNQS-SLLVIAEDVDSDALATL

chaperonin60_Cryptosporidium_p LENPYILISQGKISSL-KSILPILEFCISSRS-PLLIIAEEIEGEALTAL

Chaperonin60_Entamoeba_histoly IRNTDVIVCDYKLSSS-QSVVPLLELCLKRKR-PLVVISDTIDGDALTTL

Chaperonin60_Giardia_lamblia_X LTNPLVVITDTVLSSA-APLVSILERCVKEKR-PLLIIASDVTGDALSTL

. : . . :. .: :: :::::. . : :

2^nd^ apical loop 350

IIIIIII

GroEL_orientia_tsutsugamushi_C ILNNLKGSIKVVAVKAPGFGDRKKEMLEDIAILTNG-EVITEQLGIKLEK

GroEL_rickettsia_bellii_YP_537 VVNRLRGGLKVAAVKAPGFGDRRKAMMEDIAILTNG-ELITEDLGMKLEN

GroEL5_neoehrlichia_mikurensis VLNKLRGGLHVAAVKAPGFGDRRKDMLGDIAILTGAKYVINDELAVKMED

GroEL_anaplasma_phagocytophili VLNKLRGGLQVAAVKAPGFGDRRKDMLGDIAVIVGAKYVVNDELAVKMED

GroEL_ehrlichia_deer_AB454077 VLNKLRGGLHVAAVKAPGFGDRRKDMLGDIAILTGAKHVISDDLAIKMED

GroEL_Wolbachia_YP_198181 VINKLRGGLKVTAVKAPGFGDRRKEMLEDIAALTGAKYVIKDELGIKMED

GroEL_Borrelia_garinii_YP_0730 VLNSVRGALKVCAIKSPGFGDRRKAMLEDIAVLTGG-VLISEELGITLET

GroEL_treponema_pallidum_YP_00 VVNSLRGTLKTCAVKAPGFGDRRKEMLEDIAILSGG-QVISEDLGLKLES

GroEL_Leptospira_interrogans_Y VVNTLRKTISCVAVKAPGFGDRRKSMLEDIAILTGG-QVISEDLGMKLEN

GroEL_CH60_CHLPN_chlamydophila VVNRLRAGFRVCAVKAPGFGDRRKAMLEDIAILTGG-QLVSEELGMKLEN

GroEL3_chlamydia_psittaci_AEG8 VVNRLRAGFRVCAVKAPGFGDRRKAMLEDIAILTGG-QLISEELGMKLEN

GroEL2_salmonella_enterica_typ VVNTMRGIVKVAAVKAPGFGDRRKAMLQDIATLTGG-TVISEEIGMELEK

GroEL_salmonella_typhi_TY2_U01 VVNTMRGIVKVAAVKAPGFGDRRKAMLQDIATLTGG-TVISEEIGMELEK

GroEL_E_coli_K12_AAC77103 *VVNTMRGIVKVAA*VKAPGFGDRRKAMLQDIATLTGG-TVISEEIGMELEK

GroEL_Yersinia_pestis_NP_99189 VVNTMRGIVKVAAVKAPGFGDRRKAMLQDIATLTAG-TVISEEIGLELEK

GroEL_haemophilus_influenzae_Y VVNTMRGIVKVAAVKAPGFGDRRKAMLQDIAILTAG-TVISEEIGMELEK

GroEL_Legionella_YP_126086 VVNNMRGIVKVCAVKAPGFGDRRKAMLQDIAILTKG-QVISEEIGKSLEG

GroEL_Burkholderia_multivorans VVNAMRGILKVAAVKAPGFGDRRKAMLEDIAILTGA-TVISEETGKQLEK

GroEL_Bartonella_henselae_YP_0 VVNKLRGGLKIAAVKAPGFGDRRKAMLEDIAILTSG-QVISEDVGIKLEN

GroEL_brucella_abortus_ZP_0587 VVNKLRGGLKIAAVKAPGFGDRRKAMLEDIAILTGG-QVISEDLGIKLES

GroEL_Mycobact_tuberc_CAA17397 VVNKIRGTFKSVAVKAPGFGDRRKAMLQDMAILTGG-QVISEEVGLTLEN

GroEL_Mycobacterium_leprae_TN_ VVNKIRGTFKSVAVKAPGFGDRRKAMLQDMAILTGA-QVISEEVGLTLEN

GroEL_tropheryma_whipplei_NP_7 VVNKIRGIFKSVAVKAPGFGDRRKMMLQDIAVLTGG-QVISEEVGLKLEN

GroEL_Staph_aureus_MRSA_YP_041 VLNRMRGTFTAVAVKAPGFGDRRKAMLEDLAILTGA-QVITDDLGLDLKD

GroEL_Listeria_monocytogenes_A VLNKLRGTFNVVAVKAPGFGDRRKAMLEDIAILTGG-QVITEDLGLELKT

GroEL_streptococcus_pneumoniae VLNKIRGTFNVVAVKAPGFGDRRKAMLEDIAILTGG-TVITEDLGLELKD

GroEL_mycoplasma_penetrans_NP_ VVNKMRGVFNVVAVKAPEFGDKRKQVLEDIAILTGG-SFVTDDLGISFDK

HSP60_Leishmania_Strain_Friedl LYNKIQGRISGCAVKAPGFGDMRINQLQDIAVFTGS-QMISEDLGLSLDQ

HSP60_Schistosoma_mansoni_XP_0 VLNRLKLGLQVCAVKAPGFGDNRKNTLKDMAVATGGIVFGDEADMYKLED

Hsp60_human_mitochondria_NP_95 VLNRLKVGLQVVAVKAPGFGDNRKNQLKDMAIATGGAVFGEEGLTLNLED

HSP60_Plasmodium_falciparum_XP IVNKLRLGLKICAVKAPGFGEHRKALIHDIAVMTGAKVITEETG-LKLDD

chaperonin60_Cryptosporidium_p ILNKLQLNLKVCAVKAPGFGDHRKQILEDISVSVGAKIIQEEFSNAKLDQ

Chaperonin60_Entamoeba_histoly VLNKLRG-LPIAAVRAPGFGETRKGILHDIGIITGA-TVISNEAGKKIEE

Chaperonin60_Giardia_lamblia_X AINTLKGTVRCCAVRAPGYGDVKKGVLEDLAAVVGIPTYISDELHTASAP

* :: . *:::* :*: : : *:. :

400

GroEL_orientia_tsutsugamushi_C VN--DTSK-LGTANRVIVTKDHTTIVHDKNNSDIEKKVNSRCEQIREAIK

GroEL_rickettsia_bellii_YP_537 VS--LKS--LGHAKRVTISKENTVIVDGSGD---KKNIEERVLQIKSHIA

GroEL5_neoehrlichia_mikurensis LT--LDD--LGTAKNIRITKDTTTLIGSVDSNS--SNVQSRINQIKMQID

GroEL_anaplasma_phagocytophili IA--LSD--LGTAKSVRITKDATTIIGSVDSSS--ESIASRTNQIKAQIE

GroEL_ehrlichia_deer_AB454077 LT--LAE--LGTAKNIRITKDTTTIIGSVDNSS--ANVQNRINQIKMQIE

GroEL_Wolbachia_YP_198181 LT--LED--LGTAKNVKVTKDNTTIVSGS-SDS--DRVKARVEQIKSQIE

GroEL_Borrelia_garinii_YP_0730 VE--IEQ--LGQAKTIKVDKDNTTIIN-TGNKE---QIKERSELIKKQIE

GroEL_treponema_pallidum_YP_00 AD--IAL--LGQAKSVKVDKENTTIIDGSGKSK---DIKDRIEQIKKQIE

GroEL_Leptospira_interrogans_Y TT--LQM--LGRANKVTVDKENTTIIEGKGQTK---EIQGRIGQIKKQIE

GroEL_CH60_CHLPN_chlamydophila TT--LAM--LGKAKKVIVTKEDTTIVEGLGNKP---DIQARCDNIKKQIE

GroEL3_chlamydia_psittaci_AEG8 TT--LSM--LGKAKKVIVSKEDTTIVEGLGNKE---DIEARCENIKKQIE

GroEL2_salmonella_enterica_typ AT--LED--LGQAKRVVINKDTTTIIDGVGEEA---AIQGRVAQIRQQIE

GroEL_salmonella_typhi_TY2_U01 AT--LED--LGQAKRVVINKDTTTIIDGVGEEA---AIQGRVAQIRQQIE

GroEL_E_coli_K12_AAC77103 AT--LED--LGQAKRVVINKDTTTIIDGVGEEA---AIQGRVAQIRQQIE

GroEL_Yersinia_pestis_NP_99189 TT--LED--LGQAKRVVINKDTTIIIDGVGDEA---AIQGRVAQIRQQIE

GroEL_haemophilus_influenzae_Y AT--LED--LGQAKRVVINKDNTTIIDGIGDEA---QIKGRVAQIRQQIE

GroEL_Legionella_YP_126086 AT--LED--LGSAKRIVVTKENTTIIDGEGKAT---EINARITQIRAQME

GroEL_Burkholderia_multivorans AT--LED--LGRAKRVEVRKDDTIIIDGAGDPA---RIDARVKAIRVQID

GroEL_Bartonella_henselae_YP_0 VT--LDM--LGRAKKVNISKENTTIIDGAGQKS---EINARVNQIKVQIE

GroEL_brucella_abortus_ZP_0587 VT--LDM--LGRAKKVSISKENTTIVDGAGQKA---EIDARVGQIKQQIE

GroEL_Mycobact_tuberc_CAA17397 AD--LSL--LGKARKVVVTKDETTIVEGAGDTD---AIAGRVAQIRQEIE

GroEL_Mycobacterium_leprae_TN_ TD--LSL--LGKARKVVMTKDETTIVEGAGDTD---AIAGRVAQIRTEIE

GroEL_tropheryma_whipplei_NP_7 AT--LDL--LGCARKVVVSKDETTIVDGAGSSD---QIAGRVSQIRKELE

GroEL_Staph_aureus_MRSA_YP_041 AT--IDM--LGTASKVEVTKDNTTVVDGDGDEN---SIDARVSQLKSQIE

GroEL_Listeria_monocytogenes_A AT--VDQ--LGTANKVVVTKDDTTIVEGAGDST---QISARVNQIRAQME

GroEL_streptococcus_pneumoniae AT--IEA--LGQAARVTVDKDSTVIVEGAGNPE---AISHRVAVIKSQIE

GroEL_mycoplasma_penetrans_NP_ VT--LQD--LGQAESVVIDKDNSTIVKGKGLES---QIKERISKIKTAIE

HSP60_Leishmania_Strain_Friedl ND--FSERFLGTCRKVTVSRDECILMEGGGSAI---AVEERVQMIKDMIS

HSP60_Schistosoma_mansoni_XP_0 VQ--LQD--LGRVAEVVVTKDDCLLMRGRGSKT---DVDKRIAQIKEEME

Hsp60_human_mitochondria_NP_95 VQ--PHD--LGKVGEVIVTKDDAMLLKGKGDKA---QIEKRIQEIIEQLD

HSP60_Plasmodium_falciparum_XP P---QVVSYLGKAKSINVTKDSTLIMEGEGKKE---EINERCESIRNAIK

chaperonin60_Cryptosporidium_p MNSNQIQEFLGKCKSISVSKDETIITQGQGSPK---DVKDTISLLKSQIE

Chaperonin60_Entamoeba_histoly VT--EKD--LGKIGHFVSTKDETIITGGAGSKA---EVLARINELKNAKE

Chaperonin60_Giardia_lamblia_X GSAVLSN--IGSCHKAIITPANTVLHFNDDKNCN-SLIRGRVAGLRSLLE

:* : :

450

GroEL_orientia_tsutsugamushi_C DT--TSDYEKEKLQERLAKLRNGVAVLKVGGATEVEQKERKDRVEDALHA

GroEL_rickettsia_bellii_YP_537 ET--TSDYDKEKLQERLAKLSGGVAVLKVGGATEVEVKERKDRVEDALAA

GroEL5_neoehrlichia_mikurensis TS--TSDYDKEKLKERLAKLSGGVAVLKVGGSSEVEVKERKDRVE-----

GroEL_anaplasma_phagocytophili NS--SSDYDKEKLRERLAKLSGGVAVLKVGGSSEVEVKERKDRVEDALHA

GroEL_ehrlichia_deer_AB454077 AS--TSDYDKEKLRERLAKLSGGVAVLKVGGSSEVEVKERKDRVEDALHA

GroEL_Wolbachia_YP_198181 TS--TSDYDKEKLRERLAKLSGGVAVLKVGGVTEVEVKERRDRVEDALHA

GroEL_Borrelia_garinii_YP_0730 DS--TSEYDKEKLQERLAKLVGGVAVINVGAVTEVELKEKKHRVEDALSA

GroEL_treponema_pallidum_YP_00 AS--TSDYDSEKLKERLAKLSGGVAVIKIGAVTEVEMKEKKHRVEDALNA

GroEL_Leptospira_interrogans_Y DT--TSEYDREKLQERLAKLAGGVAVIHVGAATEVEMKEKKARVEDALSA

GroEL_CH60_CHLPN_chlamydophila DS--TSDYDKEKLQERLAKLSGGVAVIRVGAATEIEMKEKKDRVDDAQHA

GroEL3_chlamydia_psittaci_AEG8 DS--TSDYDKEKLQERLAKLSGGVAVIRVGAATEIEMKEKKDRVDDAQHA

GroEL2_salmonella_enterica_typ EA--TSDYDREKLQERVAKLAGGVAVIKVGAATEVEMKEKKARVEDALHA

GroEL_salmonella_typhi_TY2_U01 EA--TSDYDREKLQERVAKLAGGVAVIKVGAATEVEMKEKKARVEDALHA

GroEL_E_coli_K12_AAC77103 EA--TSDYDREKLQERVAKLAGGVAVIKVGAATEVEMKEKKARVEDALHA

GroEL_Yersinia_pestis_NP_99189 DA--TSDYDKEKLQERVAKLAGGVAVIKVGAATEVEMKEKKARVEDALHA

GroEL_haemophilus_influenzae_Y ES--TSDYDKEKLQERVAKLAGGVAVIKVGAATEVEMKEKKDRVDDALHA

GroEL_Legionella_YP_126086 ET--TSDYDREKLQERVAKLAGGVAVIKVGAATEVEMKEKKARVEDALHA

GroEL_Burkholderia_multivorans EA--TSDYDREKLQERVAKLAGGVAVIKVGAATEVEMKEKKDRVDDALHA

GroEL_Bartonella_henselae_YP_0 ET--TSDYDREKLQERLAKLAGGVAVIRVGGATEVEVKEKKDRVDDALNA

GroEL_brucella_abortus_ZP_0587 ET--TSDYDREKLQERLAKLAGGVAVIRVGGATEVEVKEKKDRVDDALNA

GroEL_Mycobact_tuberc_CAA17397 NS--DSDYDREKLQERLAKLAGGVAVIKAGAATEVELKERKHRIEDAVRN

GroEL_Mycobacterium_leprae_TN_ NS--DSDYDREKLQERLAKLAGGVAVIKAGAATEVELKERKHRIEDAVRN

GroEL_tropheryma_whipplei_NP_7 NS--DSDYDREKLQERLAKLSGGVAVIRSGAATEVELKERKHRIEDAVRN

GroEL_Staph_aureus_MRSA_YP_041 ET--ESDFDREKLQERLAKLAGGVAVIKVGAASETELKERKLRIEDALNS

GroEL_Listeria_monocytogenes_A ET--TSEFDREKLQERLAKLAGGVAVVKVGAATETELKERKLRIEDALNS

GroEL_streptococcus_pneumoniae TT--TSEFDREKLQERLAKLSGGVAVIKVGAATETELKEMKLRIEDALNA

GroEL_mycoplasma_penetrans_NP_ MT--DSDYDKDSLRNRLAKLNKGVAVIKVGAVSEVELKEKKDRVDDALSA

HSP60_Leishmania_Strain_Friedl AE--DHEYNRERLVERLAKLSGGVAVIKVGGASEVEINEKKDRIIDALNA

HSP60_Schistosoma_mansoni_XP_0 AS--NSEYEKEKMHERLAKLSNGVAVIKVGGSSEVEVSEKKDRYTDALNA

Hsp60_human_mitochondria_NP_95 VT--TSEYEKEKLNERLAKLSDGVAVLKVGGTSDVEVNEKKDRVTDALNA

HSP60_Plasmodium_falciparum_XP MN--TSDYEKEKLQERLAKITGGVALIKVGGISEVEVNEIKDRIQDALCA

chaperonin60_Cryptosporidium_p ENQKLTDYDKEKLRERLARLTGRVALIKIGGYSDTEISELKDRFIDALNA

Chaperonin60_Entamoeba_histoly VS--DSSYEKEKLEGRIARLTGGVAVISVGGSSEAEVGERKDRIEDAVCA

Chaperonin60_Giardia_lamblia_X SNN-LTNYQRSKLNERIGRLLGKVCTIRIGAKTELEAEEKKDRYIDSLSA

. : . : *:.:: :. : *. :: * * :

500

GroEL_orientia_tsutsugamushi_C TRAAVEEGIVPGGGVALFYASR--VLDS--LKFD----------------

GroEL_rickettsia_bellii_YP_537 TRAAVEEGVVAGGGVTLLHASQ--ALKN--LKVD----------------

GroEL5_neoehrlichia_mikurensis --------------------------------------------------

GroEL_anaplasma_phagocytophili TRAAVEEGVVPGGGAALLYALS--SLDG--LKGK----------------

GroEL_ehrlichia_deer_AB454077 TRACC---------------------------------------------

GroEL_Wolbachia_YP_198181 TRAAIEEGIVPGGGVALLYASS--ALDK--LKGG----------------

GroEL_Borrelia_garinii_YP_0730 TRAAVEEGVVPGGGSTLIEVAM--YLDTIDTSKL----------------

GroEL_treponema_pallidum_YP_00 TRAAIEEGIVAGGGLALIQAAA--ALEKADLSGL----------------

GroEL_Leptospira_interrogans_Y TRAAVEEGIVPGGGLTLLKAQE--AVGSLKLDG-----------------

GroEL_CH60_CHLPN_chlamydophila TIAAVEEGILPGGGTALVRCIP--TLEAFLPMLA----------------

GroEL3_chlamydia_psittaci_AEG8 TLAAVEEGILPGGGTALVRCIP--TLEAFIPVLT----------------

GroEL2_salmonella_enterica_typ TRAAVEEGVVAGGGVALIRVAS--KIAD--LKGQ----------------

GroEL_salmonella_typhi_TY2_U01 TRAAVEEGVVAGGGVALIRVAS--KIAD--LKGQ----------------

GroEL_E_coli_K12_AAC77103 TRAAVEEGVVAGGGVALIRVAS--KLAD--LRGQ----------------

GroEL_Yersinia_pestis_NP_99189 TRAAVEEGVVAGGGVALIRAAH--AIAG--LKGD----------------

GroEL_haemophilus_influenzae_Y TRAAVEEGIVAGGGVALVRAAA--KVAAS-LKGD----------------

GroEL_Legionella_YP_126086 TRAAVEEGIVAGGGVALIRAQK--ALDS--LKGD----------------

GroEL_Burkholderia_multivorans TRAAVEEGIVPGGGVALLRARA--ALAD--IKGA----------------

GroEL_Bartonella_henselae_YP_0 TRAAVEEGIVAGGGTALLRAAN--ALTV---KGS----------------

GroEL_brucella_abortus_ZP_0587 TRAAVEEGIVAGGGTALLRAST--KITA---KGV----------------

GroEL_Mycobact_tuberc_CAA17397 AKAAVEEGIVAGGGVTLLQAAP--TLDELKLEG-----------------

GroEL_Mycobacterium_leprae_TN_ AKAAVEEGIVAGGGVTLLQAAP--ALDKLKLTG-----------------

GroEL_tropheryma_whipplei_NP_7 AKAAVEEGIVAGGGAALLQSGTS-ALKDLQLTS-----------------

GroEL_Staph_aureus_MRSA_YP_041 TRAAVEEGIVAGGGTALVNVYQ--KVSEIEAEG-----------------

GroEL_Listeria_monocytogenes_A TRAAVEEGIVAGGGTALVSIYN--KVAALEAEG-----------------

GroEL_streptococcus_pneumoniae TRAAVEEGIVAGGGTALANVIP--AVATLELTG-----------------

GroEL_mycoplasma_penetrans_NP_ TKAAIEEGIVIGGGAALVHVSKRINVNTLNLIG-----------------

HSP60_Leishmania_Strain_Friedl TRAAVSEGILAGGGTGLLMASLR-LESISKDRRL----------------

HSP60_Schistosoma_mansoni_XP_0 TRAAIEEGIVPGGGTALLRCIP--ILKS--LESK----------------

Hsp60_human_mitochondria_NP_95 TRAAVEEGIVLGGGCALLRCIP--ALDS--LTPA----------------

HSP60_Plasmodium_falciparum_XP TKAAVEEGIVPGGGSALLFASK--ELDSVQTD------------------

chaperonin60_Cryptosporidium_p TKCAIEQGIVPGGGSALLWASR--NLGKLYSQSPPPGKTLTPSQSSSNES

Chaperonin60_Entamoeba_histoly VKAALAEGIVPGGGVALIRAGS--SLDKIRSQNW----------------

Chaperonin60_Giardia_lamblia_X ARAALEGGLLPGGGVAFLRAAQVMERKLAEGKVADP--------------

550

GroEL_orientia_tsutsugamushi_C ----NEDQRVGINIIKKVLEAPVRQIVKNAGGKEDVVVN-ELSK--STDK

GroEL_rickettsia_bellii_YP_537 ----NKDQQAGIELVIEALKDPIKQIVENAGENGGVVVG-KLLE--HKDK

GroEL5_neoehrlichia_mikurensis --------------------------------------------------

GroEL_anaplasma_phagocytophili ----NDDEQWGIDIIRRAACAPIKRIIKNSGSEEAPCVIQHLLK--QNDK

GroEL_ehrlichia_deer_AB454077 --------------------------------------------------

GroEL_Wolbachia_YP_198181 ----SDEEQIGINIIKKVLSAPIKRLVKNAGLES-AVIIDHLTK--QNDK

GroEL_Borrelia_garinii_YP_0730 ----SYEEKQGFEIVKRSLEEPMRQIISNAGFEGSIYIHQIKT----EKK

GroEL_treponema_pallidum_YP_00 ----TPDEAVGFKIVRRALEEPIRQISENAGIDGAVVAEKAK-----EKR

GroEL_Leptospira_interrogans_Y ------DEATGAKIIFRALEEPIRMITSNAGLEGSVIVEHAKA----KKG

GroEL_CH60_CHLPN_chlamydophila ----NEDEAIGTRIILKALTAPLKQIASNAGKEGAIICQQVLA----RSA

GroEL3_chlamydia_psittaci_AEG8 ----NEDEQIGARIVLKALSAPLKQIAANAGKEGAIICQQVLS----RSS

GroEL2_salmonella_enterica_typ ----NEDQNVGIKVALRAMEAPLRQIVLNCGEEPSVVANTVKG----GDG

GroEL_salmonella_typhi_TY2_U01 ----NEDQNVGIKVALRAMEAPLRQIVLNCGEEPSVVANTVKG----GDG

GroEL_E_coli_K12_AAC77103 ----NEDQNVGIKVALRAMEAPLRQIVLNCGEEPSVVANTVKG----GDG

GroEL_Yersinia_pestis_NP_99189 ----NEDQNVGIKVALRAMESPLRQIVVNAGEEASVIANKVKA----GEG

GroEL_haemophilus_influenzae_Y ----NEEQNVGIKLALRAMEAPLRQIVTNAGEEASVVASAVKN----GEG

GroEL_Legionella_YP_126086 ----NDDQNMGINILRRAIESPMRQIVTNAGYEASVVVNKVAE----HKD

GroEL_Burkholderia_multivorans ----NADQDAGIRIVLRALEAPLRVIVSNAGEEPSVVIAKVLE----GKG

GroEL_Bartonella_henselae_YP_0 ----NPDQEAGINIVRRALQAPARQIATNAGEEAAIIVGKVLEN---NAD

GroEL_brucella_abortus_ZP_0587 ----NADQEAGINIVRRAIQAPARQITTNAGEEASVIVGKILEN---TSE

GroEL_Mycobact_tuberc_CAA17397 ------DEATGANIVKVALEAPLKQIAFNSGLEPGVVAEKVRN----LPA

GroEL_Mycobacterium_leprae_TN_ ------DEATGANIVKVALEAPLKQIAFNSGMEPGVVAEKVRN----LSV

GroEL_tropheryma_whipplei_NP_7 ------EEAVGRNIVRSAIEAPLRQISLNAGLEPGVVVGKVSS----LPQ

GroEL_Staph_aureus_MRSA_YP_041 ------DIETGVNIVLKALTAPVRQIAENAGLEGSVIVERLKN----AEP

GroEL_Listeria_monocytogenes_A ------DVETGINIVLRSLEEPVRQIAHNAGLEGSVIVERLKH----EAV

GroEL_streptococcus_pneumoniae ------DEATGRNIVLRALEEPVRQIAHNAGFEGSIVIDRLKN----AEL

GroEL_mycoplasma_penetrans_NP_ ------DEKIGYQIVMSAIMSPISQIVSNAGFDKGVVINEILKA---TNP

HSP60_Leishmania_Strain_Friedl ----PPDIRTGVNIVKKAIGLPARYIANNAGVEGSVVAGKVLAR---KDP

HSP60_Schistosoma_mansoni_XP_0 ----NEDQRTGVQIVLRALSTPCYTIAHNAGVNASVVVEKVMG----MGQ

Hsp60_human_mitochondria_NP_95 ----NEDQKIGIEIIKRTLKIPAMTIAKNAGVEGSLIVEKIMQ----SSS

HSP60_Plasmodium_falciparum_XP ----NYDQRVGVNIIKDACKAPIKQIAENAGHEGSVVAGNILK--EKNS-

chaperonin60_Cryptosporidium_p NPIRNYDMAMGVKIVQDACKVPCHLISSNAGFDGSVIVGELVKVFSKGSK

Chaperonin60_Entamoeba_histoly ------AEKVGIDIVRKVTEEPTRIIARNAGIDGGIVIQKIKEG----TG

Chaperonin60_Giardia_lamblia_X ------VTIAAHKALIAALHEPARIIAESAGASGHVVAEAIKNS---PDN

600

GroEL_orientia_tsutsugamushi_C NRGFDARTMQYVDMIKAGIVDPTKVVRTALQDAFSVASLVIATSAMITD-

GroEL_rickettsia_bellii_YP_537 NFGFNAQDMQYVDMIKAGIIDPAKVVRTALQDAASVASLIITTETLIVD-

GroEL5_neoehrlichia_mikurensis --------------------------------------------------

GroEL_anaplasma_phagocytophili ELIYNVDTMNYANAFTSGVMDPLKVVRIAFDLAVSLAAVFMTLNAVVVD-

GroEL_ehrlichia_deer_AB454077 --------------------------------------------------

GroEL_Wolbachia_YP_198181 ELIYNVEAMNYANAFTAGVIDPAKVVRIAFETAISVASVLITTESMIVD-

GroEL_Borrelia_garinii_YP_0730 GLGFDASSFKWVNMIESGIIDPAKVTRSALQNAASIAGLLLTTECAITD-

GroEL_treponema_pallidum_YP_00 GIGFDASKMEWVDMIKVGIIDPAKVTRSALQNAASVSGLLLTTECAIAA-

GroEL_Leptospira_interrogans_Y NEGFNALTMVWEDMIQAGVVDPAKVVRSALQNAASIGSMILTTEVTITD-

GroEL_CH60_CHLPN_chlamydophila NEGYDALRDAYTDMIDAGILDPTKVTRSALESAASIAGLLLTTEALIAD-

GroEL3_chlamydia_psittaci_AEG8 NEGYDALRDAYTDMIEAGILDPTKVTRCALESAASVAGLLLTTEALIAD-

GroEL2_salmonella_enterica_typ NYGYNAATEEYGNMIDMGILDPTKVTRSALQYAASVAGLMITTECMVTD-

GroEL_salmonella_typhi_TY2_U01 NYGYNAATEEYGNMIDMGILDPTKVTRSALQYAASVAGLMITTECMVTD-

GroEL_E_coli_K12_AAC77103 NYGYNAATEEYGNMIDMGILDPTKVTRSALQYAASVAGLMITTECMVTD-

GroEL_Yersinia_pestis_NP_99189 SFGYNAYTEEYGDMIAMGILDPTKVTRSALQYAASIAGLMITTECMVTD-

GroEL_haemophilus_influenzae_Y NFGYNAGTEQYGDMIEMGILDPTKVTRSALQFAASVAGLMITTECMVTD-

GroEL_Legionella_YP_126086 NYGFNAATGEYGDMVEMGILDPTKVTRMALQNAASVASLMLTTECMVAD-

GroEL_Burkholderia_multivorans NFGYNAATGEYGDLVEAGVVDPTKVTRTALQNAASIAGLILTTDATVAD-

GroEL_Bartonella_henselae_YP_0 TFGYNTATGEFGDLIALGIVDPVKVVRSALQNAASIASLLITTEAMVAE-

GroEL_brucella_abortus_ZP_0587 TFGYNTANGEYGDLISLGIVDPVKVVRTALQNAASVAGLLITTEAMIAE-

GroEL_Mycobact_tuberc_CAA17397 GHGLNAQTGVYEDLLAAGVADPVKVTRSALQNAASIAGLFLTTEAVVAD-

GroEL_Mycobacterium_leprae_TN_ GHGLNAATGEYEDLLKAGVADPVKVTRSALQNAASIAGLFLTTEAVVAD-

GroEL_tropheryma_whipplei_NP_7 GHGLDASTGEYVDMLSRGISDPVKVTRSALENAASIAGLFLTTEAVVAE-

GroEL_Staph_aureus_MRSA_YP_041 GVGFNAATNEWVNMLEVGIVDPTKVTRSALQHAASVAAMFLTTEAVVAS-

GroEL_Listeria_monocytogenes_A GVGFNAANGEWVNMIDAGIVDPTKVTRSALQNASSVAALLLTTEAVVAD-

GroEL_streptococcus_pneumoniae GIGFNAATGEWVNMIDQGIIDPVKVSRSALQNAASVASLILTTEAVVAN-

GroEL_mycoplasma_penetrans_NP_ HLGFNAATGKYVDMFQTGIIDPVKVTRIALQNAVSVSSMLLTTEAVIYD-

HSP60_Leishmania_Strain_Friedl SFGYNAQTGEYVNMFEAGIIDPMKVVKSAVVNACSVAGMMITTEAAVVEK

HSP60_Schistosoma_mansoni_XP_0 NMGYDAQNDAYVDMIEAGIIDPTKVVRTALVDAAGVASLLTTAETVVTD-

Hsp60_human_mitochondria_NP_95 EVGYDAMAGDFVNMVEKGIIDPTKVVRTALLDAAGVASLLTTAEVVVTE-

HSP60_Plasmodium_falciparum_XP NIGFNAQEGKYVDMIESGIIDPTKVVKTAISDAASIASLMTTTEVAIVD-

chaperonin60_Cryptosporidium_p HFGFDAQTGQFVDMIESGILDPTKVVKSGLRDAASIASLMTTTQVSVFE-

Chaperonin60_Entamoeba_histoly SFGYDVRKNVYCDLMKVGIVDPTKVVRNAFNEAISVGSLIATSEALITD-

Chaperonin60_Giardia_lamblia_X FYGFDALNGQFVNMEKAGILDATKVVTTALDSALGVSSVLLNTDAVVQP-

650

GroEL_orientia_tsutsugamushi_C ----------------------HEEDNNTGNRSGGGVGGGHHGGMGGMDF

GroEL_rickettsia_bellii_YP_537 ----------------------EPEDKENPMPMRGGMGG--MGGMGGMDF

GroEL5_neoehrlichia_mikurensis --------------------------------------------------

GroEL_anaplasma_phagocytophili ----------------------VPSKNDAAGAGAGGMGG--MGGMGGF--

GroEL_ehrlichia_deer_AB454077 --------------------------------------------------

GroEL_Wolbachia_YP_198181 ----------------------VPNKEENASSSMG-AGG--MGGMNGF--

GroEL_Borrelia_garinii_YP_0730 ----------------------IKEE---KNTSGGGGYPMDPGMGMM---

GroEL_treponema_pallidum_YP_00 ----------------------IPE----KSSSTPPAPDMG-GMGGMY--

GroEL_Leptospira_interrogans_Y ----------------------KPDKDAPNPMAGMGGGGMG-GMGGMM--

GroEL_CH60_CHLPN_chlamydophila ----------------------IPEE---KSSSAPAMPSAG--MDY----

GroEL3_chlamydia_psittaci_AEG8 ----------------------IPEE---KSSSVPAMPGAG--MDY----

GroEL2_salmonella_enterica_typ ----------------------LPK-SDAPDLGAAGGMGGMGGMGGMM--

GroEL_salmonella_typhi_TY2_U01 ----------------------LPK-SDAPDLGAAGGMGGMGGMGGMM--

GroEL_E_coli_K12_AAC77103 ----------------------LPK-NDAADLGAAGGMGGMGGMGGMM--

GroEL_Yersinia_pestis_NP_99189 ----------------------LPR-DDKGADMGAGGMGGMGGMGGMM--

GroEL_haemophilus_influenzae_Y ----------------------LPK-DDKADLGAAG-MGGMGGMGGMM--

GroEL_Legionella_YP_126086 ----------------------LPKKEEGVGAGDMGGMGGMGGMGGMM--

GroEL_Burkholderia_multivorans ----------------------APK-DESAAPAPSPALDY----------

GroEL_Bartonella_henselae_YP_0 ----------------------VPKKDTPVPPMPGGGMGGMGGMDF----

GroEL_brucella_abortus_ZP_0587 ----------------------LPKKDAAPAGMPGG-MGGMGGMDF----

GroEL_Mycobact_tuberc_CAA17397 ----------------------KPEKEKA--SVPG-GGDMG-GMDF----

GroEL_Mycobacterium_leprae_TN_ ----------------------KPEKTAA--PASDPTGGMG-GMDF----

GroEL_tropheryma_whipplei_NP_7 ----------------------KPEPK----PAPGPADPGA-GMDF----

GroEL_Staph_aureus_MRSA_YP_041 ----------------------IPEKNN----DQPNMGGMP-GM------

GroEL_Listeria_monocytogenes_A ----------------------KPDENGPAAVPDMGMGGMG-GMM-----

GroEL_streptococcus_pneumoniae ----------------------KPEPVAP--APAMDPSMMG-GMM-----

GroEL_mycoplasma_penetrans_NP_ ----------------------VKD-DKEDSVPAMPNMGMG-GMM-----

HSP60_Leishmania_Strain_Friedl DLLGREKRIEDEGMEDKEKKRSVDKLRKQVNERDAPMPKMAPPMKFDMKG

HSP60_Schistosoma_mansoni_XP_0 ----------------------LPKEETGANAAGMGGMGGMGGMGGMM--

Hsp60_human_mitochondria_NP_95 ----------------------IPKEEKDPGMGAMGGMG--GGMGGGMF-

HSP60_Plasmodium_falciparum_XP ----------------------FKDSKNEESSQHMNSVNSMGDMGGMY--

chaperonin60_Cryptosporidium_p ----------------------PSNQSEKNNSSGSNSSESSSSFGSLPGD

Chaperonin60_Entamoeba_histoly ----------------------EPIKKEIN--------------------

Chaperonin60_Giardia_lamblia_X ----------------------IPTDTNLFKNK-----------------

GroEL_orientia_tsutsugamushi_C --

GroEL_rickettsia_bellii_YP_537 --

GroEL5_neoehrlichia_mikurensis --

GroEL_anaplasma_phagocytophili --

GroEL_ehrlichia_deer_AB454077 --

GroEL_Wolbachia_YP_198181 --

GroEL_Borrelia_garinii_YP_0730 --

GroEL_treponema_pallidum_YP_00 --

GroEL_Leptospira_interrogans_Y --

GroEL_CH60_CHLPN_chlamydophila --

GroEL3_chlamydia_psittaci_AEG8 --

GroEL2_salmonella_enterica_typ --

GroEL_salmonella_typhi_TY2_U01 --

GroEL_E_coli_K12_AAC77103 --

GroEL_Yersinia_pestis_NP_99189 --

GroEL_haemophilus_influenzae_Y --

GroEL_Legionella_YP_126086 --

GroEL_Burkholderia_multivorans --

GroEL_Bartonella_henselae_YP_0 --

GroEL_brucella_abortus_ZP_0587 --

GroEL_Mycobact_tuberc_CAA17397 --

GroEL_Mycobacterium_leprae_TN_ --

GroEL_tropheryma_whipplei_NP_7 --

GroEL_Staph_aureus_MRSA_YP_041 --

GroEL_Listeria_monocytogenes_A --

GroEL_streptococcus_pneumoniae --

GroEL_mycoplasma_penetrans_NP_ --

HSP60_Leishmania_Strain_Friedl L-

HSP60_Schistosoma_mansoni_XP_0 --

Hsp60_human_mitochondria_NP_95 --

HSP60_Plasmodium_falciparum_XP --

chaperonin60_Cryptosporidium_p FY

Chaperonin60_Entamoeba_histoly --

Chaperonin60_Giardia_lamblia_X --

**Section 6. Further discussion on HSP60 epitopes**

Antibodies to certain peptides from human HSP60 (not overlapping the ones preferentially recognized by antibodies in ME samples described here) are also more common in diabetes type 1 compared to controls ([*17*](#_ENREF_17)).

As shown in Figure 5, several of the chlamydia HSP60 epitopes defined with shorter synthetic peptides ([*8*](#_ENREF_8)*,* [*18-20*](#_ENREF_18)) were also seen in our epitope survey. Compared to controls, secretory IgA reactive with a chlamydia peptide (ATLVGNRIRGGF) was more common in women with infertility ([*20*](#_ENREF_20)) and was more commonly recognized by IgG from women with pelvic inflammatory disease ([*18*](#_ENREF_18)). It overlapped our human HSP60 peptides G20 and G20c and their microbial homologs. However it contained only two amino acids (underlined) of the consensus antigenicity profile defined here. Although unlikely, its cross-reactions with G20c homologs and ME samples should be evaluated. Women with *Chlamydia trachomatis*-associated ectopic pregnancy recognized 13 peptides from *Chlamydia trachomatis* HSP60 ([*8*](#_ENREF_8)*,* [*19*](#_ENREF_19)) (marked in the alignment of Supplementary Figure SF2). Our longer peptides detected some, but not all, of these epitopes, using BD and ME patient samples. It remains to investigate if hidden in these reactions lie diagnostically useful epitopes, and whether there exist disease-specific *Chlamydia pneumoniae* HSP60 antibody patterns with our set of peptides, aside from the selective ME reactions reported here.

Knowing that HSP60 is a highly conserved and cross-reactive protein, the ability of some *Chlamydia pneumoniae*, and *Mycoplasma penetrans* HSP60 peptides to selectively detect IgM antibodies in blood samples from ME patients cannot be taken as definite evidence for *Chlamydia* or *Mycoplasma* as etiologic agents in ME. The matter requires an investigation with more HSP60 antigens than was possible in this report.

*Other disease associations of antibodies reacting with HSP60 peptides, and their crossreactivities.*

In the mycobacterially induced adjuvant arthritis of rats, the arthritis starts a T cell response to a centrally placed HSP60 epitope which subsides when a response to the C terminal portion of HSP65 occurs ([*21*](#_ENREF_21)). The humoral anti-HSP60 response appears to protect against the arthritis ([*22*](#_ENREF_22)). The antibody response initially involves several epitopes, but later narrows down to fewer epitopes. This is reminiscent of the epitope differences in the IgM and IgG tests seen in this work, where IgM epitopes were more numerous than the IgG ones. The *Porphyromonas gingivalis* peptide 19 (*TLVVNRLRG*SLKICAVKAPG) was frequently antigenic in patients suffering from cardiovascular disease ([*5*](#_ENREF_5)*,* [*23*](#_ENREF_23)). It contains nine (underlined) of the 16 consensus amino acids critical for detection of antibodies in some ME patients defined here. It contains a part of apical helix I (italics). The possibility that peptide p19 and the G20/G20c homologs share some antigenicity should be tested experimentally. Antibodies to certain peptides from human HSP60 are also more common in T1D compared to controls ([*17*](#_ENREF_17)). There exists a correlation between enterovirus infection and T1D, see e.g. ([*24*](#_ENREF_24)*,* [*25*](#_ENREF_25)). Of note, antibodies to HSP60 peptides were also induced after enterovirus infection ([*25*](#_ENREF_25)).

**Section 7. Further details regarding neurological autoimmune diseases**

A large number of neurological diseases, like narcolepsy ([*26-29*](#_ENREF_26)), postinfectious encephalomyelitis, including the Guillain-Barré syndrome ([*30*](#_ENREF_30)), and pediatric neuropsychiatric disorder with tics and Sydenham´s chorea after streptococcal infection ([*31-33*](#_ENREF_31)) are now known. Other diseases with autoimmunity to specific brain tissues are multiple sclerosis ([*34*](#_ENREF_34)) and anti-NMDA receptor encephalitis ([*35*](#_ENREF_35)). It is relevant for this paper that IgM autoepitopes of human HSP60 and human HSP70 proteins are commonly recognized in MS ([*36*](#_ENREF_36)*,* [*37*](#_ENREF_37)).

**Section 8. Further details regarding the association of Chlamydia and Mycoplasma with ME.**

The literature contains conflicting results regarding *Chlamydia* and *Mycoplasma* as contributing factors in the etiology of ME ([*38-48*](#_ENREF_38)).

**Section 9. Other supplementary information**

**Supplementary Figure SF6.** Needle plots of *Chlamydia pneumoniae* HSP60 peptides with significant reactivity and selectivity for ME. Results with peptides coupled with short and long spacer, IgG and IgM are shown. Abscissas: Patient categories as detailed in the legend of Figure 1. Ordinates: MFI.

**
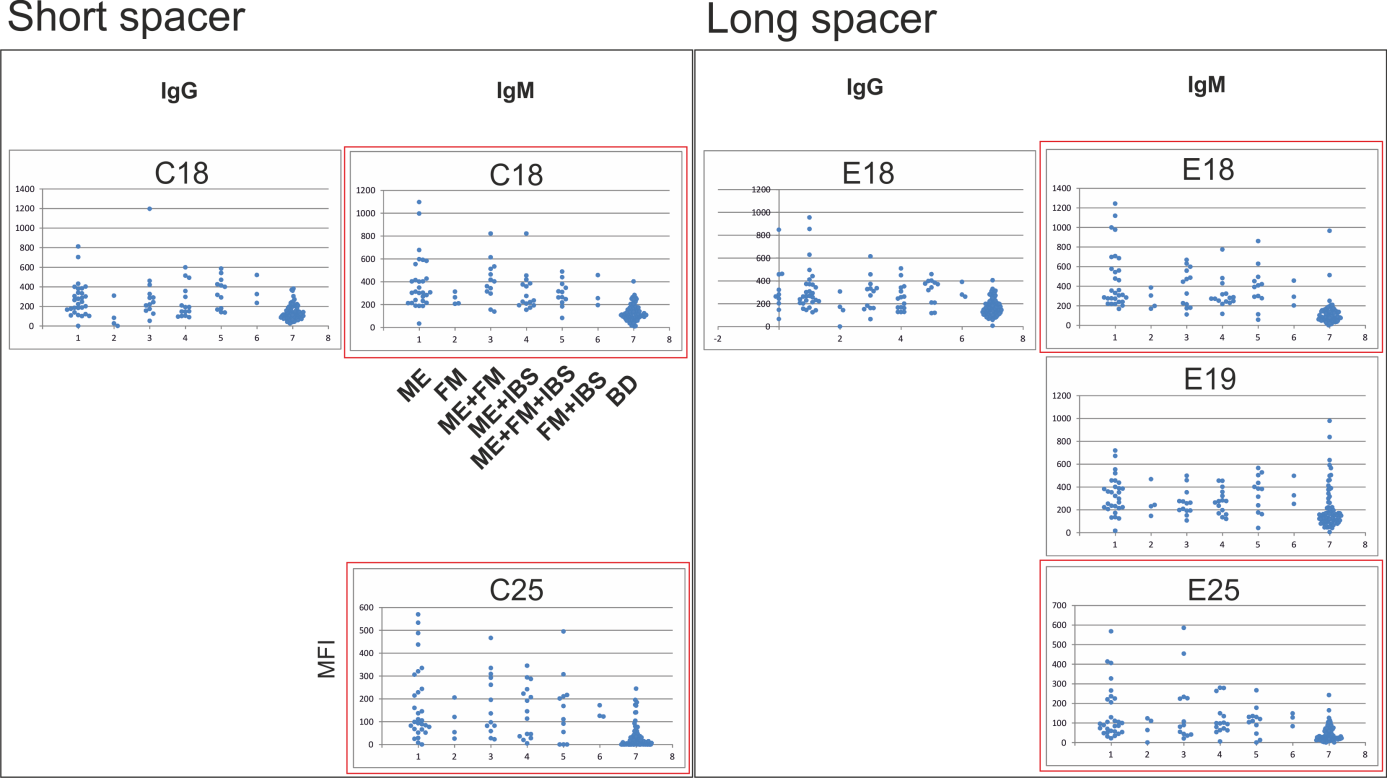
**

**Supplementary figure SF7.** Epitope scanning with overlapping HSP60 peptides from *Mycoplasma penetrans.*


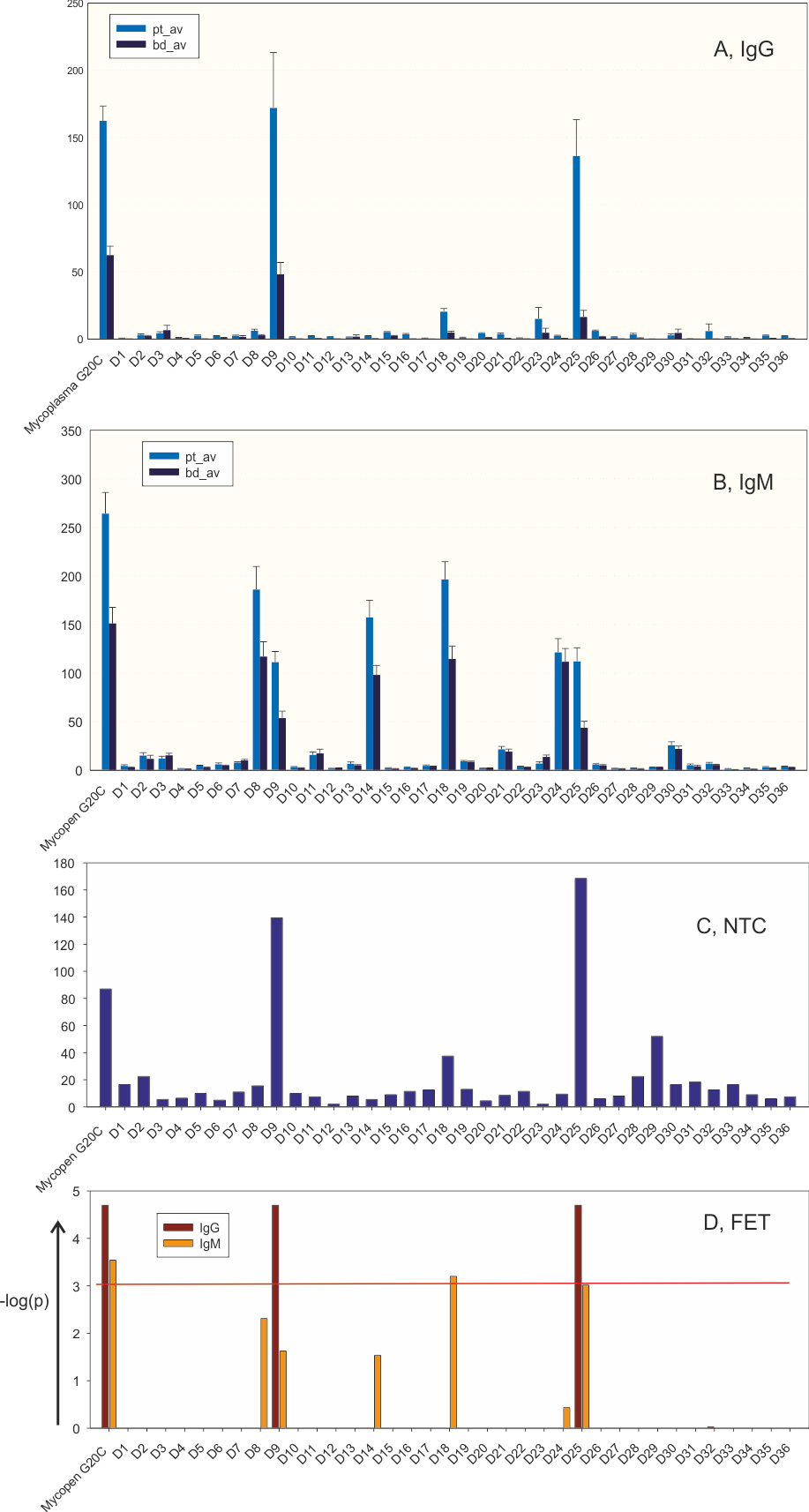


**Supplementary figure SF8.**

Needle plots of antigenicity of the most discriminatory *Mycoplasma penetrans* peptides separated according to Test set patient category and BD status (cf Figure SF3). Ordinates: MFI. Abscissae: Categories. A. IgG data. B. IgM data.


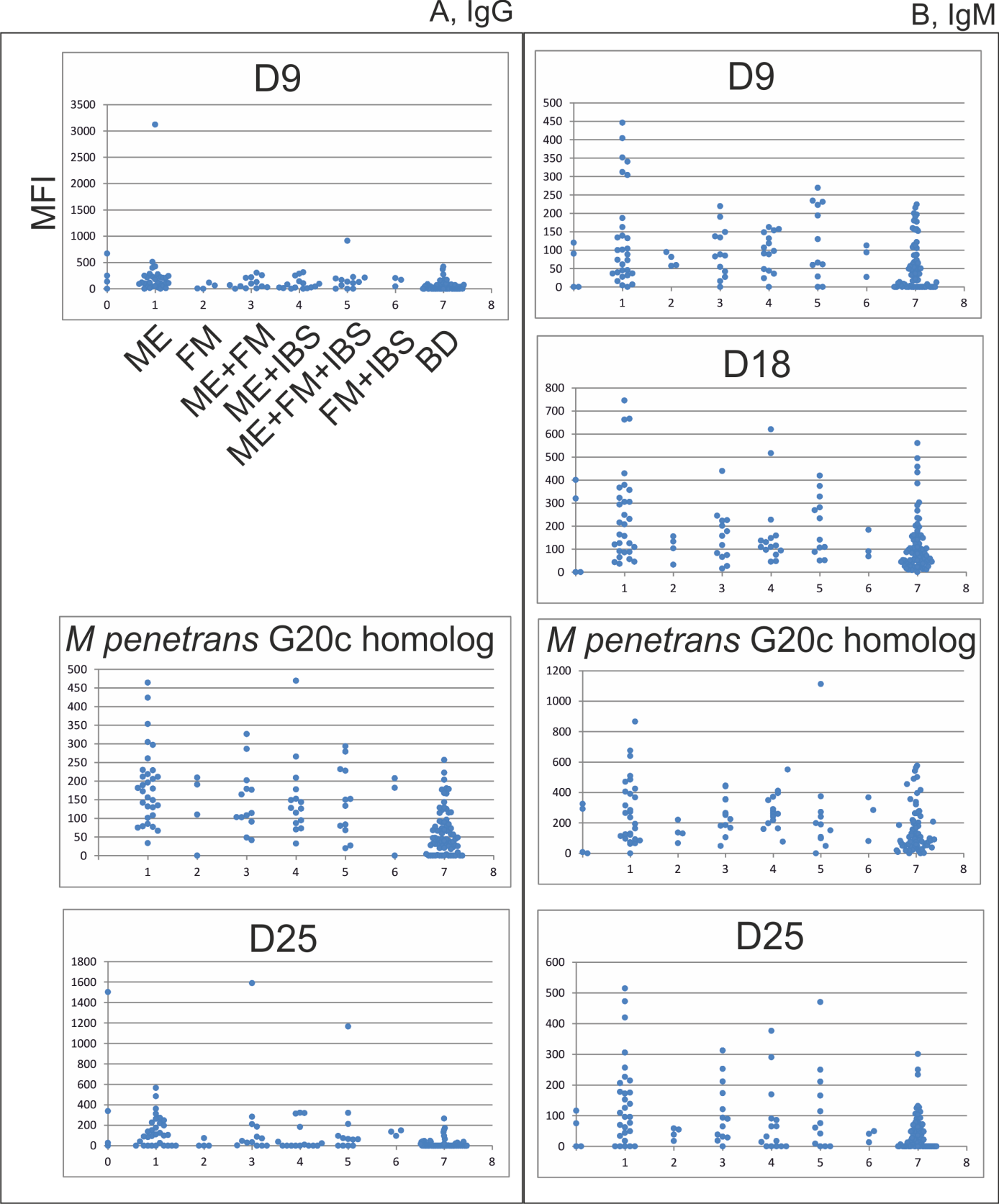


The pattern for IgG reactivity of the *Mycoplasma* HSP60 peptides was somewhat different from that of the *Chlamydia* HSP60 peptides, with D18 (EALTTLVVNKMRGVFNVVAVKAPEFGDKRK, Supplementary Table ST4) being largely non-reactive. However, the separately synthesized *Mycoplasma penetrans* G20c homolog (LTTLVVNKMRGVFNVVAVKAPEFGDKRKQV, Supplementary Table ST3) reacted with a strong ME sample preference. The D18 peptide contained the whole apical helix I sequence (underlined) and the G20c homolog contained most of it.

**Supplementary table ST8.** Panel of antigen candidates.

| **Short name** | **Antigen type** | **Origin of HSP60** |
| --- | --- | --- |
| P5 | G20c homolog peptide | *Borrelia gariini* |
| P7 | “ | *Tropheryma whipplei* |
| P11 | “ | *Escherichia coli* |
| P12 | “ | *Chlamydia pneumoniae* |
| P13 | “ | *Staphylococcus aureus* |
| P15 | “ | *Mycoplasma penetrans* |
| P16 | “ | *Leishmania major* |
| P17 | “ | *Schistosoma mansonii* |
| P18 | “ | *Plasmodium falciparum* |
| P19 | “ | *Leptospira interrogans* |
| P20 | “ | *Listeria monocytogenes* |
| P22 | “ | *Treponema pallidum* |
| P24 | “ | *Cryptosporidium parvum* |
| P25 | “ | *Entamoeba histolytica* |
| D9 | Peptide from N terminal half | *Mycoplasma penetrans* |
| D25 | Peptide from C terminal half | *Mycoplasma penetrans* |
| C18 | Peptide overlapping G20c, short spacer | *Chlamydia pneumoniae* |
| C25 | Peptide from C terminal half, short spacer | *Chlamydia pneumoniae* |
| C29 | Peptide from C terminal half, short spacer | *Chlamydia pneumoniae* |
| E18 | Peptide overlapping g20c, long spacer | *Chlamydia pneumoniae* |
| E25 | Peptide from C terminal half, long spacer | *Chlamydia pneumoniae* |
| G20 | Peptide overlapping g20c | *Homo sapiens* |
| G20c | g20c homolog peptide | *Homo sapiens* |
| Human HSP60 | Recombinant human HSP60 | *Homo sapiens* |
| E coli HSP60 | Recombinant *E coli* HSP60 (“GroEL”) | *Escherichia coli* |

**Results from the evaluation stage**

**Figure SF9.** Overview of the results from the evaluation stage

Figure SF9 depicts the result of the IgM test of the selected antigens with the Evaluation sample set. Blood donors from 2010 (n=91) gave lower values than the other blood donor groups (BD2005, n=50, and BD2013, n=161) for all antigens. ME (n=61), MS (n=20) and SLE (n=48) samples were included. The differences between ME and BD observed in the Test set, where a different set of blood donors from 2010 was used, could not be reproduced in the Evaluation set, with the exception of the P12 (*Chlamydia pneumoniae* G20c homolog) antigen. Thus, the date of blood donation influenced the Test set results.

We wanted to test the hypothesis that the IgM reactivity of the p12 peptide simply reflected the presence of IgM reactive with whole *Chlamydia pneumoniae* antigen. As seen in supplementary figure SF10 A the reactivity of this antigen was not higher in ME patients than in MS and SLE patients and blood donors. The whole antigen relative MFI values did not correlate with p12 relative MFI. Supplementary figure SF10 B shows that there is no evidence for a correlation of these two serological variables.

**Figure SF10.** Results with elementary body *Chlamydia pneumoniae* antigen. A. Needle plot of MFI for the categories ME, MS, SLE and BD. B. Scatter plot of MFI of Chlamydia antigen versus that of peptide p12 (*Chlamydia* homolog of G20c).


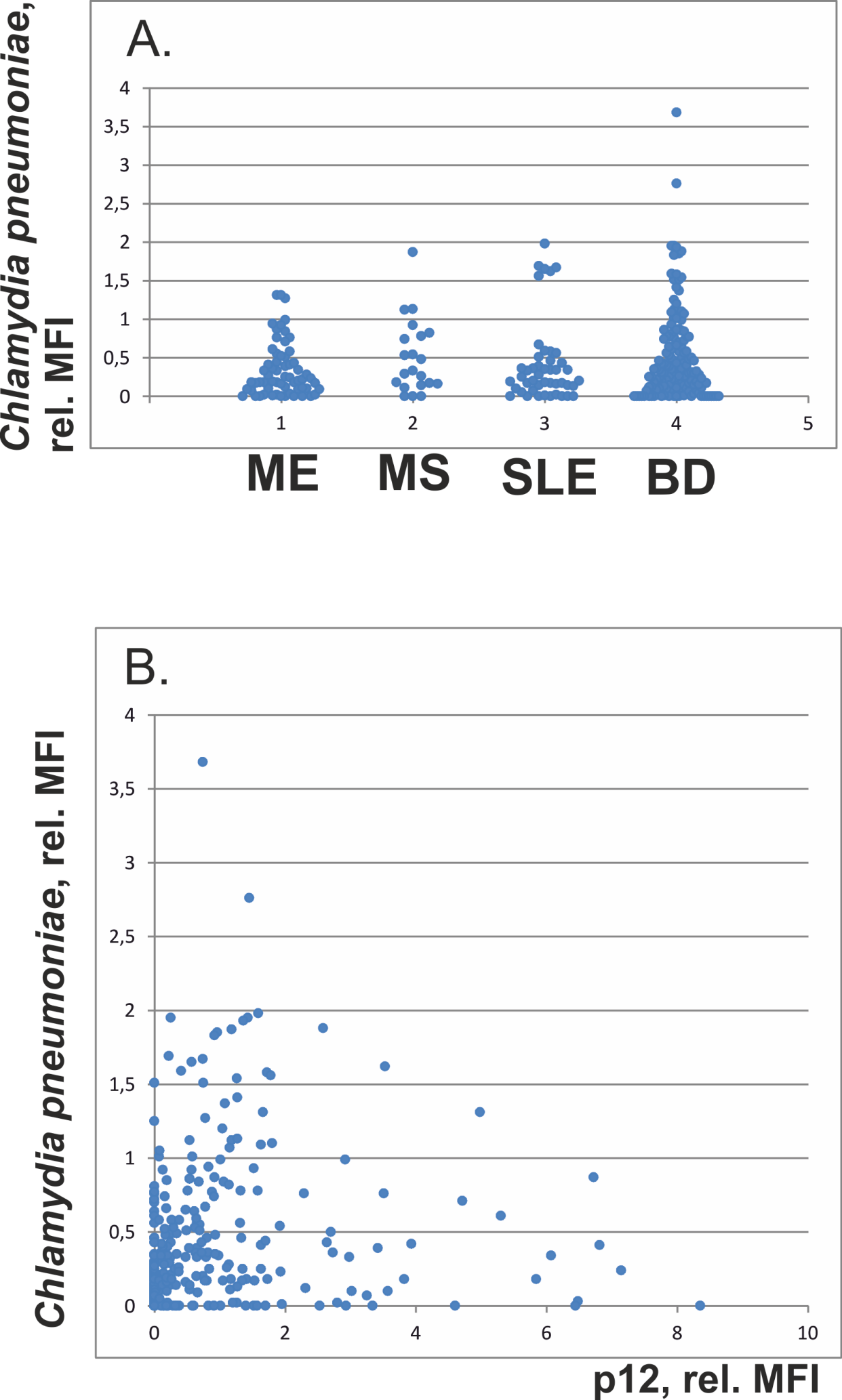


**Supplementary figure SF11**. ROC curve for discrimination of ME from BD by p12 and IgM in the Evaluation set. Two breakpoints were calculated, either with a relative MFI of 1, optimizing for sensitivity, or a relative MFI of 3, optimizing for positive predictive value.


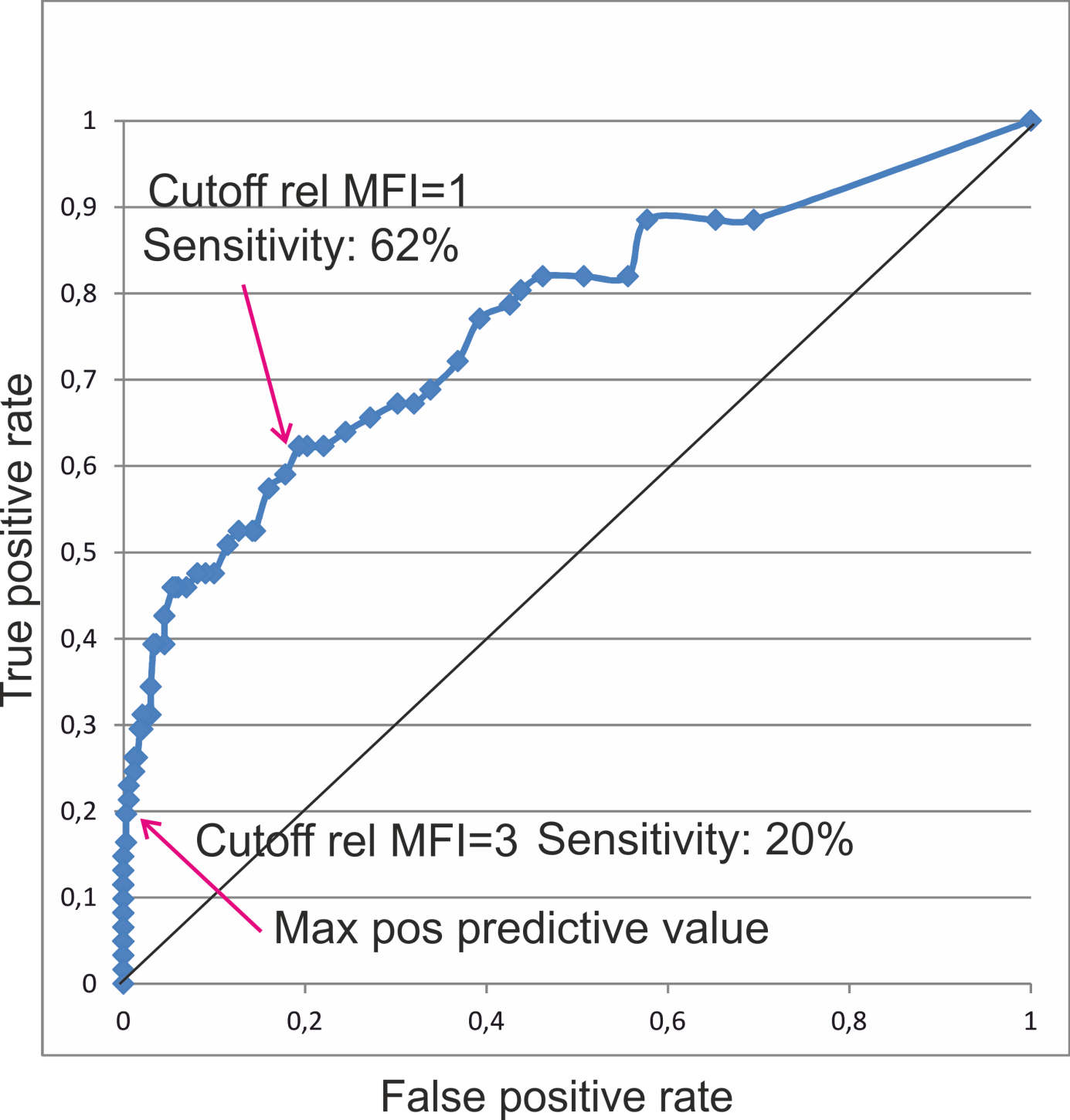


References

1. L. Chen, P. B. Sigler, The crystal structure of a GroEL/peptide complex: plasticity as a basis for substrate diversity. *Cell* **99**, 757 (Dec 23, 1999).

2. H. Perschinka *et al.*, Cross-reactive B-cell epitopes of microbial and human heat shock protein 60/65 in atherosclerosis. *Arterioscler Thromb Vasc Biol* **23**, 1060 (Jun 1, 2003).

3. E. Jeong, J. Y. Lee, S. J. Kim, J. Choi, Predominant immunoreactivity of Porphyromonas gingivalis heat shock protein in autoimmune diseases. *J Periodontal Res* **47**, 811 (Dec, 2012).

4. T. Okada *et al.*, Antibodies against heat shock protein 60 derived from Helicobacter pylori: diagnostic implications in cardiovascular disease. *Journal of autoimmunity* **29**, 106 (Sep-Nov, 2007).

5. J. Wysocki *et al.*, Human heat shock protein 60 (409-424) fragment is recognized by serum antibodies of patients with acute coronary syndromes. *Cardiovasc Pathol* **11**, 238 (Jul-Aug, 2002).

6. J. J. Hansen *et al.*, Hereditary spastic paraplegia SPG13 is associated with a mutation in the gene encoding the mitochondrial chaperonin Hsp60. *Am J Hum Genet* **70**, 1328 (May, 2002).

7. T. Harkonen *et al.*, Picornavirus proteins share antigenic determinants with heat shock proteins 60/65. *J Med Virol* **62**, 383 (Nov, 2000).

8. Y. Yi, G. Zhong, R. C. Brunham, Continuous B-cell epitopes in Chlamydia trachomatis heat shock protein 60. *Infect Immun* **61**, 1117 (Mar, 1993).

9. Y. Xue, M. L. O'Mara, P. P. Surawski, M. Trau, A. E. Mark, Effect of poly(ethylene glycol) (PEG) spacers on the conformational properties of small peptides: a molecular dynamics study. *Langmuir* **27**, 296 (Jan 4, 2011).

10. J. Blomberg *et al.*, No evidence for xenotropic murine leukemia-related virus infection in Sweden using internally controlled multiepitope suspension array serology. *Clin Vaccine Immunol* **19**, 1399 (Sep, 2012).

11. A. Sheikholvaezin, F. Blomberg, C. Ohrmalm, A. Sjosten, J. Blomberg, Rational recombinant XMRV antigen preparation and bead coupling for multiplex serology in a suspension array. *Protein Expr Purif* **80**, 176 (Dec, 2011).

12. B. Akerstrom, T. Brodin, K. Reis, L. Bjorck, Protein G: a powerful tool for binding and detection of monoclonal and polyclonal antibodies. *J Immunol* **135**, 2589 (Oct, 1985).

13. L. Bjorck, J. Blomberg, Streptococcal protein G: a sensitive tool for detection of antibodies to human immunodeficiency virus proteins in Western blot analysis. *Eur J Clin Microbiol* **6**, 428 (Aug, 1987).

14. J. Michaelsson *et al.*, A signal peptide derived from hsp60 binds HLA-E and interferes with CD94/NKG2A recognition. *J Exp Med* **196**, 1403 (Dec 2, 2002).

15. R. Lachumanan, S. Devi, Y. M. Cheong, S. J. Rodda, T. Pang, Epitope mapping of the Sta58 major outer membrane protein of Rickettsia tsutsugamushi. *Infect Immun* **61**, 4527 (Oct, 1993).

16. K. Braig *et al.*, The crystal structure of the bacterial chaperonin GroEL at 2.8 A. *Nature* **371**, 578 (Oct 13, 1994).

17. L. Horvath *et al.*, Antibodies against different epitopes of heat-shock protein 60 in children with type 1 diabetes mellitus. *Immunol Lett* **80**, 155 (Mar 1, 2002).

18. M. Domeika, K. Domeika, J. Paavonen, P. A. Mardh, S. S. Witkin, Humoral immune response to conserved epitopes of Chlamydia trachomatis and human 60-kDa heat-shock protein in women with pelvic inflammatory disease. *J Infect Dis* **177**, 714 (Mar, 1998).

19. I. Sziller *et al.*, Serological responses of patients with ectopic pregnancy to epitopes of the Chlamydia trachomatis 60 kDa heat shock protein. *Hum Reprod* **13**, 1088 (Apr, 1998).

20. I. Kligman, J. A. Grifo, S. S. Witkin, Expression of the 60 kDa heat shock protein in peritoneal fluids from women with endometriosis: implications for endometriosis-associated infertility. *Hum Reprod* **11**, 2736 (Dec, 1996).

21. K. D. Moudgil, M. Durai, Regulation of autoimmune arthritis by self-heat-shock proteins. *Trends Immunol* **29**, 412 (Sep, 2008).

22. H. R. Kim, E. Y. Kim, J. Cerny, K. D. Moudgil, Antibody responses to mycobacterial and self heat shock protein 65 in autoimmune arthritis: epitope specificity and implication in pathogenesis. *J Immunol* **177**, 6634 (Nov 15, 2006).

23. H. Perschinka *et al.*, Identification of atherosclerosis-associated conformational heat shock protein 60 epitopes by phage display and structural alignment. *Atherosclerosis* **194**, 79 (Sep, 2007).

24. A. Elfaitouri *et al.*, Recent enterovirus infection in type 1 diabetes: evidence with a novel IgM method. *J Med Virol* **79**, 1861 (Dec, 2007).

25. T. Harkonen *et al.*, Enterovirus infection may induce humoral immune response reacting with islet cell autoantigens in humans. *J Med Virol* **69**, 426 (Mar, 2003).

26. B. R. Kornum, J. Faraco, E. Mignot, Narcolepsy with hypocretin/orexin deficiency, infections and autoimmunity of the brain. *Curr Opin Neurobiol* **21**, 897 (Dec, 2011).

27. B. R. Kornum *et al.*, Common variants in P2RY11 are associated with narcolepsy. *Nat Genet* **43**, 66 (Jan, 2011).

28. J. Hallmayer *et al.*, Narcolepsy is strongly associated with the T-cell receptor alpha locus. *Nat Genet* **41**, 708 (Jun, 2009).

29. A. Fontana *et al.*, Narcolepsy: autoimmunity, effector T cell activation due to infection, or T cell independent, major histocompatibility complex class II induced neuronal loss? *Brain* **133**, 1300 (May, 2010).

30. D. M. Wingerchuk, Postinfectious encephalomyelitis. *Curr Neurol Neurosci Rep* **3**, 256 (May, 2003).

31. R. Kurlan, E. L. Kaplan, The pediatric autoimmune neuropsychiatric disorders associated with streptococcal infection (PANDAS) etiology for tics and obsessive-compulsive symptoms: hypothesis or entity? Practical considerations for the clinician. *Pediatrics* **113**, 883 (Apr, 2004).

32. T. K. Murphy, R. Kurlan, J. Leckman, The immunobiology of Tourette's disorder, pediatric autoimmune neuropsychiatric disorders associated with Streptococcus, and related disorders: a way forward. *J Child Adolesc Psychopharmacol* **20**, 317 (Aug, 2010).

33. J. F. Baizabal-Carvallo, J. Jankovic, Movement disorders in autoimmune diseases. *Mov Disord* **27**, 935 (Jul, 2012).

34. M. Comabella, S. J. Khoury, Immunopathogenesis of multiple sclerosis. *Clin Immunol* **142**, 2 (Jan, 2012).

35. F. Piehl, K. Winnerback, [Newly discovered autoimmune encephalitis wrongly mistaken for mental disease. Anti-NMDA receptor encephalitis puts demands on correct differential diagnosis]. *Lakartidningen* **107**, 589 (Mar 3-9, 2010).

36. M. J. Mansilla, X. Montalban, C. Espejo, Heat shock protein 70: roles in multiple sclerosis. *Mol Med* **18**, 1018 (2012).

37. F. J. Quintana *et al.*, Antigen microarrays identify unique serum autoantibody signatures in clinical and pathologic subtypes of multiple sclerosis. *Proc Natl Acad Sci U S A* **105**, 18889 (Dec 2, 2008).

38. G. L. Nicolson, R. Gan, J. Haier, Multiple co-infections (Mycoplasma, Chlamydia, human herpes virus-6) in blood of chronic fatigue syndrome patients: association with signs and symptoms. *Apmis* **111**, 557 (May, 2003).

39. S. D. Vernon, S. K. Shukla, W. C. Reeves, Absence of Mycoplasma species DNA in chronic fatigue syndrome. *J Med Microbiol* **52**, 1027 (Nov, 2003).

40. G. K. Endresen, Mycoplasma blood infection in chronic fatigue and fibromyalgia syndromes. *Rheumatol Int* **23**, 211 (Sep, 2003).

41. J. Nijs, G. L. Nicolson, P. De Becker, D. Coomans, K. De Meirleir, High prevalence of Mycoplasma infections among European chronic fatigue syndrome patients. Examination of four Mycoplasma species in blood of chronic fatigue syndrome patients. *FEMS Immunol Med Microbiol* **34**, 209 (Nov 15, 2002).

42. M. Nasralla, J. Haier, G. L. Nicolson, Multiple mycoplasmal infections detected in blood of patients with chronic fatigue syndrome and/or fibromyalgia syndrome. *Eur J Clin Microbiol Infect Dis* **18**, 859 (Dec, 1999).

43. A. Vojdani *et al.*, Detection of Mycoplasma genus and Mycoplasma fermentans by PCR in patients with Chronic Fatigue Syndrome. *FEMS Immunol Med Microbiol* **22**, 355 (Dec, 1998).

44. A. L. Komaroff, D. S. Bell, P. R. Cheney, S. C. Lo, Absence of antibody to Mycoplasma fermentans in patients with chronic fatigue syndrome. *Clin Infect Dis* **17**, 1074 (Dec, 1993).

45. J. K. Chia, L. Y. Chia, Chronic Chlamydia pneumoniae infection: a treatable cause of chronic fatigue syndrome. *Clin Infect Dis* **29**, 452 (Aug, 1999).

46. L. D. Devanur, J. R. Kerr, Chronic fatigue syndrome. *J Clin Virol* **37**, 139 (Nov, 2006).

47. J. E. Greenlee, J. W. Rose, Controversies in neurological infectious diseases. *Semin Neurol* **20**, 375 (2000).

48. A. L. Komaroff, S. P. Wang, J. Lee, J. T. Grayston, No association of chronic Chlamydia pneumoniae infection with chronic fatigue syndrome. *J Infect Dis* **165**, 184 (Jan, 1992).
